# Semiautomated Synthesis and Interactive Visualization of Zeolitic Imidazolate Framework-Based Biocomposites

**DOI:** 10.1021/acs.chemmater.6c01702

**Published:** 2026-09-29

**Authors:** Xue Chen, Aleksandar Kondinski, Jing Yang, Helmar Wiltsche, Bernd Nidetzky, Francesco Carraro, Paolo Falcaro

**Affiliations:** † Institute of Physical and Theoretical Chemistry, Graz University of Technology, Stremayrgasse 9, Graz 8010, Austria; ‡ Institute of Analytical Chemistry and Food Chemistry, Graz University of Technology, Stremayrgasse 9, Graz 8010, Austria; § Institute of Biotechnology and Biochemical Engineering, Graz University of Technology, Petersgasse 12/1, Graz 8010, Austria

## Abstract

Zeolitic imidazolate framework (ZIF) biocomposites are widely studied for their potential in biocatalysis and biomedicine. However, their preparation is highly sensitive to precursor composition, concentration, washing conditions, and operator-dependent handling procedures. Here, we combine a semiautomated syringe-pump system, high-throughput sample preparation, and digital data visualization to map synthesis–structure–property relationships in ZIF biocomposites. A programmable four-syringe pump platform was used to systematically prepare 360 distinct samples in triplicate, yielding 1080 samples. The investigated synthetic parameters were relative amounts of metal, ligand, protein, the total precursor concentration, and the washing protocol. Structural, chemical, and functional characterization yielded a data set of ∼2250 entries comprising crystalline-to-amorphous ratios, IR-based protein-to-framework ratios, encapsulation efficiency, and loading capacity. We note that exploring such a large data set manually can be challenging and that current static visualizations can hide meaningful trends. With the aim of enabling a customized and interactive exploration of the data, we designed the ZIF Biocomposite Explorer App (ZIF-BEA), which supports user-defined navigation in the space of the variables, providing direct access to experimental data and material properties. We anticipate that integrating large experimental data sets with interactive digital platforms may expedite the design of MOF-based biocomposites, while providing a transferable tool for the exploration of other classes of functional materials.

## Graphical abstract

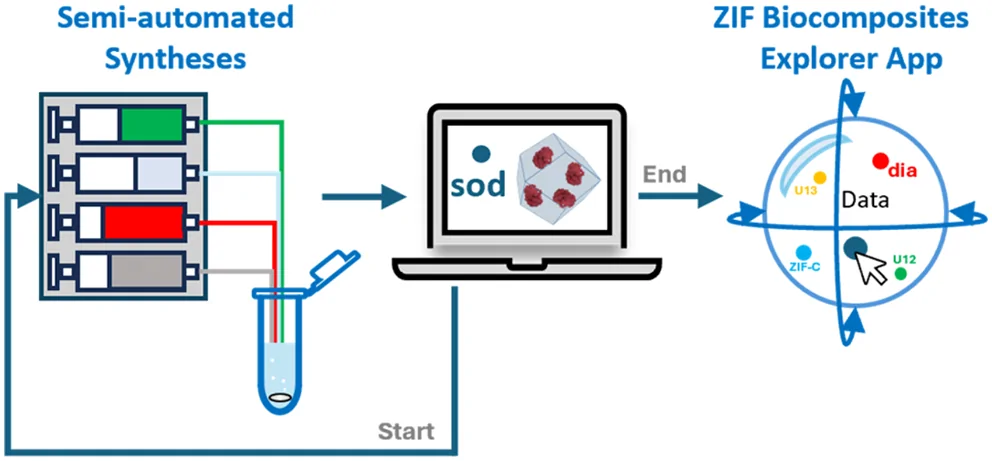

## 1 Introduction

Metal–organic frameworks (MOFs) are extended network materials formed by the self-assembly of metal-based nodes (metal ions or clusters) with multidentate organic linkers.^1^ The integration of biomacromolecules, such as proteins,^2^ carbohydrates,^3^ nucleic acids,^4,5^ and more complex biological entities such as viruses^6^ and living cells,^7^ with MOF materials yields MOF biocomposites. In these composites, the MOF coating enhances the stability of the encapsulated biomacromolecule against chemical, physical, and mechanical stressors.^8^ In addition, because several MOF matrices can be decomposed under controlled chemical conditions, these framework materials can be utilized as carriers for triggered biomolecule release.^8–10^ The combination of these properties has positioned MOF biocomposites as a promising platform for biotechnological applications such as biocatalysis^11–15^ and drug delivery.^16–20^

Among the numerous different MOFs, zeolitic imidazolate frameworks (ZIFs) have emerged as a particularly attractive class of hosts for biomacromolecule encapsulation.^21–23^ ZIF biocomposites are generally synthesized by biomimetic mineralization, a process in which biomacromolecules trigger nucleation and growth of the ZIF materials at room temperature under aqueous conditions.^2^ This approach has been widely explored with Zn salts and 2-methylimidazole (HmIM) precursors, yielding Zn­(mIM)_2_ frameworks often described as zeolitic imidazolate framework-8 (ZIF-8) systems. However, the reaction between Zn^2+^ and 2-methylimidazolate (mIM^–^) does not produce a single isostructural or isocompositional material. Depending on synthesis and processing conditions, the same precursors can yield different polymorphs, including crystalline structures of Zn­(mIM)_2_ such as sodalite (**sod**), diamondoid (**dia**), katsenite (**kat**), **ZIF-L**, and chemically distinct crystalline phases (e.g., **ZIF-C**, **ZIF-EC-1**), as well as amorphous materials, highly defective phases, or a combination thereof.^24–33^ The structural and chemical variations of these zinc-imidazolate systems strongly influence the functional properties of the resulting biocomposite, including mass transfer, encapsulation efficiency (EE%), loading capacity (LC%), stability, cytotoxicity, release kinetics, and retained bioactivity after release.^24–27^ Establishing reliable synthesis–structure–property relationships is therefore essential for the rational design of zinc-imidazolate-based biocomposites.

Despite the rapid expansion of the literature on protein@ZIF-8, defining predictive relationships among synthesis conditions, the resulting structures, and the final functional properties remains challenging. One central reason is that ZIF biocomposite formation can be rapid (the particle formation occurs within milliseconds),^34^ kinetically controlled,^35–37^ and often highly sensitive to minimal variations in experimental execution.^2,38,39^ Minor variations in precursor concentration, precursor ratio, mixing sequence, and addition rate can alter phase composition, crystallinity, encapsulation efficiency, and loading capacity.^23,25,40–44^ For example, in our laboratory, we noted that even operator-dependent factors, such as pipetting timing and mixing consistency, can significantly influence the properties of the resulting solid products, especially near phase-transition regions where small variations shift phase composition. As a result, nominally similar protocols can yield different products: this variability can limit reproducibility and complicate comparisons across studies. A second limitation is that most reports investigate only small and often discontinuous regions of multidimensional synthesis parameter space. This combination of kinetic sensitivity, operator dependence, and sparse data exploitation has made the design of customized ZIF biocomposites predominantly empirical. Large, systematic, and reproducible data sets are desired to move research in ZIF biocomposites from trial-and-error to data-assisted synthesis.

Recent works have shown that broad and systematic exploration of the variable space can reveal strong links between precursor composition, phase selection, encapsulation, loading, bioactivity, and release behavior, and that such data sets can provide practical structure–property guidance for protein@ZIF systems.^13,14,25,40,41^ Mechanistic studies have further shown that nucleation pathways, protein effects, and carbonate sources can influence ZIF phase formation, which may explain why minor changes in preparation conditions result in different phases (e.g., **ZIF-C**, **sod**, and **dia**) as well as variations in encapsulation efficiency and loading capacity.^43–45^ In the report by Hafner et al.,^26^ precursor ratio, total concentration (TC), and washing procedure were screened across hundreds of samples, establishing a 4D synthesis map for protein@ZIF biocomposites. However, even when a large data set is available, its practical use can be limited by how information is generated and displayed. For example, in protein@Zn­(mIM)_2_ systems, phase information is often reduced to a dominant-phase representation in which each synthesis condition is assigned a single label and color.^26^ While this data representation is useful for a simplified overview, it inevitably compresses mixed-phase information, thus hiding gradual structural transitions. Consequently, a sample that is only slightly enriched in **sod** relative to **ZIF-C** would be displayed in the same way as a nearly phase-pure **sod** sample, whereas a neighboring point with the inverse ratio would be assigned to a different phase.^22,25^ In these data representations, a continuous transition region is converted into an artificial abrupt phase boundary. The problem becomes more severe when multiple ternary diagrams (TDs), each corresponding to a different TC, are stacked into a single multidimensional representation (e.g., a 4D-phase diagram).^26^ In static printed plots, only selected directional views are available to readers, and data points located behind the front surface are partially or completely obscured. As a result, local trajectories across the variable space, sparse transition regions, and concentration-dependent changes in phase coexistence become difficult to inspect directly, thereby limiting access to a portion of the experimental information. Moreover, such static plots cannot provide immediate access to the underlying data (e.g., diffraction patterns) or be readily extended to include chemical information, such as infrared spectra and encapsulation parameters, within the same navigable environment. The integration of these data within an interactive platform could facilitate data interpretation and improve the reproducibility of biocomposite syntheses.

Here, we address these limitations by combining semiautomated syntheses with digital chemistry to explore protein@ZIF biocomposites. Recent studies on automated and semiautomated MOF synthesis, including robotic MOF synthesis platforms and automated layer-by-layer MOF growth, have demonstrated improved control and reproducibility in MOF preparation.^46–50^ Building on this progress, we utilized a programmable four-syringe-pump setup, and we established a semiautomated synthesis protocol in which precursor delivery, timing, and mixing are controlled instrumentally, while syringe loading, sample collection, and washing remain operator-assisted (see Figure 1a). This approach reduces operator-dependent variability in repetitive dosing steps and mixing without requiring a fully automated platform. The system was used to test 360 synthesis conditions across a defined space of precursor mass ratios and TCs with each synthesis produced in triplicate. The explored variable space was designed to encompass and extend a previously explored space^26^ to enable a direct comparison of visualization approaches. The protein chosen was bovine serum albumin (BSA), a widely used model protein whose cost is compatible with the preparation of large data sets such as that generated in this study.

**Figure 1:**
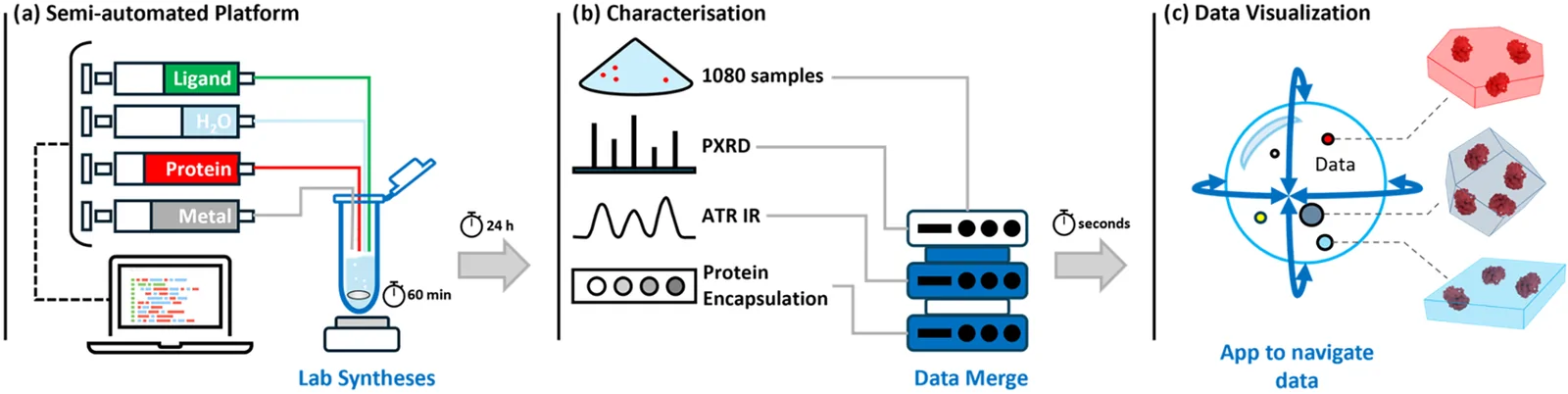
Scheme of the workflow used for the synthesis and analysis of protein@ZIF biocomposites. (a) Semiautomated synthesis platform based on software-controlled syringe pumps and mechanical stirring. (b) Characterization of 1080 samples by powder X-ray diffraction (PXRD), infrared (IR) spectroscopy, encapsulation efficiency (EE%), and loading capacity (LC%), followed by data merging with experimental synthesis conditions. (c) Data visualization of the collected data set (∼2250 entries) with a data visualization App.

The resulting materials were characterized structurally and chemically using complementary methods. Powder X-ray diffraction (PXRD) was used to determine crystalline phase composition across the data set, while infrared (IR) spectroscopy was applied to confirm protein immobilization and to evaluate the relative contribution of protein and framework components through the normalized intensity ratio between the protein (amide I vibrational mode at ∼1650 cm^–1^) and a Zn–mIM coordination band (Zn–N stretching vibration at ∼421 cm^–1^). Functional performance was assessed by quantifying EE% and LC% using Bradford colorimetric assay and atomic emission spectroscopy. Taken together, these measurements define a multidimensional synthesis–structure–chemistry–property data set rather than an isolated phase map (Figure 1b).

To enable direct exploration of this data set, we designed an interactive application that enables navigation of the mapped variable space. In contrast to static printed plots in prior reports, our data visualization platform allows the user to rotate and zoom within the data set, customize trajectories through the variable space, and focus on one phase, multiple phases, or the complete data set (Figure 1c). Selection of an individual point provides direct access to the corresponding experimental data, enabling the immediate inspection of the associated PXRD plots or IR spectra. In parallel, the application supports heat map visualization of IR-derived information across the same three-dimensional (3D) synthesis space, allowing qualitative assessment of how the relative protein/framework ratio changes with different protocols. This digital environment gives access to information that is lost or hidden in threshold-based or purely static representations, guiding users through a complex experimental space for the design of ZIF biocomposites with customized properties.

## 2 Results and Discussion

### 2.1 Semiautomated Synthesis of ZIF Biocomposites

Automation is highly desirable when consistent and precise control of liquid-based reactions is required. Examples of protocol parameters that can be controlled include reagent addition sequence, flow rate, and timing, ensuring highly reproducible preparation of large numbers of samples under well-defined conditions.^49–53^ We note that, in high-throughput chemical synthesis, in contrast to manual procedures, automation enables standardization and consistency, which improves reproducibility within a given data set and facilitates reproducible execution of protocols across different laboratories.^54^ Given this, to systematically explore the synthesis landscape of ZIF biocomposites, we developed a semiautomated synthesis platform integrating a computer-controlled programmable system^55^ with a four-syringe pump (Figure 1a) containing the separate components required for the preparation of Zn­(mIM)-based biocomposites. These include aqueous solutions of Zn­(OAc)_2_·2H_2_O (hereafter Zn^2+^), BSA, and HmIM; a fourth syringe, loaded with deionized (DI) water, was employed to regulate the reagent concentration in the final batch (further details can be found in Section 1.2, Supporting Information). Each experiment was executed from a predefined protocol list by dosing the aforementioned components, their concentrations, dosing order, dispensed volume, flow rate, and timing of each step, thereby replacing manually timed reagent addition with a scripted and repeatable synthesis procedure. Specifically, the ligand solution (L), water, BSA solution, and metal precursor solution (M) were sequentially delivered (L → water → BSA → M) using the programmable syringe-pump platform. All reagents were first added, followed by continuous magnetic stirring for 60 min to ensure homogeneous mixing. Then, the resulting suspensions were incubated under static conditions for 24 h before collection of the solid products and supernatant. This procedure replaced manually timed reagent addition with a repeatable protocol that maintained consistent dosing, timing, stirring, and incubation conditions across all syntheses (see Figures S2–S6, and Tables S7–S11, Supporting Information). All experiments were organized within a coded synthesis matrix, in which each sample was assigned a unique parameter coordinate (see Figure S1 and Tables S1–S6, Supporting Information). This coding system directly links the programmable pump protocol with the corresponding compositional variables, including precursor ratios and TC. As a result, each synthesized material can be unambiguously mapped to its synthesis parameters, enabling systematic comparison of chemical and structural outputs (material composition and crystallinity) and functional properties such as EE% and LC%, as schematically illustrated in Figure 1b. EE% represents the fraction of protein immobilized within the ZIF matrix relative to the initial amount used in the synthesis and thus provides a measure of the effectiveness of the immobilization process. LC% corresponds to the weight fraction (wt %) of protein immobilized within the ZIF matrix relative to the total mass of the biocomposite and thus provides a measure of the protein mass fraction in the final material.

Building on our previous investigation of two-dimensional (2D) TDs,^26^ the explored range of TCs was rounded from 11.2–82 to 12.5–100 mg mL^–1^. This range extended that investigated by Hafner et al.^26^ and allowed a systematic investigation of how the mass fractions of Zn­(OAc)_2_·2H_2_O (indicated as Zn^2+^), HmIM, and BSA (Zn^2+^ wt %: HmIM wt %: BSA wt %) influence the formation of ZIF biocomposites. For each TC (12.5, 25, 50, 75, and 100 mg mL^–1^), a set of TDs was constructed. Each TD consisted of 36 experimental points, in which the mass fraction of each component varied between 10 and 80 wt % (see Figure S1, Tables S1–S6, Supporting Information). To remap the effect of postsynthetic processing on phase formation, two different washing protocols were applied to each sample (see Section 1.3, Supporting Information), namely, DI water alone (here referred to as TD–H_2_O) and an initial water washing step followed by ethanol washing (here referred to as TD–EtOH). Each synthesis coordinate was prepared in both washing variants and in triplicate.

To investigate the reproducibility of the semiautomated synthesis, we compared a subset of nine samples synthesized by two different operators using micropipettes to dispense the precursor solutions and compared them with the same samples synthesized via a semiautomated setup and syringe-pump-controlled dosing. For each sample, PXRD patterns were recorded for each replicate, and the EE% was calculated by Bradford assay on the triplicates.

Overall, the comparison between manual pipet addition and syringe-pump-controlled dosing (see Figures S7–S9, Supporting Information) shows that the semiautomated preparation affords smaller variations in the crystalline ZIF phase composition and reduced relative standard deviations (RSDs) for EE% and LC%, consistent with reduced operator-dependent variability under controlled reagent delivery.

### 2.2 Sample Characterization and Data Set Generation

The crystalline structure of the dry powders was investigated by PXRD. To expand the data set of valuable properties in the explored space of the variables, here we systematically collected infrared data, EE%, and LC%. IR spectroscopy was used to confirm the protein immobilization (i.e., amide I band at ∼1650 cm^–1^) and verify the presence of ZIF fingerprint bands (e.g., Zn–N stretching band at ∼421 cm^–1^). The EE% was calculated by quantifying the protein concentration in the supernatant of each synthesis using the Bradford assay. The LC% was calculated by quantifying the S-content in the crystalline samples via an Inductively Coupled Plasma-Optical Emission Spectrometer (ICP-OES). For details about the analytical procedures, see Supporting Information Section 1.4–1.9. In summary, the data set includes 1080 PXRD patterns, 360 IR spectra, 540 EE% determinations, and 266 LC% values, totaling ∼2250 entries.

Here, to describe the effort of a classical manual interpretation of a subset of these data (105 entries, including triplicates, ∼5% of the full data set), we selected three [BSA/HmIM/Zn^2+^] composition coordinates (P31, P32, and P34) and we plotted in Figure 2 the data measured for these samples at different TCs. By analyzing the PXRD patterns of P31, P32, and P34, we further confirmed the concentration-dependent phase relationship observed by Hafner et al.,^26^ albeit with distinct details (see Tables S7–S11 for the crystalline phase composition). In P31 (Figure 2a), at low precursor concentration, the diffraction pattern is dominated by **ZIF-C**, indicating that crystallization initially proceeds via this intermediate phase. As the precursor concentration increases, diffraction peaks corresponding to the **sod** topology emerge at TC = 25 mg mL^–1^, resulting in a mixed-phase sample (average crystalline phase composition: 82.4% **ZIF-C** and 17.6% **sod**). At the highest concentration, the **sod** phase becomes dominant (100%), indicating a gradual phase transformation from **ZIF-C** to **sod** with increasing TC.

**Figure 2:**
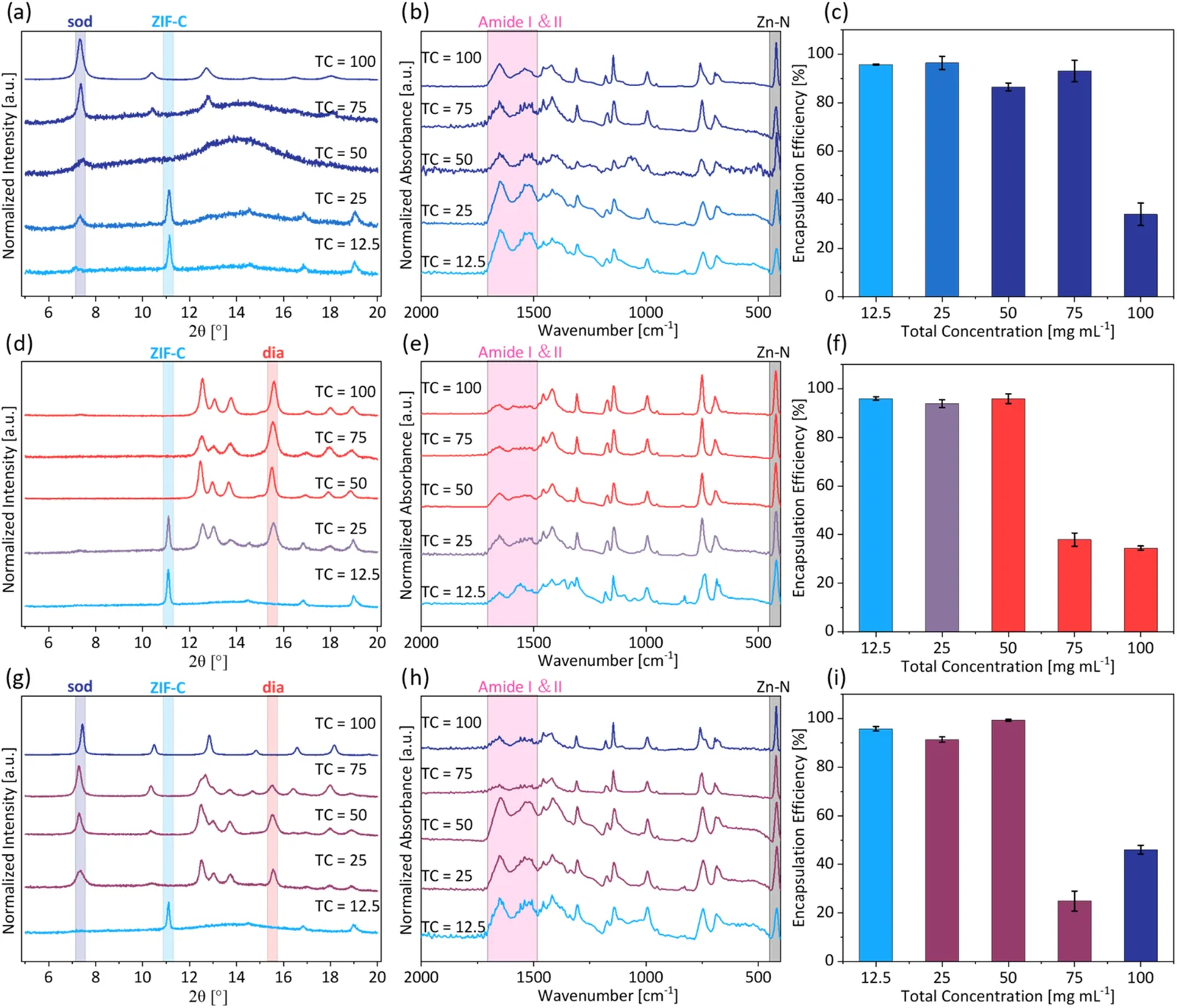
PXRD, IR, and EE% of protein@ZIF biocomposites as a function of 5 different total concentrations (TCs from 12.5 to 100 mg mL^–1^) for 3 different points (P31, P32, and P34). These samples are part of the TD–H_2_O system. (a) PXRD patterns, (b) IR spectra, and (c) EE% of the P31 sample. (d) PXRD patterns, (e) IR spectra, and (f) EE% of the P32 sample. (g) PXRD patterns, (h) IR spectra, and (i) EE% of the P34 sample.

In P32 (Figure 2d), an analogous concentration-dependent trend is observed, but with a different final topology. **ZIF-C** is initially the dominant phase (100%), followed by the appearance of a mixed **ZIF-C**/**dia** region at TC = 25 mg mL^–1^ (46.0% **ZIF-C**, 54.02% **dia**) and ultimately by a transition to a **dia**-dominated structure (100%) at higher concentrations.

The P34 sample (Figure 2g) exhibits multiple competing phases. At the lower precursor concentration (TC = 12.5 mg mL^–1^), **ZIF-C** is the dominant phase. With increasing concentration, both **dia** and **sod** phases emerge. At TC = 25 mg mL^–1^, the phase fractions are 75.7% **dia** and 24.3% **sod**; at TC = 50 mg mL^–1^, 74.0% **dia** and 26.0% **sod**; and at TC = 75 mg mL^–1^, 40.7% **dia** and 59.3% **sod**. With further increase in TC to 100 mg mL^–1^, the fraction of **sod** gradually increases to become the dominant phase. This indicates that concentrated conditions favor the formation of **sod** topology.

The EE% is high up to 95% at precursor concentrations lower than 75 mg mL^–1^, and it only shows a decrease at the highest precursor concentrations (≥75 mg mL^–1^, Figures 2c,2f,2i). For example, in P31, the EE% decreases sharply from 92% at TC = 75 mg mL^–1^ to 34% at TC = 100 mg mL^–1^. Reductions in EE% are observed in P32 and P34 at TC = 75 and 100 mg mL^–1^, respectively.

These samples showcase how, from a subset of 15 samples, three different concentration-driven structural transformation pathways and three different encapsulation trends can be determined by using classical 2D plots. However, as they stand, the observed trends remain isolated within the space of variables, giving information on ∼5% of the entries. While PXRD plots and selected EE% values were previously sequentially analyzed,^26^ in the current study the additional characterizations (IR data, the full sets of EE% and LC%) exceed the capacity for simultaneous manual inspection.

To plot data sets, 3D visualizations can help, but the data can result in complex plots that are difficult to interpret. Simplifications are helpful but can reduce the accessible information. For example, for mixed phases, a visualization threshold was used (e.g., only phases >50% are visualized),^26^ obscuring some phase domains and transitions (Figure 3a). By representing phase boundaries as interpolated curved surfaces, phase domains become easier to visualize, but this useful representation remains a simplification of a complex system and inevitably hides local variability (Figure 3b). Moreover, these plots provided fixed directional views, without the possibility of customizing the exploration of the collected data.

**Figure 3:**
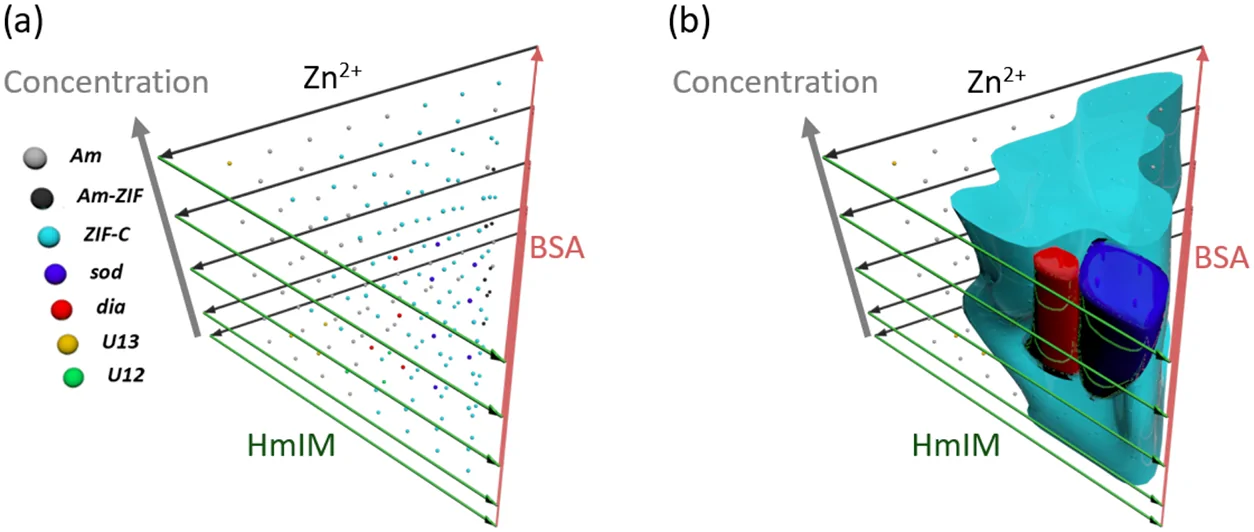
Previously reported plots adapted from Hafner et al.^26^ showing (a) the dominant phases (threshold >50 wt %) in the space of the variables. Information about the coexistence of other phases is lost. The plot is rendered from a fixed directional view. While this 3D data cluster provides a useful generic view, this representation makes the exploration of each individual experiment difficult. (b) Phase domains represented using curved surfaces connecting boundary regions. The static visualization and the threshold-based simplification obscure local variability within phase domains.

These limitations can be addressed through interactive navigation, which enables the direct inspection of neighboring synthesis conditions and their associated experimental outputs. This motivated us to explore the use of a digital platform to design innovative visualizations for the ZIF biocomposites.

### 2.3 ZIF Biocomposite Explorer Application

To enable systematic interpretation of the full data set, synthesis parameters, PXRD plots, IR spectra, and EE% and LC% data were integrated into a digital platform here named ZIF Biocomposite Explorer App (ZIF-BEA). Each experiment is treated as a unique synthesis coordinate defined by its formulation position in the ternary composition space, the TC, and the washing protocol and is linked to the corresponding experimental property descriptors. The organization of this data set encodes a direct correspondence among the programmable pump protocol, the compositional variables, and the resulting structural and functional properties, thereby supporting consistent comparison across the explored parameter space (Figure 1).

PXRD data were first processed via the ZIF Phase Analysis webapp^56^ developed by our group, where the crystalline phase assignment is performed by an automated procedure that includes baseline correction, smoothing, and peak detection, followed by a search for matching reference peaks across the diverse candidate ZIF phases (see Figures S13–S33, Tables S13 and S14, Supporting Information).

To access additional structural details, in ZIF-BEA, the crystalline-to-amorphous material ratio of each sample was estimated by a procedure illustrated in detail in the Supporting Information (Section 1.5). The amorphous component can be ascribed to both the disordered coordination network fraction of the ZIF material and the intrinsically disordered protein confined within the carrier material (Figure 4). We note that in MOF biocomposites, this protein-related contribution to the amorphous fraction is often overlooked, with the amorphous signal commonly attributed only to disorder in the coordination network.

**Figure 4:**
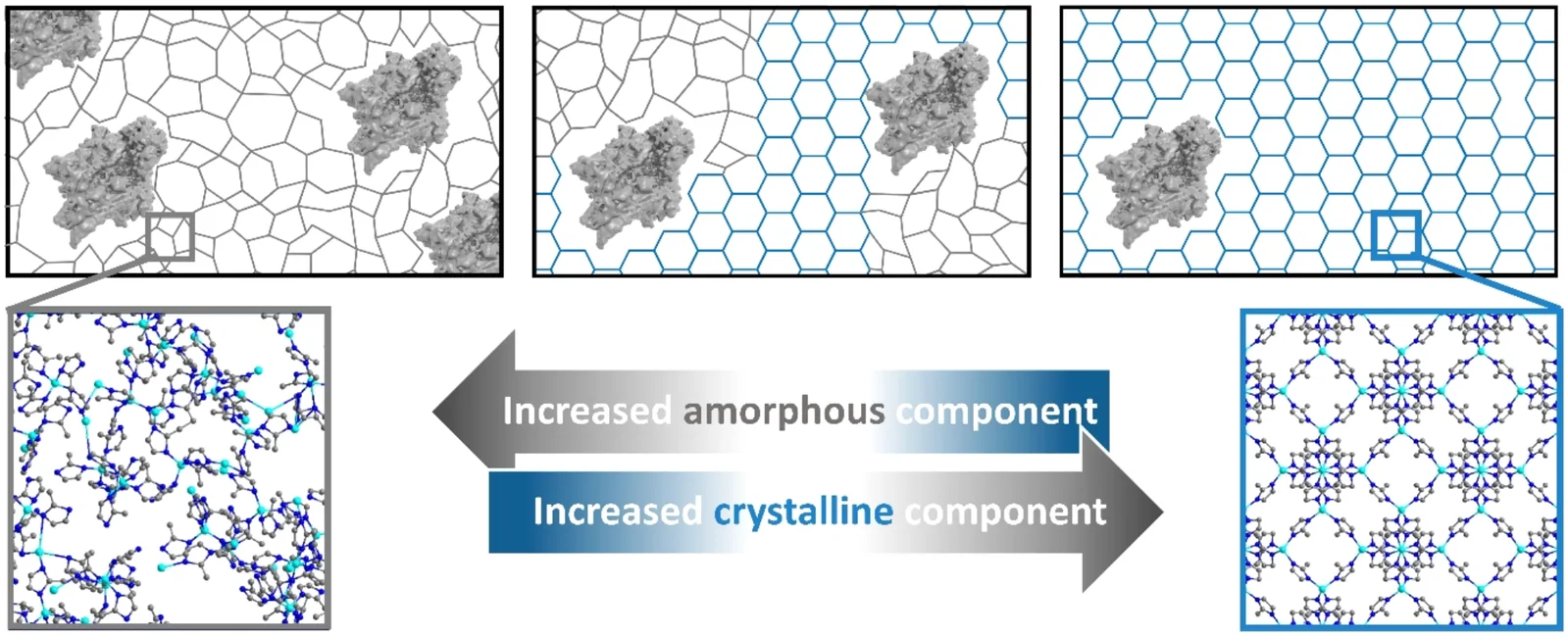
Increasing crystallinity is associated with increasing long-range order of the framework and decreasing protein content. The total amorphous fraction arises from both structural disorder within the framework and the protein content.

Next, IR spectra were integrated into ZIF-BEA to provide information about the chemical composition of the samples. Additionally, from IR data, we calculated the ratio between the relative intensity of the ∼1650 cm^–1^ amide I and the ∼421 cm^–1^ Zn–mIM band, enabling visualization of how the relative protein/framework spectral ratio varies with sample preparation (see Figures S53–S72, Supporting Information). EE% and LC% were also incorporated into ZIF-BEA, allowing encapsulation efficiency and loading capacity to be inspected together with the structural and spectroscopic information within the same navigable data set (see Figures S34–S48, Supporting Information). The final data set is visualized in an interactive stacked ternary format, in which ternary coordinates represent the relative contributions of Zn^2+^, HmIM, and BSA, while the stacked layers represent TCs; washing conditions and additional properties can be selected as independent filters (Figure 5). Rather than compressing the synthesis space into a small number of static views, our interface enables rapid navigation across neighboring conditions, inspection of local trajectories through the variable space, and selection of subsets defined by crystallinity, IR information, EE%, LC%, or a combination thereof. The potential offered by ZIF-BEA can be tested from the app (see Figures S10–S12 and Table S12, Supporting Information), and Figure 5 shows its layout.

**Figure 5:**
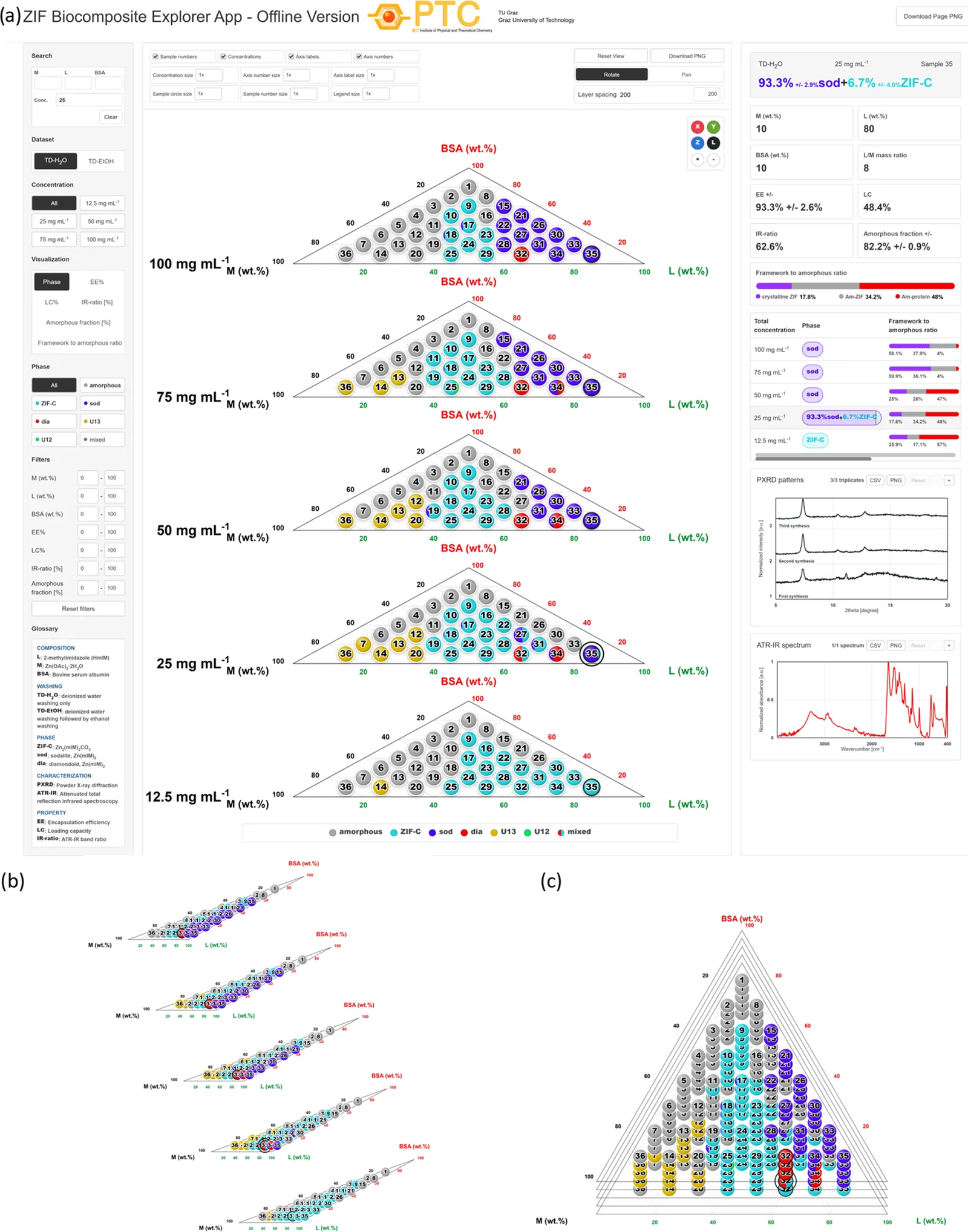
Image downloaded from ZIF-BEA using the built-in Download PNG function for the interactive visualization of the properties of BSA@ZIF biocomposites. ZIF-BEA handles a large data set and displays it as stacked ternary phase maps, where ternary coordinates represent the relative proportions of Zn^2+^, HmIM, and BSA as wt %, and stacked layers correspond to different TCs. The ZIF-BEA interface enables filtering by washing condition, phase, crystallinity, and composition, while linked panels provide sample-specific information, including material composition, IR spectra, PXRD patterns, and experimental conditions. The ternary diagrams in panels (a), (b), and (c) are shown from representative *X*-, *Y*-, and *L*-angle views, respectively. The logo is reproduced with permission from Paolo Falcaro.

#### 2.3.1 Visualization of PXRD-Derived Structural Information in ZIF-BEA

In Figure 6a, the crystalline phases identified from PXRD data and the crystallinity ratio of each TD–H_2_O sample are displayed. For the crystalline phase assignment, we can identify regions where the formation of **sod** is favored (i.e., HmIM > 40 wt % and Zn^2+^ ≤ 20 wt %), where **ZIF-C** is predominant (i.e., 25 wt % < HmIM < 50 wt %) and where amorphous or **U13** materials are formed (i.e., HmIM < 25 wt %). By increasing TC, we observe an increased number of samples with **sod** topology, mainly at the expense of **ZIF-C** samples, in agreement with trends previously observed in manually prepared samples. With the dynamic visualization of ZIF-BEA, here, we move beyond static dominant-phase maps and inspect the data set at the level of individual synthesis coordinates. This enables the direct identification of phase-transition regions and local variability that were previously hidden in threshold-based representations. For example, we can clearly identify regions where, at higher TC, **sod** expands into a larger BSA range, replacing **ZIF-C** and amorphous samples (region highlighted with a dark blue box in Figure 6a). We note that this information could support the design of carriers with a high or low accessible surface area, depending on the target application.

**Figure 6:**
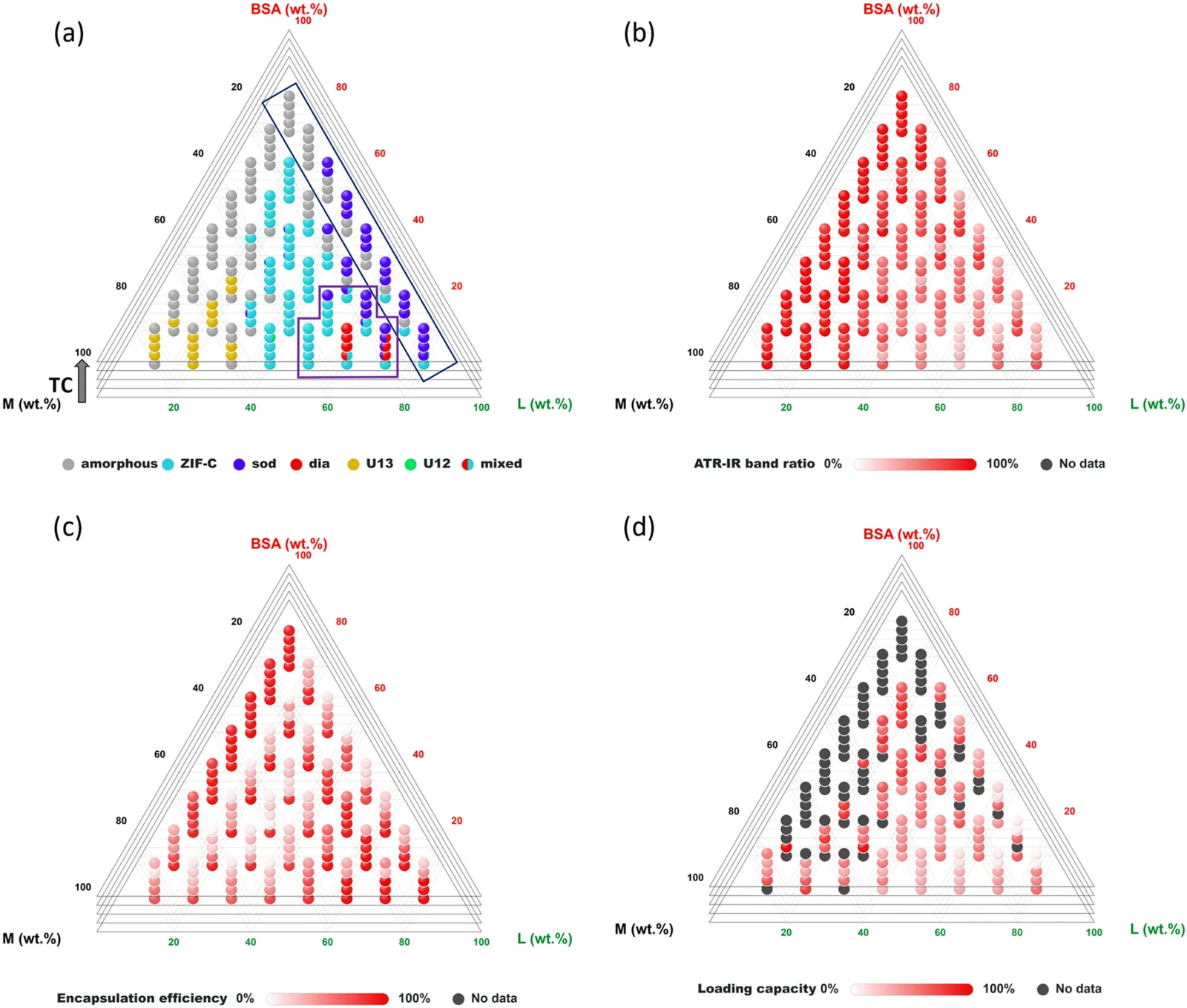
Stacked ternary visualization of TD-H_2_O protein@ZIF biocomposite samples viewed from low (bottom) to high TC (top). The ternary coordinates plot the relative contributions of Zn^2+^, HmIM, and BSA for a specific TC, while the stacked layers correspond to different piled TCs. The inset shows how phase transitions (**ZIF-C** to **dia**, and **ZIF-C** to **dia** to **sod**) across TCs can be visualized. (a) Phase distribution map with two highlighted regions of selected trends discussed in the main text, where light blue, red, and dark blue represent **ZIF-C**, **dia**, and **sod**, respectively; (b) IR data map indicating the ratio between the intensity of the amide I band (∼1650 cm^–1^) and the Zn–N stretching mode (∼421 cm^–1^)—red color for high ratios, white color for low ratios; (c) EE% map—red color for high EE% and white color for low EE%; (d) LC% map—red color for high LC%, white color for low LC%, and gray indicates samples without LC% measurements.

Furthermore, we can inspect local variability, like the region around BSA/HmIM/Zn^2+^ = [10/60/30] (region highlighted with a violet box in Figure 6a). Here, **dia** is obtained for TC > 25 mg mL^–1^, while all the nearest-neighbor samples show different phases and trends over different TC. For example, [10/50/40] yields only **ZIF-C**; [20/60/20] yields **ZIF-C** at TC < 25 mg mL^–1^and **sod** at TC > 50 mg mL^–1^; [10/70/20] undergoes a crystalline phase change from **ZIF-C** to **dia** and finally **sod** while increasing TC. These observations suggest that in this region the ZIF crystallization is highly sensitive to small differences in the BSA/HmIM/Zn^2+^ ratio and TC. As a result, these synthetic conditions might lead to a less consistent material preparation when compared with other more robust regions.

#### 2.3.2 Visualization of IR-Derived Chemical Information in ZIF-BEA

The possibility of performing rapid comparisons within this expanded data set allowed us to explore other functional properties. For example, to obtain qualitative information about the loading of protein in the biocomposites, we systematically analyzed the IR spectra. The IR ratio map in Figure 6b provides complementary spectroscopic information on the relative amounts of BSA and ZIF present in the samples by comparing the relative contributions of the amide I band of BSA and the Zn–N stretching band. A comparison of the IR ratio with the ICP-OES-derived LC% (Figure S50) shows that the general increase of the IR ratio with ICP-OES-derived LC% supports its use as a qualitative indicator of protein loading.

In both the TD–H_2_O (Figure 6b, and Figures S63 to S67, Supporting Information) and TD–EtOH (Figures S68 to S72, Supporting Information) data sets, the IR ratio is lowest in regions with high ligand wt % and increases when lowering ligand content. By comparing the IR ratio map (Figure 6b) with the crystalline phase and crystallinity ratio (Figure 6a), we can observe that the regions where the amorphous samples are predominant (i.e., HmIM < 25 wt %) show the maxima IR ratios, confirming that these samples are mostly composed of aggregated BSA or of Zn-BSA-based material (i.e., **U13**). Conversely, the samples with the lower IR ratio are found in the high HmIM ratio region, and this corresponds to the regions where the crystallinity is higher.

By comparing the TD–H_2_O (Figures 6b, and S63–S67, Supporting Information) and TD–EtOH (Figures S68–S72, Supporting Information) data sets across the different concentration layers, we observe that the TD–H_2_O IR maps have broader regions of high IR ratios. This reflects the larger number of partially or fully amorphous samples in the water-washed set. The IR maps therefore complement the phase and crystallinity ratio assignments by showing how the relative balance between protein-associated and framework-associated infrared modes evolves across the same synthesis coordinates. This information could expedite qualitative IR screening of a large set of samples, thus focusing PXRD or ICP analyses on specific subsets of interest.

#### 2.3.3 Visualization of Protein Immobilization Information in ZIF-BEA

Next, the protein immobilization was quantified by calculating the EE% and the LC%. Across the EE% map, protein immobilization shows a dependence on both the HmIM ratio and the TC, in agreement with previous trends from a small subset of samples from the manual procedure (Figures 6c, S9 and S34–S38, Supporting Information). In general, the lowest TC showed the highest number of samples with high EE%>80% (i.e., ∼80% of samples), distributed within two regions (i.e., samples with HmIM <20 wt % and samples with HmIM >50 wt %), while increasing TC leads to a progressive reduction in EE% across most of the synthesis space (e.g., ∼11% of samples with EE%>80% for the maximum TC), with only regions with HmIM <20 wt % maintaining high EE%.

Then, LC% was measured for the samples that showed the presence of crystalline ZIFs, since fully amorphous samples and Zn-BSA-based materials (i.e., **U13**) are currently of minor interest for biotechnological applications.^26^ LC% shows a clear dependence on both the precursor ratio and TC (Figures 6d, S39–S48, Supporting Information). The LC% increases with sample amorphization (i.e., BSA > 50 wt %). Unlike EE%, the LC% tends to be invariant for the same sample among different TC values across most of the synthesis space. Only in the region where there is a high excess of ligand (more than 60 wt %), LC% tends to decrease at higher TC values, similar to the EE% trend: this suggests that, at high TC and high ligand ratios, homogeneous ZIF nucleation is favored, and the presence of BSA as a heterogeneous nucleating agent is less influential. We also note that no direct correlation was observed between LC% and EE% (Figure S51). This reflects the fact that EE% quantifies the fraction of the initially added BSA that is retained in the recovered biocomposite and is therefore independent of the amount of ZIF powder produced. In contrast, LC% represents the mass fraction of BSA within the recovered biocomposite and thus depends not only on the amount of BSA encapsulated but also on the amount of ZIF formed during the synthesis.

Overall, the observed trends confirm that protein encapsulation in protein@ZIF is governed by a coupled effect of composition and concentration, where higher ligand content favors efficient protein immobilization, while increasing TC tends to reduce the EE%.

It is worth noting that, in our previous work based on the static representation of the data, EE% and LC% were deliberately measured only for a small fraction of samples (∼17%) to represent data with classical 3D plots.^26^ Here, the dynamic visualization of the data in ZIF-BEA enables access to all EE% and LC% trends throughout the entire space of variables.

#### 2.3.4 Amorphous Content Nature and Trends from ZIF-BEA

Next, to investigate the nature of the amorphous fraction, we directly compared the amorphous content estimated from PXRD with the protein content (LC%) determined by ICP-OES. We note that LC% is calculated from the sulfur content measured by ICP-OES. Since the protein is the only source of sulfur in the biocomposite, LC% provides a quantitative measure of the protein content, i.e., LC% = protein mass/total biocomposite mass. The remaining mass of the biocomposite consists of ZIF, which may be present in either crystalline or amorphous form (Figure 4). However, LC% alone does not distinguish between these two contributions. In contrast, PXRD enables a semiquantitative estimation of the relative proportions of crystalline and amorphous material.^25^ Therefore, comparing the PXRD-derived amorphous fraction with the LC% can indicate whether the amorphous component arises from the protein alone or whether it may also include an amorphous ZIF fraction. We hypothesized that if the amorphous fraction of the biocomposite were entirely attributable to the protein content, the amorphous fraction derived from PXRD would match LC%. Conversely, if an amorphous ZIF component were present, the PXRD-derived amorphous fraction would be higher than the LC%. To investigate these hypotheses, we plotted the amorphous fraction calculated from PXRD patterns against the LC% obtained from ICP-OES (see Figure 7a). Figures S49–S52 show the relationship between amorphous fraction, IR ratio, EE%, and LC% (Supporting Information). For the TD-H_2_O samples, the PXRD-derived amorphous fraction is generally higher than the LC%. This suggests that the amorphous material cannot be attributed to the protein content alone, indicating the presence of a significant amorphous ZIF fraction. In contrast, for the TD-EtOH samples, good agreement between the two quantification methods is observed for samples with LC% >20%. Based on this qualitative analysis, we conclude that the amorphous fraction in the TD-H_2_O samples consists of both protein and amorphous ZIF, while the amorphous fraction in the TD-EtOH samples consists primarily of protein. Upon ethanol washing, the amorphous ZIF can be converted to crystalline ZIF (i.e., **sod**),^26^ such that the protein becomes the primary contributor to the amorphous fraction of the material, leading to agreement between the PXRD-derived amorphous fraction and the protein fraction of the biocomposite (i.e., LC%). We note that, for the small subset of samples with LC% < 20% (obtained at TC ≥50 mg mL^–1^; 18 samples in total), the PXRD-derived amorphous fraction tends to be higher than the LC% in both TD-H_2_O and TD-EtOH samples. This observation suggests that the PXRD-based amorphous quantification may be less accurate at low LC% values because this approach relies on the simplifying assumption that the crystalline and amorphous fractions possess comparable X-ray scattering factors, such that the integrated diffraction intensity is proportional to their relative mass fractions. In addition, a fraction of amorphous ZIF may not be fully converted to crystalline ZIF during ethanol washing in these samples. Thus, by combining the PXRD-derived amorphous fraction with LC%, we can distinguish between the amorphous ZIF and protein contributions to the amorphous component of the biocomposite (Figure 4) and ultimately estimate the relative contents of crystalline ZIF, amorphous ZIF, and protein in the crystalline samples. The results are displayed in Figure 7b and can be explored with the offline ZIF-BEA. Different trends can be identified. For example, we notice that the samples with a higher relative crystalline ZIF content are more frequently obtained at high TC and high ligand wt % (>50 mg mL^–1^, >60 wt %), and that the weight fraction of amorphous ZIF in the total amorphous content of the biocomposite is higher for lower BSA wt % (<60 wt %). This information could support the design of carriers with different degrees of crystallinity and protein content, properties that can influence both the cargo release profile and the biocomposite cytotoxicity.^26^ For example, a high fraction of amorphous ZIF might be desirable to maximize the release rate, while a biocomposite composed only of protein and crystalline ZIF might be desirable to minimize the release rate.

**Figure 7:**
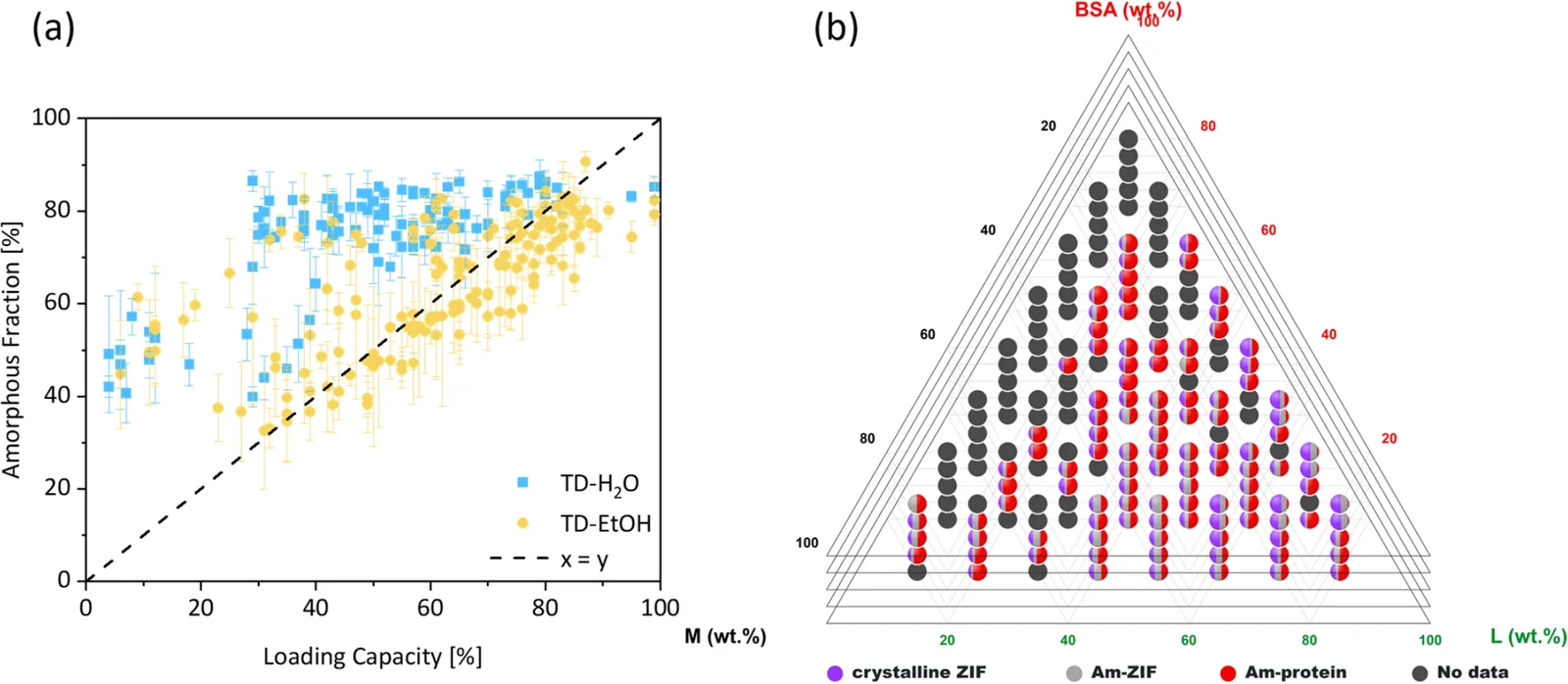
(a) Amorphous fraction plotted against LC% for TD-H_2_O and TD-EtOH samples; the dashed line indicates *x* = *y*. (b) TD-H_2_O samples showing the relative fractions of crystalline ZIF (violet), amorphous ZIF (Am-ZIF, gray), and amorphous protein (Am-protein, red), highlighting the dual nature of the amorphous fraction.

#### 2.3.5 Filtering Structural, Chemical, and Functional Properties in ZIF-BEA

An additional advantage of this interactive platform is the ability to dynamically filter the data set based on user-defined criteria (Figure 8). Input fields allow the selection of samples according to structural, functional, and chemical descriptors, such as crystalline phase, EE %, LC %, and IR ratios. This functionality enables rapid identification and highlighting of regions of interest within the TDs, facilitating the targeted exploration of the synthesis space. In contrast to static representations where such multiparameter filtering is not possible, the proposed approach here allows users to isolate specific material profiles and directly relate them to neighboring synthesis conditions and experimental outputs. For example, it is possible to selectively display a 2D slice of the data set (e.g., samples prepared from Zn^2+^ = 20 wt %, Figure 8a). Then, samples with a high amorphous content (>80%, Figure 8b) can be displayed, discriminating between those fully amorphous and those with a crystalline ZIF component. Next, it is possible to add a third filter to visualize high EE% values (>80%, Figure 8c) and analyze their LC% (Figure 8d). This discretization of the target values allows users to identify the ZIF biocomposite formulation with the selected chemical, structural, and functional properties.

**Figure 8:**
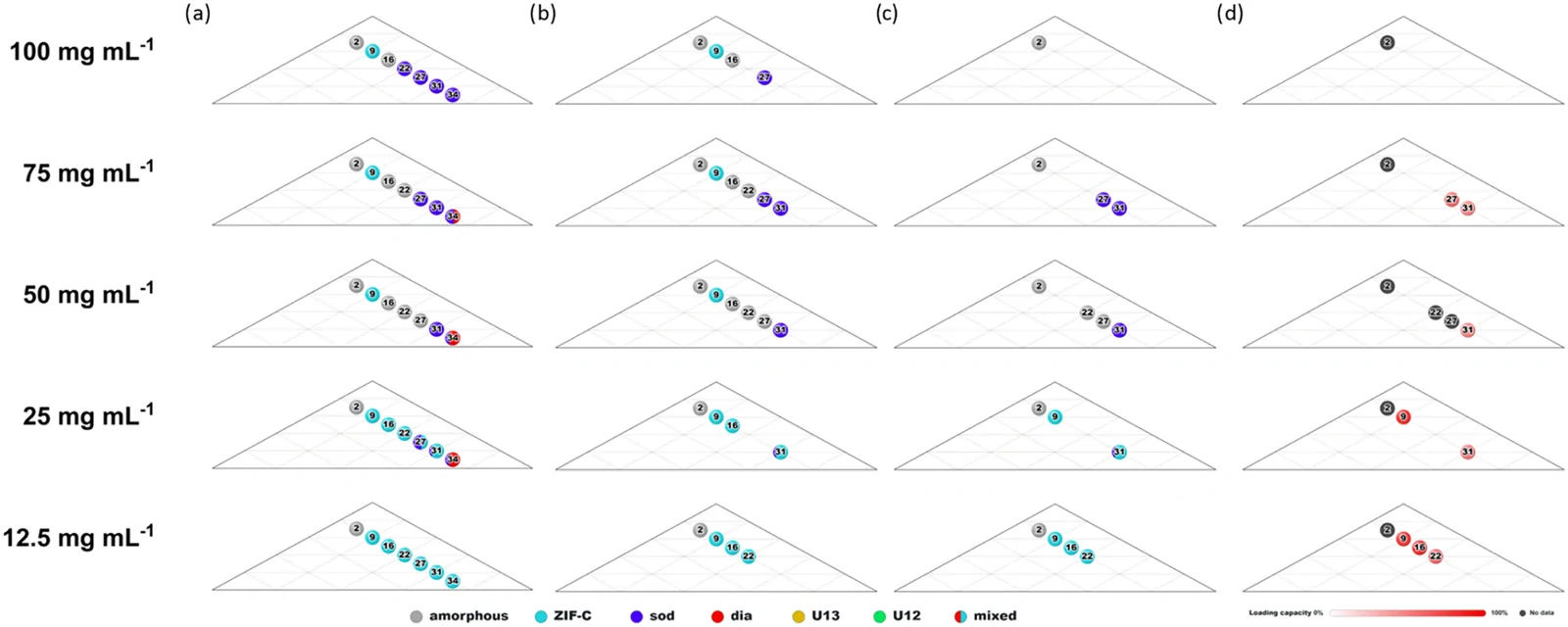
Interactive filtering and visualization of protein@ZIF biocomposite samples prepared using the water washing procedure in ZIF-BEA from low (bottom) to high total concentration (top). (a) 2D slice of the data set selected at Zn^2+^ = 20 wt %, showing the distribution of samples across stacked ternary diagrams representing different total concentrations. (b) Filtered visualization of samples with amorphous fraction higher than 80%. (c) Further filtering of the same region to highlight samples with high encapsulation efficiency (EE% > 80%). (d) Loading capacity (LC%) map of the filtered samples, where red indicates high LC%, white indicates low LC%, and gray indicates samples without LC% measurements.

In addition to the evaluation of the collected data set, ZIF-BEA is designed as a practical tool for researchers interested in a direct comparison between synthesis conditions in a given laboratory and ZIF-BEA data. Specifically, users can input their own synthesis parameters, namely, metal, ligand, and biomacromolecule content, and total precursor concentration. These inputs are displayed directly onto the existing data set, enabling users to visualize where their experimental conditions lie within the explored synthesis space. This feature facilitates a straightforward comparison with neighboring samples and provides practical guidance. For example, users can identify directions to adjust synthesis parameters and maximize LC% and/or crystallinity depending on the targeted application.

By enabling user-driven exploration, ZIF-BEA establishes an original tool for exploring synthesis–structure–property relationships, moving beyond static maps toward interactive data analysis.

## 3 Conclusion

We applied a semiautomated preparation method for the synthesis of ZIF biocomposites, in which syringe pumps and stirring minimize the variability introduced by classical manual procedures and thereby enhance consistency within the same set of samples. Using this approach, the variable space was systematically explored through 360 synthesis conditions by varying the weight ratios among metal, ligand, protein, and the total precursor concentration. Compared with previous studies, this work systematically collects additional structural, chemical, and functional descriptors, including crystalline-to-amorphous ratios, IR data providing a qualitative indication of the protein-to-ZIF contributions, and encapsulation efficiency values for all samples, while loading capacity (LC%) was measured for samples with a significant crystalline fraction (266 samples). To make this large data set directly accessible, we built an interactive platform, ZIF-BEA, which enables interrogation of ∼2250 entries through rapid sequential inspection of synthesis coordinates and allows detailed evaluation of property trends, local anomalies, and replicate variability across samples. Through this interactive visualization, the different behaviors of EE% and LC% and the contribution of increasing protein content to the overall amorphous fraction of the biocomposite become more readily visible. The different trends in EE% and LC% can be explained by variations in the amount of ZIF formed under different synthesis conditions. Furthermore, free access to ZIF-BEA supports interrogation of the database: by entering specific compositional coordinates and by evaluating the surrounding experiments, users can qualitatively estimate the salient properties of a hypothetical biocomposite and identify synthetic directions to enhance desired properties, such as crystallinity or the proportion between protein and MOF components. Overall, this interactive platform provides customized access to a large data set, facilitates the design of ZIF biocomposites with tailored properties, and suggests that similar dynamic exploration of complex data sets can expedite material engineering in other classes of functional materials.

## 4 Experimental Section

### 4.1 General Remarks

Zinc acetate dihydrate (Zn­(OAc)_2_·2H_2_O, ACS grade, Merck), 2-methylimidazole (HmIM, 99%, Sigma-Aldrich), bovine serum albumin (BSA, lyophilized powder, ≥96%, Sigma-Aldrich), Bradford reagent (for 0–1.4 mg mL^–1^ protein, Sigma-Aldrich), and ethanol (99%, Roth) were purchased from commercial suppliers and used as received without further purification. Deionized (DI) water was used in all experiments.

### 4.2 Semiautomated Synthesis of ZIF Biocomposites

A systematic synthesis matrix was designed to explore the predefined variable space with high repeatability. Each sample was assigned a compact identifier

[C−W−P−R]

where *C* denotes the total precursor concentration level (*C*_1_–*C*_5_: 12.5, 25, 50, 75, and 100 mg mL^–1^), *W* is the washing regime (“WW” for DI water washing, “EW” for DI water followed by ethanol washing), *P* denotes the composition coordinate (1–36), and *R* is the replicate index (Round 1–3).

Based on the design, the experimental matrix comprised 5 total concentrations (TCs), 36 composition coordinates, and 2 washing regimes, resulting in a total of 5 × 36 × 2 = 360 unique conditions. Each condition was prepared in triplicate (*n* = 3) under identical automated control scripts, yielding 360 × 3 = 1080 individual samples.

All syntheses were executed using a software-controlled syringe-pump platform.^55^ Each run was defined by a protocol file encoding: (i) reagent identities and stock concentrations, (ii) dosing order, (iii) flow rates, (iv) interstep delays, (v) aspiration and dispensing cycles, and (vi) total reaction volume. The reaction volume was fixed at 2.0 mL for all matrix points, ensuring that variations in the total precursor concentration corresponded to controlled dilution.

### 4.3 Washing Procedures

Depending on the assigned ternary-diagram (TD) group, the washing process was performed using either the TD-H_2_O (WW) procedure or the TD-EtOH (EW) procedure:

#### 4.3.1 TD-H_2_O

The solid product was separated via centrifugation (13,000 rpm for 5 min), and the reaction supernatant was retained for subsequent determination of EE%. The powder was resuspended in 2.0 mL DI water using a vortex mixer (3000 rpm for 1 min). The suspension was centrifuged (13,000 rpm for 5 min) to yield a pellet, and the supernatant was discarded. This procedure was repeated 3 times (a total of 4 DI-washing steps including the initial separation). Finally, the powder was air-dried for 48 h at room temperature (23 °C).^26^

#### 4.3.2 TD-EtOH

The solid product was separated via centrifugation (13,000 rpm for 5 min), and the reaction supernatant was retained for subsequent determination of EE%. The powder was resuspended in 2.0 mL DI water using a vortex mixer (3000 rpm for 1 min) and then centrifuged (13,000 rpm for 5 min), and the supernatant was discarded. This water washing was repeated twice (3 DI-water washing steps in total, including the initial separation). Afterward, the pellet was resuspended in 2.0 mL ethanol (EtOH) using a vortex mixer (3000 rpm for 1 min) and centrifuged (13,000 rpm for 5 min), and the supernatant was discarded. This ethanol washing was repeated once (2 EtOH washing steps in total). Finally, the powder was air-dried for 48 h at room temperature (23 °C).^26^

### 4.4 Characterization

Powder X-ray diffraction (PXRD) patterns were collected at room temperature using a Rigaku SmartLab X-ray Diffractometer (Cu Kα radiation, λ = 1.5406 Å) with a step size of 0.01°, a scanning range of 5–20°, and a scanning rate of 6.0° min^–1^.

Infrared (IR) measurements were carried out at room temperature using a Bruker α spectrometer in Attenuated Total Reflectance (ATR) mode with a diamond window, with a resolution of 2 cm^–1^, 32 acquisitions, and a spectral range of 4000–400 cm^–1^.

Spectra were used as compositional checks to confirm: (i) framework formation (e.g., Zn–N bands characteristic of imidazolate coordination) and (ii) guest immobilization (Amide I and Amide II bands for proteins, where relevant). Where needed, spectra were baseline-corrected and normalized to enable qualitative comparison across the mapped synthesis space.

## Supplementary Material





## Data Availability

The software to control the syringe pump was specifically developed for the current study and can be downloaded from Zenodo (https://zenodo.org/records/20268990). The experimental data can be downloaded from Zenodo (https://zenodo.org/records/20330906). ZIF-BEA can be accessed offline (available in the Supporting Information as downloadable Offline_ZIF-BEA.zip).

## Notes

Offline version of ZIF-BEA (ZIP)
Experimental details (i.e., materials, synthesis, analytical procedures, data set generation), the plots of all the PXRD and IR data and of the relationship between amorphous fraction and LC%, tables with detailed synthesis conditions, tables with PXRD data interpretation, tables with EE% and LC% data, TDs displaying crystalline phase assignment, calculated IR ratios, EE%, and LC%, guidelines for the use of the offline ZIF-BEA, data related to the comparison of semiautomated and manual workflows, data related to the comparison of synthesis with and without stirring (PDF)

## References

[1] Furukawa H., Cordova K. E., O’Keeffe M., Yaghi O. M. (2013). The Chemistry and
Applications of Metal–Organic Frameworks. Science.

[2] Liang K., Ricco R., Doherty C. M., Styles M. J., Bell S., Kirby N., Mudie S., Haylock D., Hill A. J., Doonan C. J., Falcaro P. (2015). Biomimetic Mineralization of Metal–Organic
Frameworks as Protective Coatings for Biomacromolecules. Nat. Commun..

[3] Astria E., Thonhofer M., Ricco R., Liang W., Chemelli A., Tarzia A., Alt K., Hagemeyer C. E., Rattenberger J., Schroettner H. (2019). Carbohydrates@MOFs. Mater. Horiz..

[4] Poddar A., Conesa J. J., Liang K., Dhakal S., Reineck P., Bryant G., Pereiro E., Ricco R., Amenitsch H., Doonan C. (2019). Encapsulation, Visualization and Expression of Genes
with Biomimetically Mineralized Zeolitic Imidazolate Framework-8 (ZIF-8). Small.

[5] Alsaiari S. K., Patil S., Alyami M., Alamoudi K. O., Aleisa F. A., Merzaban J. S., Li M., Khashab N. M. (2018). Endosomal Escape
and Delivery of CRISPR/Cas9 Genome Editing Machinery Enabled by Nanoscale
Zeolitic Imidazolate Framework. J. Am. Chem.
Soc..

[6] Li S., Dharmarwardana M., Welch R. P., Benjamin C. E., Shamir A. M., Nielsen S. O., Gassensmith J. J. (2018). Investigation of Controlled Growth
of Metal–Organic Frameworks on Anisotropic Virus Particles. ACS Appl. Mater. Interfaces.

[7] Gan L., Velásquez-Hernández M. D. J., Emmerstorfer-Augustin A., Wied P., Wolinski H., Dal Zilio S., Solomon M., Liang W., Doonan C., Falcaro P. (2022). Multi-Layered
ZIF-Coated Cells for the Release of Bioactive Molecules in Hostile
Environments. Chem. Commun..

[8] Yu Z., Lepoitevin M., Serre C. (2025). Iron-MOFs for Biomedical Applications. Adv. Healthcare Mater..

[9] Abánades
Lázaro I., Chen X., Ding M., Eskandari A., Fairen-Jimenez D., Giménez-Marqués M., Gref R., Lin W., Luo T., Forgan R. S. (2024). Metal–Organic
Frameworks for Biological Applications. Nat.
Rev. Methods Primers.

[10] Abánades
Lázaro I., Vicent-Morales M., Mínguez Espallargas G., Giménez-Marqués M. (2023). Hierarchical Mesoporous NanoMUV-2
for the Selective Delivery of Macromolecular Drugs. J. Mater. Chem. B.

[11] Liang W., Wied P., Carraro F., Sumby C. J., Nidetzky B., Tsung C.-K., Falcaro P., Doonan C. J. (2021). Metal–Organic
Framework-Based Enzyme Biocomposites. Chem.
Rev..

[12] Cao Y., Li X., Ge J. (2021). Enzyme Catalyst Engineering toward
the Integration
of Biocatalysis and Chemocatalysis. Trends Biotechnol..

[13] Liang W., Zheng S., Shu Y., Huang J. (2024). Machine Learning Optimizing
Enzyme/ZIF Biocomposites for Enhanced Encapsulation Efficiency and
Bioactivity. JACS Au.

[14] Liang W., Flint K., Yao Y., Wu J., Wang L., Doonan C., Huang J. (2023). Enhanced Bioactivity
of Enzyme/MOF
Biocomposite via Host Framework Engineering. J. Am. Chem. Soc..

[15] Glatz J., Cases Díaz J., Salinas-Uber J., Talens-Perales D., Polaina J., Giménez-Marqués M. (2025). Channel-Directed
Enzymatic Depolymerization within a Metal–Organic Framework. ACS Appl. Mater. Interfaces.

[16] Wijesundara Y. H., Howlett T. S., Kumari S., Gassensmith J. J. (2024). The Promise
and Potential of Metal–Organic Frameworks and Covalent Organic
Frameworks in Vaccine Nanotechnology. Chem.
Rev..

[17] Horcajada P., Gref R., Baati T., Allan P. K., Maurin G., Couvreur P., Férey G., Morris R. E., Serre C. (2012). Metal–Organic
Frameworks in Biomedicine. Chem. Rev..

[18] McKinlay A.
C., Morris R. E., Horcajada P., Férey G., Gref R., Couvreur P., Serre C. (2010). BioMOFs: Metal–Organic
Frameworks for Biological and Medical Applications. Angew. Chem., Int. Ed..

[19] Yu P., Zheng X., Alimi L. O., Al-Babili S., Khashab N. M. (2024). Metal–Organic Framework-Mediated Delivery of
Nucleic Acid across Intact Plant Cells. ACS
Appl. Mater. Interfaces.

[20] Liu X., Fatieiev Y., Khashab N. M. (2025). Supramolecular
Porous Materials for
Biomedical Applications. Adv. Healthcare Mater..

[21] Velásquez-Hernández M. d. J., Linares-Moreau M., Astria E., Carraro F., Alyami M. Z., Khashab N. M., Sumby C. J., Doonan C. J., Falcaro P. (2021). Towards Applications of Bioentities@MOFs in Biomedicine. Coord. Chem. Rev..

[22] Shieh F.-K., Wang S.-C., Yen C.-I., Wu C.-C., Dutta S., Chou L.-Y., Morabito J. V., Hu P., Hsu M.-H., Wu K. C.-W., Tsung C.-K. (2015). Imparting Functionality
to Biocatalysts
via Embedding Enzymes into Nanoporous Materials by a de Novo Approach:
Size-Selective Sheltering of Catalase in Metal–Organic Framework
Microcrystals. J. Am. Chem. Soc..

[23] Vo T. H., Lin S.-W., Lin M.-C., Kuan P.-Y., Chen J.-H., Huang H.-K., Liu W.-T., Xu H., Li A., Hsu Y.-A. (2025). Exploring Enzyme Encapsulation
Efficiency in MOFs Using
Eco-Friendly Approaches. ChemSusChem.

[24] Carraro F., Velásquez-Hernández M. D. J., Astria E., Liang W., Twight L., Parise C., Ge M., Huang Z., Ricco R., Zou X. (2020). Phase
Dependent Encapsulation
and Release Profile of ZIF-Based Biocomposites. Chem. Sci..

[25] Hermans P. H., Weidinger A. (1948). Quantitative X-Ray Investigations on the Crystallinity
of Cellulose Fibers. A Background Analysis. J. Appl. Phys..

[26] Hafner M. R., Carraro F., Abbasgholi-NA B., Dal Zilio S., Schmallegger M., Wiltsche H., Ge L., Reithofer M. R., Astria E., Villanova L. (2026). 4D Mapping of ZIF Biocomposites
for High Protein Loading and Tunable Release Profiles. Adv. Funct. Mater..

[27] Feng Y., Cao X., Zhang L., Li J., Cui S., Bai Y., Chen K., Ge J. (2022). Defect Engineering
of Enzyme-Embedded
Metal–Organic Frameworks for Smart Cargo Release. Chem. Eng. J..

[28] Hu C., Bai Y., Hou M., Wang Y., Wang L., Cao X., Chan C.-W., Sun H., Li W., Ge J., Ren K. (2020). Defect-Induced Activity Enhancement of Enzyme-Encapsulated Metal–Organic
Frameworks Revealed in Microfluidic Gradient Mixing Synthesis. Sci. Adv..

[29] Borrego-Marin E., Garrido-Barros P., Peterson G. W., Vismara R., Carmona F. J., Barea E., Navarro J. A. R. (2025). Reactive ZIF-L Crystal Surface for
Organophosphorous Degradation and Acetylcholinesterase Reactivation. J. Am. Chem. Soc..

[30] Basnayake S. A., Su J., Zou X., Balkus K. J. (2015). Carbonate-Based
Zeolitic Imidazolate Framework for Highly Selective CO2 Capture. Inorg. Chem..

[31] Ge M., Wang Y., Carraro F., Liang W., Roostaeinia M., Siahrostami S., Proserpio D. M., Doonan C., Falcaro P., Zheng H., Zou X., Huang Z. (2021). High-Throughput Electron
Diffraction Reveals a Hidden Novel Metal–Organic Framework
for Electrocatalysis. Angew. Chem., Int. Ed..

[32] Katsenis A. D., Puškarić A., Štrukil V., Mottillo C., Julien P. A., Užarević K., Pham M.-H., Do T.-O., Kimber S. A. J., Lazić P. (2015). In Situ X-ray Diffraction Monitoring of a Mechanochemical Reaction
Reveals a Unique Topology Metal–Organic Framework. Nat. Commun..

[33] Low Z. X., Yao J., Liu Q., He M., Wang Z., Suresh A. K., Bellare J., Wang H. (2014). Crystal Transformation in Zeolitic-Imidazolate
Framework. Cryst. Growth Des..

[34] Carraro F., Williams J. D., Linares-Moreau M., Parise C., Liang W., Amenitsch H., Doonan C. J., Kappe C. O., Falcaro P. (2020). Continuous-Flow
Synthesis of ZIF-8 Biocomposites with Tunable Particle Size. Angew. Chem., Int. Ed..

[35] Ogata A. F., Rakowski A. M., Carpenter B. P., Fishman D. A., Merham J. G., Hurst P. J., Patterson J. P. (2020). Direct
Observation of Amorphous Precursor
Phases in the Nucleation of Protein–Metal–Organic Frameworks. J. Am. Chem. Soc..

[36] Rose B., Olivas E. M., Prasad P. R., Li Z., Wang F., Sroge C., Talosig A. R., Carpenter B. P., Martin R. W., Fowler W. C., Patterson J. P. (2026). The Role
of Carbon Sources in the Formation of Carbonate-Based Zeolitic Imidazolate
Framework Protein Biocomposites. Chem. Mater..

[37] Talosig A. R., Wang F., Mulvey J. T., Carpenter B. P., Olivas E. M., Katz B. B., Zhu C., Patterson J. P. (2024). Understanding
the Nucleation and Growth of ZIF-8 Polymorphs. Cryst. Growth Des..

[38] Maddigan N. K., Tarzia A., Huang D. M., Sumby C. J., Bell S. G., Falcaro P., Doonan C. J. (2018). Protein
Surface Functionalisation
as a General Strategy for Facilitating Biomimetic Mineralisation of
ZIF-8. Chem. Sci..

[39] Liang W., Xu H., Carraro F., Maddigan N. K., Li Q., Bell S. G., Huang D. M., Tarzia A., Solomon M. B., Amenitsch H. (2019). Enhanced Activity of Enzymes Encapsulated in Hydrophilic Metal–Organic
Frameworks. J. Am. Chem. Soc..

[40] Liang W., Ricco R., Maddigan N. K., Dickinson R. P., Xu H., Li Q., Sumby C. J., Bell S. G., Falcaro P., Doonan C. J. (2018). Control of Structure Topology and Spatial Distribution
of Biomacromolecules in Protein@ZIF-8 Biocomposites. Chem. Mater..

[41] Li Y.-M., Yuan J., Ren H., Ji C.-Y., Tao Y., Wu Y., Chou L.-Y., Zhang Y.-B., Cheng L. (2021). Fine-Tuning the Micro-Environment
to Optimize the Catalytic Activity of Enzymes Immobilized in Multivariate
Metal–Organic Frameworks. J. Am. Chem.
Soc..

[42] Patterson J. P., Abellan P., Denny M. S., Park C., Browning N. D., Cohen S. M., Evans J. E., Gianneschi N. C. (2015). Observing
the Growth of Metal–Organic Frameworks by in Situ Liquid Cell
Transmission Electron Microscopy. J. Am. Chem.
Soc..

[43] Carpenter B. P., Rose B., Olivas E. M., Navarro M. X., Talosig A. R., Hurst P. J., Di Palma G., Xing L., Guha R., Copp S. M., Patterson J. P. (2024). The Role of Protein Folding in Prenucleation
Clusters on the Activity of Enzyme@Metal–Organic Frameworks. J. Mater. Chem. A.

[44] Wu M., Du Y., Xu H., Zhang X., Ma J., Li A., Chou L.-Y. (2025). Enzyme
Surface Residues Direct Encapsulation into Metal–Organic
Frameworks for Performance Regulation. Angew.
Chem., Int. Ed..

[45] Carpenter B. P., Talosig A. R., Rose B., Di Palma G., Patterson J. P. (2023). Understanding
and Controlling the Nucleation and Growth of Metal–Organic
Frameworks. Chem. Soc. Rev..

[46] Anderson D. G., Lynn D. M., Langer R. (2003). Semi-Automated
Synthesis and Screening
of a Large Library of Degradable Cationic Polymers for Gene Delivery. Angew. Chem., Int. Ed..

[47] Enciso A. E., Abid Z. M., Simanek E. E. (2014). Rapid,
Semi-Automated Convergent
Synthesis of Low Generation Triazine Dendrimers Using Microwave Assisted
Reactions. Polym. Chem..

[48] Monterrubio I., Orive J., Ismail M., Castillo E., García J., Redondo I., Dauvergne J.-L., Saracibar A., Carrasco J., Casas-Cabanas M., Reynaud M. (2025). Affordable Automated
Modules for Lab-Scale High-Throughput Synthesis of Inorganic Materials. Chem. - Eur. J..

[49] Xie Y., Zhang C., Deng H., Zheng B., Su J.-W., Shutt K., Lin J. (2021). Accelerate Synthesis of Metal–Organic
Frameworks by a Robotic Platform and Bayesian Optimization. ACS Appl. Mater. Interfaces.

[50] Goswami S., Rimoldi M., Anderson R., Lee C., Li X., Li A., Deria P., Chen L. X., Schaller R. D., Gómez-Gualdrón D. A. (2022). Toward Ideal Metal–Organic Framework Thin-Film Growth via
Automated Layer-by-Layer Deposition: Examples Based on Perylene Diimide
Linkers. Chem. Mater..

[51] Pilz L., Natzeck C., Wohlgemuth J., Scheuermann N., Weidler P. G., Wagner I., Wöll C., Tsotsalas M. (2023). Fully Automated Optimization of Robot-Based MOF Thin
Film Growth via Machine Learning Approaches. Adv. Mater. Interfaces.

[52] Avci M. B., Kocer F., Kurul F., Cetin A. E. (2025). An Integrated Platform
for Liquid Handling and Cell Imaging in Life Science Applications. Nat. Commun..

[53] Wu Y., Chen Y., Cheng Y. (2024). Building an Arduino-Based Open-Source
Programmable Multichannel Syringe Pump: A Useful Tool for Fluid Delivery
in Microfluidics and Flow Chemistry. J. Chem.
Educ..

[54] Lu J.-M., Pan J.-Z., Mo Y.-M., Fang Q. (2024). Automated
Intelligent
Platforms for High-Throughput Chemical Synthesis. Artif. Intell. Chem..

[55] Kondinski, A. Automated Syringe Pump Dashboard for Multi-Reagent Dispensing in ZIF Biocomposite Synthesis, v1.0.0; Software, Zenodo 2025 10.5281/zenodo.20268990.

[56] Hafner M. R., Villanova L., Carraro F. (2022). App-Based Quantification of Crystal
Phases and Amorphous Content in ZIF Biocomposites. CrystEngComm.

