# Supplementary material for: Semiautomated Synthesis and Interactive Visualization of Zeolitic Imidazolate Framework-Based Biocomposites
Source: Chem Mater. 2026 Sep 29;38(19):10135–48. doi: 10.1021/acs.chemmater.6c01702 (PMC13673939; doi:10.1021/acs.chemmater.6c01702)
Supplement: Supplementary file 1 [file cm6c01702_si_002.pdf]

Semi-automated Synthesis and Interactive Visualization of Zeolitic Imidazolate Framework-Based Biocomposites

Xue Chen[a], Aleksandar Kondinski[a], Jing Yang[a], Helmar Wiltsche[b], Bernd Nidetzky[c], Francesco Carraro[a,], Paolo Falcaro[a,]**

a) Institute of Physical and Theoretical Chemistry, Graz University of Technology, Stremayrgasse 9, Graz, 8010 Austria

b) Institute of Analytical Chemistry and Food Chemistry, Graz University of Technology, Stremayrgasse 9, Graz, 8010 Austria

c) Institute of Biotechnology and Biochemical Engineering, Graz University of Technology, Petersgasse 12/1, Graz, 8010 Austria

Contents

1 Experimental details 3
1.1 General remarks 3
1.2 Semi-automated synthesis of ZIF Biocomposites 3
1.2.1 Synthesis matrix and protocol encoding 3
1.2.2 Dosing sequence and reaction conditions 4
1.2.3 Composition map of synthesis coordinates 5
1.2.4 Concentration-specific dosing tables 7
1.3 Washing procedures 15
1.4 Characterization 16
1.5 PXRD-based amorphous fraction estimation 16
1.6 ATR-IR band-ratio analysis (IR ratio) 18
1.7 EE% 19
1.8 ICP-OES Analysis 20
1.9 Loading capacity (LC%) 20
1.10 Comparison of synthesis with and without stirring 21
1.11 Comparison of semi-automated and manual workflows 27
2 The offline version of ZIF Biocomposite Explorer App 32
2.1 User guidelines for the offline version of ZIF Biocomposite Explorer App 32
2.1.1 Getting started 32
2.1.2 Interface overview 32
2.1.3 Data Exploration and Visualization 33
2.1.4 Results Presentation and Data Export 34
2.2 Development of the offline ZIF Biocomposite Explorer App 37
3 PXRD patterns and analysis 39
3.1 PXRD patterns 39
3.2 TDs of phases 49
3.3 Phase-summary 54
4 Quantification of the protein immobilization 56
4.1 TDs of EE% 56
4.2 TDs of LC% 58
4.3 Relationship Between Amorphous Fraction and LC% 64
5 ATR–IR Spectral Data 68
5.1 Spectra 68
5.2 TDs of IR ratio 78

1 Experimental details

1.1 General remarks

Zinc acetate dihydrate ($Zn(OAc)_2 \cdot 2H_2O$, ACS grade, Merck), 2-methylimidazole (HmIM, 99%, Sigma-Aldrich), bovine serum albumin (BSA, lyophilized powder, ≥96%, Sigma-Aldrich), Bradford reagent (for 0–1.4 mg mL^{-1} protein, Sigma-Aldrich), and ethanol (99%, Roth) were purchased from commercial suppliers and used as received without further purification. Deionized (DI) water was used in all experiments.

1.2 Semi-automated synthesis of ZIF Biocomposites

1.2.1 Synthesis matrix and protocol encoding

A systematic synthesis matrix was designed to explore the predefined variable space with high repeatability. Each sample was assigned a compact identifier:

$$[C - W - P - R]$$

where C denotes the total precursor concentration level (C_1–C_5: 12.5, 25, 50, 75, and 100 mg mL^{-1}), W is the washing regime ("WW" for DI water washing, "EW" for DI water followed by ethanol washing), P denotes the composition coordinate (1–36), and R is the replicate index (Round 1–3).

Based on the design, the experimental matrix comprised five total concentrations (TCs), 36 composition coordinates, and two washing regimes, resulting in a total of $5 \times 36 \times 2 = 360$ unique conditions. Each condition was prepared in triplicate ($n = 3$) under identical automated control scripts, yielding $360 \times 3 = 1080$ individual samples.

All syntheses were executed using a software-controlled syringe-pump platform.[1] Each run was defined by a protocol file encoding: (i) reagent identities and stock concentrations, (ii) dosing order, (iii) flow rates, (iv) inter-step delays, (v) aspiration and dispensing cycles, and (vi) total reaction volume. The reaction volume was fixed at 2.0 mL for all matrix points, ensuring that variations in total precursor concentration corresponded to controlled dilution.

1.2.2 Dosing sequence and reaction conditions

For each synthesis coordinate [*C*–*P*], stock solutions of $Zn(OAc)_2 \cdot 2H_2O$ (M), HmIM (L), and BSA were first prepared in DI water at the required concentrations. The syringe-pump script[1] then automatically dispensed predefined volumes of (i) ligand (L), (ii) DI water, (iii) BSA, and (iv) metal cation (M) into 2.0 mL Eppendorf tubes according to the encoded formulation matrix. The corresponding addition volumes are listed in **Table S2** to **Table S6**. The dosing order was L → water → BSA → M, with a flow rate of 2.0 mL min^{-1}.

After complete addition of all solutions, the reaction mixture was stirred at 1,000 rpm for 60 min using a cylindrical polytetrafluoroethylene magnetic stirrer bar (8 × 3 mm, Cowie, item no. 001.108) on an IKA-COMBIMAG RCT magnetic stirrer to ensure homogeneous mixing. The mixture was subsequently left undisturbed under static conditions for 24 h without further stirring. The resulting solid products were collected by centrifugation (13,000 rpm, 5 min; Eppendorf 5425), and the supernatants were retained for Encapsulation Efficiency (EE%) measurements.

Samples from the ternary-diagram water group (TD-H_2O, WW) were washed with DI water, whereas samples from the ternary-diagram ethanol group (TD-EtOH, EW) were washed sequentially with DI water and ethanol. Finally, all powders were air-dried at room temperature (23 °C) for 48 h. The detailed washing procedure is described in Section 1.3.

1.2.3 Composition map of synthesis coordinates

Table S1. Synthesis conditions for the preparation of the ZIF biocomposites[2]

Sample #	M (wt.%)	L (wt.%)	BSA (wt.%)	L/M mass ratio
1	10	10	80	1.00
2	20	10	70	0.50
3	30	10	60	0.33
4	40	10	50	0.25
5	50	10	40	0.20
6	60	10	30	0.17
7	70	10	20	0.14
8	10	20	70	2.00
9	20	20	60	1.00
10	30	20	50	0.67
11	40	20	40	0.50
12	50	20	30	0.40
13	60	20	20	0.33
14	70	20	10	0.29
15	10	30	60	3.00
16	20	30	50	1.50
17	30	30	40	1.00
18	40	30	30	0.75
19	50	30	20	0.60
20	60	30	10	0.50
21	10	40	50	4.00
22	20	40	40	2.00
23	30	40	30	1.33
24	40	40	20	1.00
25	50	40	10	0.80

Sample #	M (wt.%)	L (wt.%)	BSA (wt.%)	L/M mass ratio
26	10	50	40	5.00
27	20	50	30	2.50
28	30	50	20	1.67
29	40	50	10	1.25
30	10	60	30	6.00
31	20	60	20	3.00
32	30	60	10	2.00
33	10	70	20	7.00
34	20	70	10	3.50
35	10	80	10	8.00
36	80	10	10	0.13

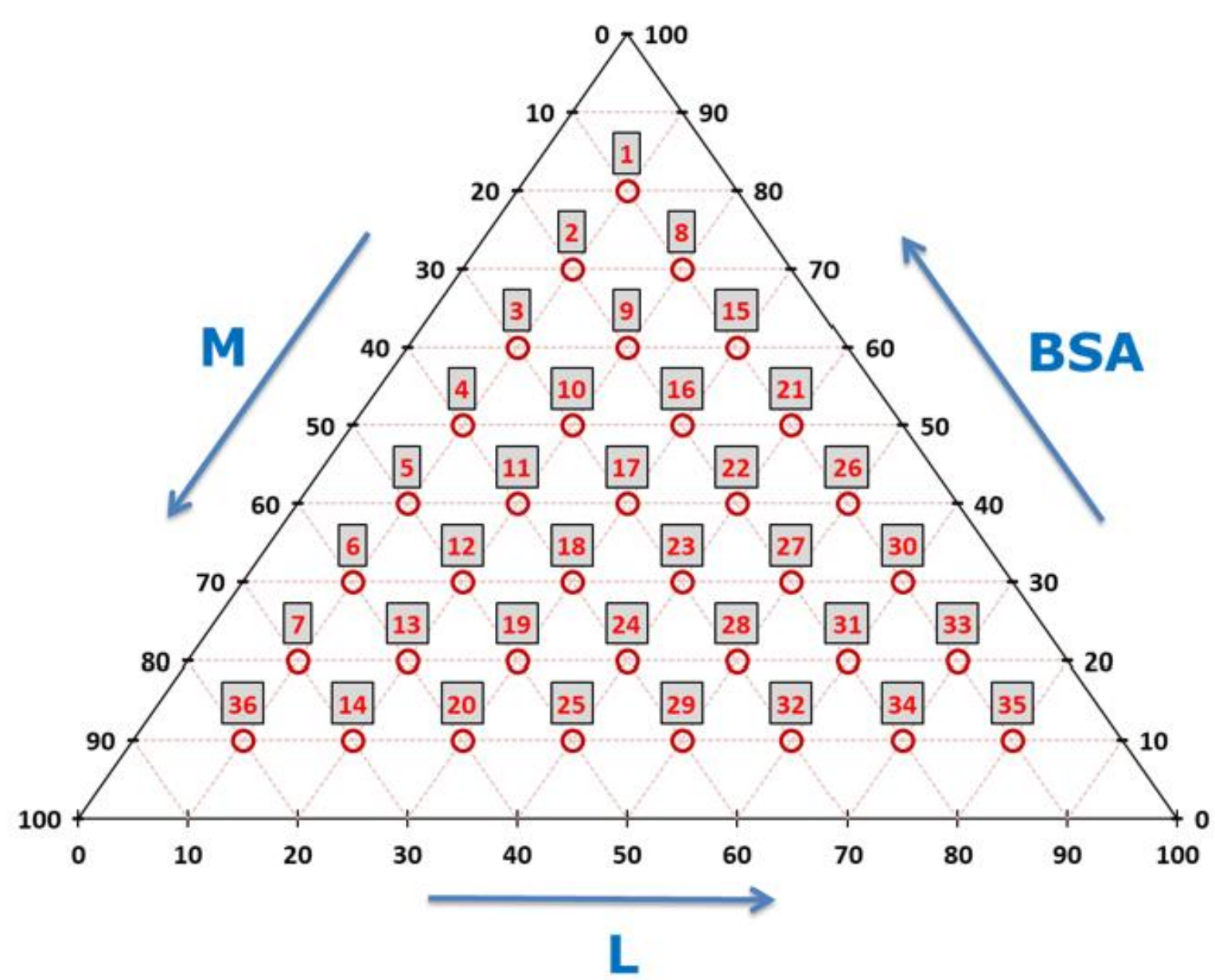

Figure S1. Schematic TD illustrating the weight percentages of M, L, and BSA, together with the sample numbering used in this work.[2]

1.2.4 Concentration-specific dosing tables

Different stock concentrations were used for the five TC levels to ensure reliable liquid delivery by the syringe-pump system while maintaining a constant final sample volume. For each TC level, the concentrations of the metal, ligand, and protein stock solutions were adjusted accordingly, so that the same stock solutions could be used throughout the corresponding set of samples while allowing the required precursor volumes to be dispensed within the reliable operating range of the syringe pumps. Because dispensing volumes below 100 μL resulted in insufficiently precise dosing, lower-concentration stock solutions were used when necessary to increase the required dispensing volumes without altering the target precursor ratios or total precursor concentration.

TC = 12.5 mg mL^{-1} Aqueous stock solutions of $Zn(OAc)_2 \cdot 2H_2O$ (60 mM), 2-methylimidazole (160 mM), and BSA (20 mg mL^{-1}) were prepared and independently delivered using the four-syringe pump system.

Table S2. Synthesis conditions for ZIF biocomposites at TC = 12.5 mg mL^{-1}.

Sample #	M (wt.%)	L (wt.%)	BSA (wt.%)	L/M mass ratio	L (μL)	Water (μL)	BSA (μL)	M (μL)
1	10	10	80	1.00	190	620	1000	190
2	20	10	70	0.50	190	555	875	380
3	30	10	60	0.33	190	491	750	569
4	40	10	50	0.25	190	426	625	759
5	50	10	40	0.20	190	361	500	949
6	60	10	30	0.17	190	296	375	1139
7	70	10	20	0.14	190	231	250	1329
8	10	20	70	2.00	381	554	875	190
9	20	20	60	1.00	381	489	750	380
10	30	20	50	0.67	381	425	625	569

Sample #	M (wt.%)	L (wt.%)	BSA (wt.%)	L/M mass ratio	L (μL)	Water (μL)	BSA (μL)	M (μL)
11	40	20	40	0.50	381	360	500	759
12	50	20	30	0.40	381	295	375	949
13	60	20	20	0.33	381	230	250	1139
14	70	20	10	0.29	381	165	125	1329
15	10	30	60	3.00	571	489	750	190
16	20	30	50	1.50	571	424	625	380
17	30	30	40	1.00	571	360	500	569
18	40	30	30	0.75	571	295	375	759
19	50	30	20	0.60	571	230	250	949
20	60	30	10	0.50	571	165	125	1139
21	10	40	50	4.00	761	424	625	190
22	20	40	40	2.00	761	359	500	380
23	30	40	30	1.33	761	295	375	569
24	40	40	20	1.00	761	230	250	759
25	50	40	10	0.80	761	165	125	949
26	10	50	40	5.00	952	358	500	190
27	20	50	30	2.50	952	293	375	380
28	30	50	20	1.67	952	229	250	569
29	40	50	10	1.25	952	164	125	759
30	10	60	30	6.00	1142	293	375	190
31	20	60	20	3.00	1142	228	250	380
32	30	60	10	2.00	1142	164	125	569
33	10	70	20	7.00	1332	228	250	190
34	20	70	10	3.50	1332	163	125	380
35	10	80	10	8.00	1523	162	125	190
36	80	10	10	0.13	190	166	125	1519

TC = 25 mg mL⁻¹ Aqueous stock solutions of $Zn(OAc)_2 \cdot 2H_2O$ (120 mM), 2-methylimidazole (320 mM), and BSA (30 mg mL^{-1}) were prepared and independently delivered using the four-syringe pump system.

Table S3. Synthesis conditions for ZIF biocomposites at TC = 25 mg mL^{-1}.

Sample #	M (wt.%)	L (wt.%)	BSA (wt.%)	L/M mass ratio	L (μL)	Water (μL)	BSA (μL)	M (μL)
1	10	10	80	1.00	190	287	1333	190
2	20	10	70	0.50	190	263	1167	380
3	30	10	60	0.33	190	241	1000	569
4	40	10	50	0.25	190	218	833	759
5	50	10	40	0.20	190	194	667	949
6	60	10	30	0.17	190	171	500	1139
7	70	10	20	0.14	190	148	333	1329
8	10	20	70	2.00	381	262	1167	190
9	20	20	60	1.00	381	239	1000	380
10	30	20	50	0.67	381	217	833	569
11	40	20	40	0.50	381	193	667	759
12	50	20	30	0.40	381	170	500	949
13	60	20	20	0.33	381	147	333	1139
14	70	20	10	0.29	381	123	167	1329
15	10	30	60	3.00	571	239	1000	190
16	20	30	50	1.50	571	216	833	380
17	30	30	40	1.00	571	193	667	569
18	40	30	30	0.75	571	170	500	759
19	50	30	20	0.60	571	147	333	949
20	60	30	10	0.50	571	123	167	1139
21	10	40	50	4.00	761	216	833	190
22	20	40	40	2.00	761	192	667	380

Sample #	M (wt.%)	L (wt.%)	BSA (wt.%)	L/M mass ratio	L (μL)	Water (μL)	BSA (μL)	M (μL)
23	30	40	30	1.33	761	170	500	569
24	40	40	20	1.00	761	147	333	759
25	50	40	10	0.80	761	123	167	949
26	10	50	40	5.00	952	191	667	190
27	20	50	30	2.50	952	168	500	380
28	30	50	20	1.67	952	146	333	569
29	40	50	10	1.25	952	122	167	759
30	10	60	30	6.00	1142	168	500	190
31	20	60	20	3.00	1142	145	333	380
32	30	60	10	2.00	1142	122	167	569
33	10	70	20	7.00	1332	145	333	190
34	20	70	10	3.50	1332	121	167	380
35	10	80	10	8.00	1523	120	167	190
36	80	10	10	0.13	190	124	167	1519

TC = 50 mg mL⁻¹ Aqueous stock solutions of $Zn(OAc)_2 \cdot 2H_2O$ (240 mM), 2-methylimidazole (1280 mM), and BSA (50 mg mL^{-1}) were prepared and independently delivered using the four-syringe pump system.

Table S4. Synthesis conditions for ZIF biocomposites at TC = 50 mg mL^{-1}.

Sample #	M (wt.%)	L (wt.%)	BSA (wt.%)	L/M mass ratio	L (μL)	Water (μL)	BSA (μL)	M (μL)
1	10	10	80	1.00	95	115	1600	190
2	20	10	70	0.50	95	125	1400	380
3	30	10	60	0.33	95	136	1200	569
4	40	10	50	0.25	95	146	1000	759
5	50	10	40	0.20	95	156	800	949

Sample #	M (wt.%)	L (wt.%)	BSA (wt.%)	L/M mass ratio	L (μL)	Water (μL)	BSA (μL)	M (μL)
6	60	10	30	0.17	95	166	600	1139
7	70	10	20	0.14	95	176	400	1329
8	10	20	70	2.00	190	220	1400	190
9	20	20	60	1.00	190	230	1200	380
10	30	20	50	0.67	190	241	1000	569
11	40	20	40	0.50	190	251	800	759
12	50	20	30	0.40	190	261	600	949
13	60	20	20	0.33	190	271	400	1139
14	70	20	10	0.29	190	281	200	1329
15	10	30	60	3.00	285	325	1200	190
16	20	30	50	1.50	285	335	1000	380
17	30	30	40	1.00	285	346	800	569
18	40	30	30	0.75	285	356	600	759
19	50	30	20	0.60	285	366	400	949
20	60	30	10	0.50	285	376	200	1139
21	10	40	50	4.00	381	429	1000	190
22	20	40	40	2.00	381	439	800	380
23	30	40	30	1.33	381	450	600	569
24	40	40	20	1.00	381	460	400	759
25	50	40	10	0.80	381	470	200	949
26	10	50	40	5.00	476	534	800	190
27	20	50	30	2.50	476	544	600	380
28	30	50	20	1.67	476	555	400	569
29	40	50	10	1.25	476	565	200	759
30	10	60	30	6.00	571	639	600	190
31	20	60	20	3.00	571	649	400	380
32	30	60	10	2.00	571	660	200	569

Sample #	M (wt.%)	L (wt.%)	BSA (wt.%)	L/M mass ratio	L (µL)	Water (µL)	BSA (µL)	M (µL)
33	10	70	20	7.00	666	744	400	190
34	20	70	10	3.50	666	754	200	380
35	10	80	10	8.00	761	849	200	190
36	80	10	10	0.13	95	186	200	1519

TC = 75 mg mL⁻¹ Aqueous stock solutions of $Zn(OAc)_2 \cdot 2H_2O$ (640 mM), 2-methylimidazole (1800 mM), and BSA (75 mg mL^{-1}) were prepared and independently delivered using the four-syringe pump system.

Table S5. Synthesis conditions for ZIF biocomposites at TC = 75 mg mL^{-1}.

Sample #	M (wt.%)	L (wt.%)	BSA (wt.%)	L/M mass ratio	L (µL)	Water (µL)	BSA (µL)	M (µL)
1	10	10	80	1.00	102	191	1600	107
2	20	10	70	0.50	102	284	1400	214
3	30	10	60	0.33	102	378	1200	320
4	40	10	50	0.25	102	471	1000	427
5	50	10	40	0.20	102	564	800	534
6	60	10	30	0.17	102	657	600	641
7	70	10	20	0.14	102	751	400	747
8	10	20	70	2.00	203	290	1400	107
9	20	20	60	1.00	203	383	1200	214
10	30	20	50	0.67	203	477	1000	320
11	40	20	40	0.50	203	570	800	427
12	50	20	30	0.40	203	663	600	534
13	60	20	20	0.33	203	756	400	641
14	70	20	10	0.29	203	850	200	747
15	10	30	60	3.00	305	388	1200	107

16	20	30	50	1.50	305	481	1000	214
17	30	30	40	1.00	305	575	800	320
18	40	30	30	0.75	305	668	600	427
19	50	30	20	0.60	305	761	400	534
20	60	30	10	0.50	305	854	200	641
21	10	40	50	4.00	406	487	1000	107
22	20	40	40	2.00	406	580	800	214
23	30	40	30	1.33	406	674	600	320
24	40	40	20	1.00	406	767	400	427
25	50	40	10	0.80	406	860	200	534
26	10	50	40	5.00	508	585	800	107
27	20	50	30	2.50	508	678	600	214
28	30	50	20	1.67	508	772	400	320
29	40	50	10	1.25	508	865	200	427
30	10	60	30	6.00	609	684	600	107
31	20	60	20	3.00	609	777	400	214
32	30	60	10	2.00	609	871	200	320
33	10	70	20	7.00	711	782	400	107
34	20	70	10	3.50	711	875	200	214
35	10	80	10	8.00	812	881	200	107
36	80	10	10	0.13	102	844	200	854

TC = 100 mg mL^{-1} Aqueous stock solutions of $Zn(OAc)_2 \cdot 2H_2O$ (640 mM), 2-methylimidazole (2560 mM), and BSA (100 mg mL^{-1}) were prepared and independently delivered using the four-syringe pump system.

Table S6. Synthesis conditions for ZIF biocomposites at TC = 100 mg mL^{-1}.

Sample #	M (wt.%)	L (wt.%)	BSA (wt.%)	L/M mass ratio	L (μL)	Water (μL)	BSA (μL)	M (μL)
1	10	10	80	1.00	95	163	1600	142
2	20	10	70	0.50	95	220	1400	285
3	30	10	60	0.33	95	278	1200	427
4	40	10	50	0.25	95	336	1000	569
5	50	10	40	0.20	95	393	800	712
6	60	10	30	0.17	95	451	600	854
7	70	10	20	0.14	95	508	400	997
8	10	20	70	2.00	190	268	1400	142
9	20	20	60	1.00	190	325	1200	285
10	30	20	50	0.67	190	383	1000	427
11	40	20	40	0.50	190	441	800	569
12	50	20	30	0.40	190	498	600	712
13	60	20	20	0.33	190	556	400	854
14	70	20	10	0.29	190	613	200	997
15	10	30	60	3.00	285	373	1200	142
16	20	30	50	1.50	285	430	1000	285
17	30	30	40	1.00	285	488	800	427
18	40	30	30	0.75	285	546	600	569
19	50	30	20	0.60	285	603	400	712
20	60	30	10	0.50	285	661	200	854
21	10	40	50	4.00	381	477	1000	142
22	20	40	40	2.00	381	534	800	285
23	30	40	30	1.33	381	592	600	427
24	40	40	20	1.00	381	650	400	569
25	50	40	10	0.80	381	707	200	712
26	10	50	40	5.00	476	582	800	142
27	20	50	30	2.50	476	639	600	285

Sample #	M (wt.%)	L (wt.%)	BSA (wt.%)	L/M mass ratio	L (µL)	Water (µL)	BSA (µL)	M (µL)
28	30	50	20	1.67	476	697	400	427
29	40	50	10	1.25	476	755	200	569
30	10	60	30	6.00	571	687	600	142
31	20	60	20	3.00	571	744	400	285
32	30	60	10	2.00	571	802	200	427
33	10	70	20	7.00	666	792	400	142
34	20	70	10	3.50	666	849	200	285
35	10	80	10	8.00	761	897	200	142
36	80	10	10	0.13	95	566	200	1139

1.3 Washing procedures

Depending on the assigned ternary-diagram (TD) group, the washing process was performed using either the TD-H_2O (WW) procedure or the TD-EtOH (EW) procedure:

TD-H_2O. The solid product was separated via centrifugation (13,000 rpm for 5 min), and the reaction supernatant was retained for subsequent determination of EE%. The powder was re-suspended in 2.0 mL DI water using a vortex mixer (3,000 rpm for 1 min). The suspension was centrifuged (13,000 rpm for 5 min) to yield a pellet, and the supernatant was discarded. This procedure was repeated 3 times (a total of 4 DI-washing steps including the initial separation). Finally, the powder was air-dried for 48 h at room temperature (23 °C). [2]

TD-EtOH. The solid product was separated via centrifugation (13,000 rpm for 5 min), and the reaction supernatant was retained for subsequent determination of EE%. The powder was re-suspended in 2.0 mL DI water using a vortex mixer (3,000 rpm for 1 min), then centrifuged (13,000 rpm for 5 min) and the supernatant was discarded. This water washing was repeated twice (3 DI-water washing steps in total including the initial separation). Afterwards, the pellet was re-

suspended in 2.0 mL ethanol (EtOH) using a vortex mixer (3,000 rpm for 1 min), centrifuged (13,000 rpm for 5 min), and the supernatant was discarded. This ethanol washing was repeated once (2 EtOH washing steps in total). Finally, the powder was air-dried for 48 h at room temperature (23 °C). [2]

1.4 Characterization

Powder X-ray diffraction (PXRD) patterns were collected at room temperature using a Rigaku SmartLab X-ray Diffractometer (Cu Kα radiation, λ = 1.5406 Å) with a step size of 0.01°, a scanning range of 5–20°, and a scanning rate of 6.0 ° min^{-1}.

Infrared (IR) measurements were carried out at room temperature using a Bruker Alpha spectrometer in Attenuated Total Reflectance (ATR) mode with a diamond window, with a resolution of 2 cm^{-1}, 32 acquisitions, and a spectral range of 4,000–400 cm^{-1}.

Spectra were used as compositional checks to confirm: (i) framework formation (e.g., Zn–N bands characteristic of imidazolate coordination) and (ii) guest immobilisation (Amide I and Amide II bands for proteins, where relevant). Where needed, spectra were baseline-corrected and normalised to enable qualitative comparison across the mapped synthesis space.

1.5 PXRD-based amorphous fraction estimation

To estimate the crystalline and amorphous fractions of each biocomposite from the PXRD patterns, the following systematic analysis procedure was applied. Samples that did not show diffraction peaks and were classified as "amorphous" by the ZIF phase analysis app (https://rapps.tugraz.at/apps/porousbiotech/ZIFphaseanalysis/) were excluded from this analysis.[3] First, the background pattern collected from a clean silicon wafer without sample was subtracted from the

measured PXRD pattern. This step was used to remove the instrumental and background contribution before further analysis.

After background subtraction, the PXRD pattern was analysed over the selected 2θ range of 5–20°. The total diffraction area, A_{total}, was calculated by integrating the complete background subtracted curve:

$$A_{total} = \int I_{corr}(2\theta)d(2\theta)$$

where $I_{corr}(2\theta)$ is the PXRD intensity after subtraction of the background.

To estimate the crystalline contribution, we fitted an asymmetric least-squares (ALS) baseline to the background-subtracted PXRD pattern. The ALS baseline was calculated using p = 0.015 and $\lambda = 10^6$. This baseline was used to follow the broad underlying scattering profile, while the sharper Bragg reflections above the baseline were assigned to the crystalline contribution. The crystalline area, $A_{\text{crystalline}}$, was then calculated by integrating only the peak area above the fitted ALS baseline:

$$A_{\text{crystalline}} = \int [I_{corr}(2\theta) - I_{ALS}(2\theta)]\, d(2\theta)$$

where $I_{ALS}(2\theta)$ represents the fitted ALS baseline.

The crystallinity ratio was defined as the ratio between the crystalline peak area and the total PXRD area:

$$\text{Crystallinity ratio} = A_{\text{crystalline}}/A_{total}$$

The amorphous fraction was then calculated as:

$$\text{Amorphous fraction} = 1 - \text{Crystallinity ratio}$$

This approach provides a relative PXRD-based descriptor of the amorphous contribution in each sample. A crystallinity ratio close to 1 indicates that most of the diffraction intensity arises from crystalline Bragg reflections, whereas a low crystallinity ratio indicates a larger contribution from broad amorphous scattering. Accordingly, an amorphous fraction close to 1 suggests a predominantly amorphous sample, while a value close to 0 suggests a predominantly crystalline material.

It should be noted that this method provides a qualitative estimate rather than an absolute crystallinity value or a calibrated amorphous mass fraction.

The same processing parameters and integration range were applied consistently to all PXRD patterns to allow comparison across the dataset. Comparison with LC% therefore provides a qualitative assessment of the protein and framework contributions, subject to differences in scattering power and experimental uncertainty.

1.6 ATR-IR band-ratio analysis (IR ratio)

To obtain a semi-quantitative descriptor of the relative protein contribution in the ZIF biocomposites, an ATR-IR band-ratio analysis was performed for each spectrum. Two characteristic wavenumbers were selected: ~ 421 cm^{-1}, assigned to the Zn–N vibration of the ZIF framework, and ~ 1650 cm^{-1}, corresponding to the amide I band of BSA. For each sample, the absorbance values at these two positions were extracted from the ATR-IR spectrum and used to calculate the IR band ratio according to the following equation:

$$\text{IR ratio} = ABS_{1650}/(ABS_{421} + ABS_{1650})$$

where ABS_{1650} is the absorbance at ~ 1650 cm^{-1} and ABS_{421} is the absorbance at ~ 421 cm^{-1}. For visualization, the IR ratio is expressed as a percentage by multiplying by 100.

This ratio provides a qualitative indication of the relative contribution of protein and ZIF framework bands in the biocomposite. A value close to 1 indicates that the amide I band dominates the selected spectral features, suggesting a stronger protein contribution. In contrast, a value close to 0 indicates that the Zn–N stretching vibration is dominant, suggesting a stronger ZIF contribution.

It should be noted that this IR ratio is used as a qualitative descriptor rather than an absolute measurement of protein loading. Therefore, the IR band ratio was mainly used to compare trends across the sample library and to support the interpretation of protein incorporation together with encapsulation efficiency, loading capacity, and PXRD-derived phase information.

1.7 EE%

EE% was quantified by measuring the amount of non-immobilised biomolecule remaining in the reaction supernatant after synthesis and before washing. The reaction supernatant was analysed using the Bradford assay, with calibration curves prepared from the same biomolecule used in the synthesis, for example BSA standards in the 0–1.4 mg mL^{-1} range. Absorbance was recorded at 595 nm using a microplate reader (VANTAstar, BMG LABTECH) with blank subtraction using solvent and reagent controls. EE% was calculated as follows: [2]

$$EE\% = 100 \times (m_0 - m_{lost})/m_0$$

where m_0 is the initial mass of biomolecule dosed into the synthesis.

m_{lost} is the mass of non-immobilized biomolecule remaining in the reaction supernatant.

1.8 ICP-OES Analysis

The sulfur concentration was quantified by a radially viewed Inductively Coupled Plasma-Optical Emission Spectrometer (ICP-OES, Arcos II, Spectro, Germany). Approximately 1.0 mg of biocomposite sample was accurately weighed and dissolved in 2.0 mL of 0.1 M HCl. The solution was subsequently diluted to 10.0 mL with ultrapure water. Sulfur quantification was carried out using the S(I) 180.731 nm emission line. Scandium was employed as an internal standard at a concentration of 1.0 mg L^{-1}.

1.9 Loading capacity (LC%)

LC% of BSA in the biocomposites was calculated based on sulfur quantification according to the following equation: [2,4]

$$LC\% = 100 \times \left(m_{BSA}/m_{\text{biocomposite}}\right)$$

where:

$$m_{BSA} = m_S/w_S = (c_S \times V)/w_S$$

Thus:

$$LC\% = 100 \times \left[(c_S \times V)/\left(w_S \times m_{\text{biocomposite}}\right)\right]$$

where:

m_{BSA} is the mass of BSA present in the analysed sample.

$m_{\text{biocomposite}}$ is the mass of the biocomposite used for ICP-OES analysis.

m_S is the sulfur mass determined in the sample.

c_S is the sulfur concentration measured by ICP-OES.

V is the final solution volume after digestion.

w_S is the corrected experimentally determined sulfur mass fraction in pure BSA, measured under identical digestion and ICP-OES conditions.

1.10 Comparison of synthesis with and without stirring

To evaluate the influence of initial mixing on the formation of protein@ZIF biocomposite phases, samples P31, P32, and P34 were selected for comparison. For the stirred samples, the synthesis was conducted according to the procedure described in section 1.2.2, which includes 60 min of stirring during the initial stage. In contrast, non-stirred samples were prepared using the same precursor dosing sequence but were left undisturbed after mixing. After 24 h of incubation, all samples were collected and washed following the TD-H_2O protocol. PXRD was employed to characterize the resulting biocomposite phases. The corresponding diffraction patterns and phase assignments are presented in **Figure S2** to **Figure S6** and **Table S7** to **Table S11**.

Compared to samples subjected to initial stirring (**Figure 2**), those prepared without the 60 min stirring step exhibited markedly reduced crystallinity and poorer reproducibility. This effect was particularly pronounced at lower total concentrations (TC = 12.5 and 25 mg mL^{-1}), likely due to insufficient homogenization of the precursors. As shown in **Figure S2d** and **Figure S3d**, **Figure S3e**, samples 12.5-31, 25-31, and 25-32 displayed broad and weak diffraction features, hindering reliable phase identification. Furthermore, the absence of stirring led to significant variability between replicates, limiting the ability to establish clear correlations between precursor concentration and resulting biocomposite phases. For instance, the triplicate samples of 75-34 yielded distinctly different crystalline phases (**Figure S5f** and **Table S10**).

These results highlight that homogeneous precursor mixing, achieved through initial stirring, is critical for improving both the crystallinity and reproducibility of protein@ZIF biocomposites, thereby enabling more reliable and controllable synthesis.

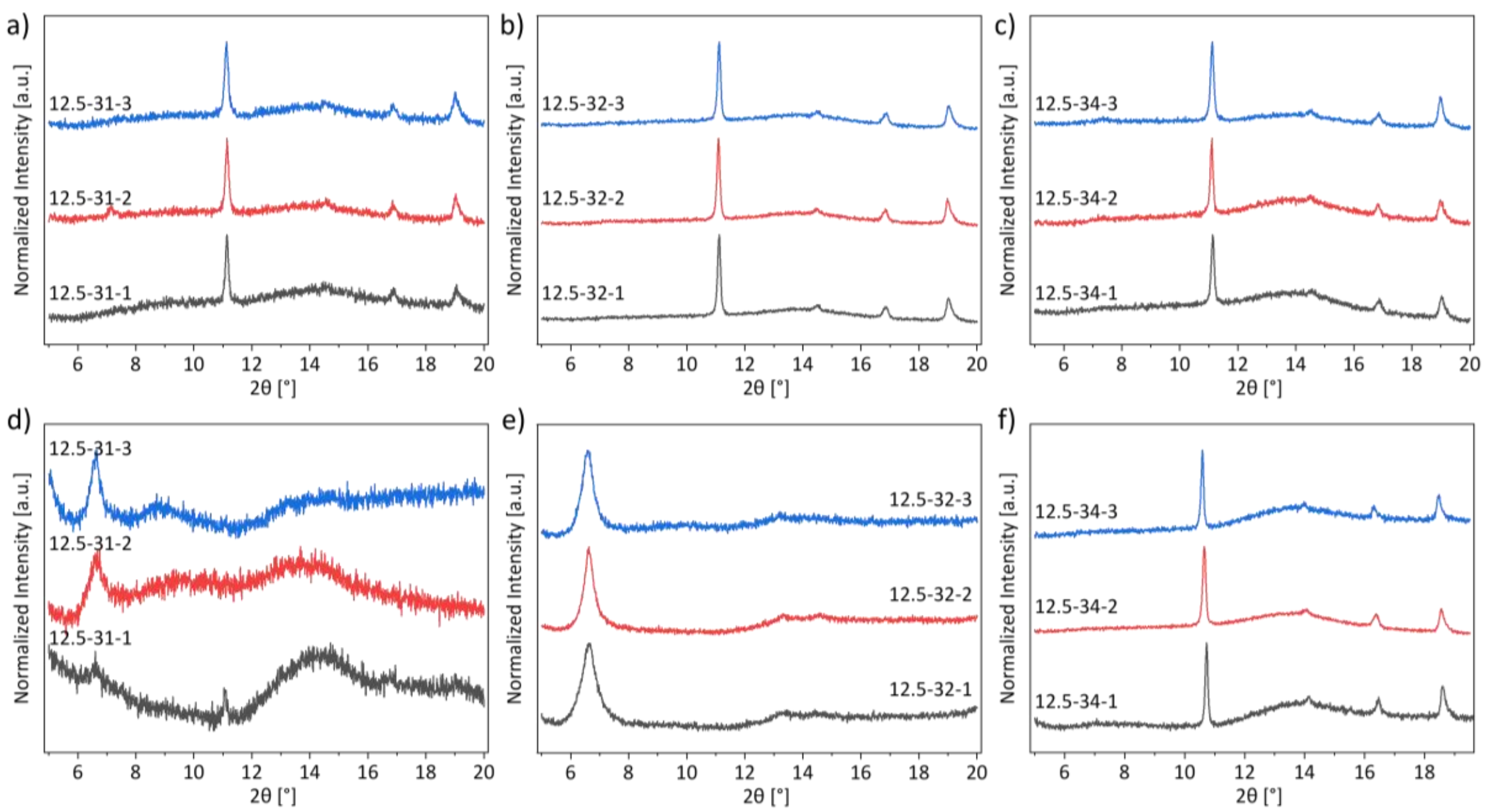

Figure S2. PXRD comparison of samples prepared with and without a stirring step at TC = 12.5 mg mL^{-1}. a) to c) are samples with stirring, d) to f) are samples without stirring.

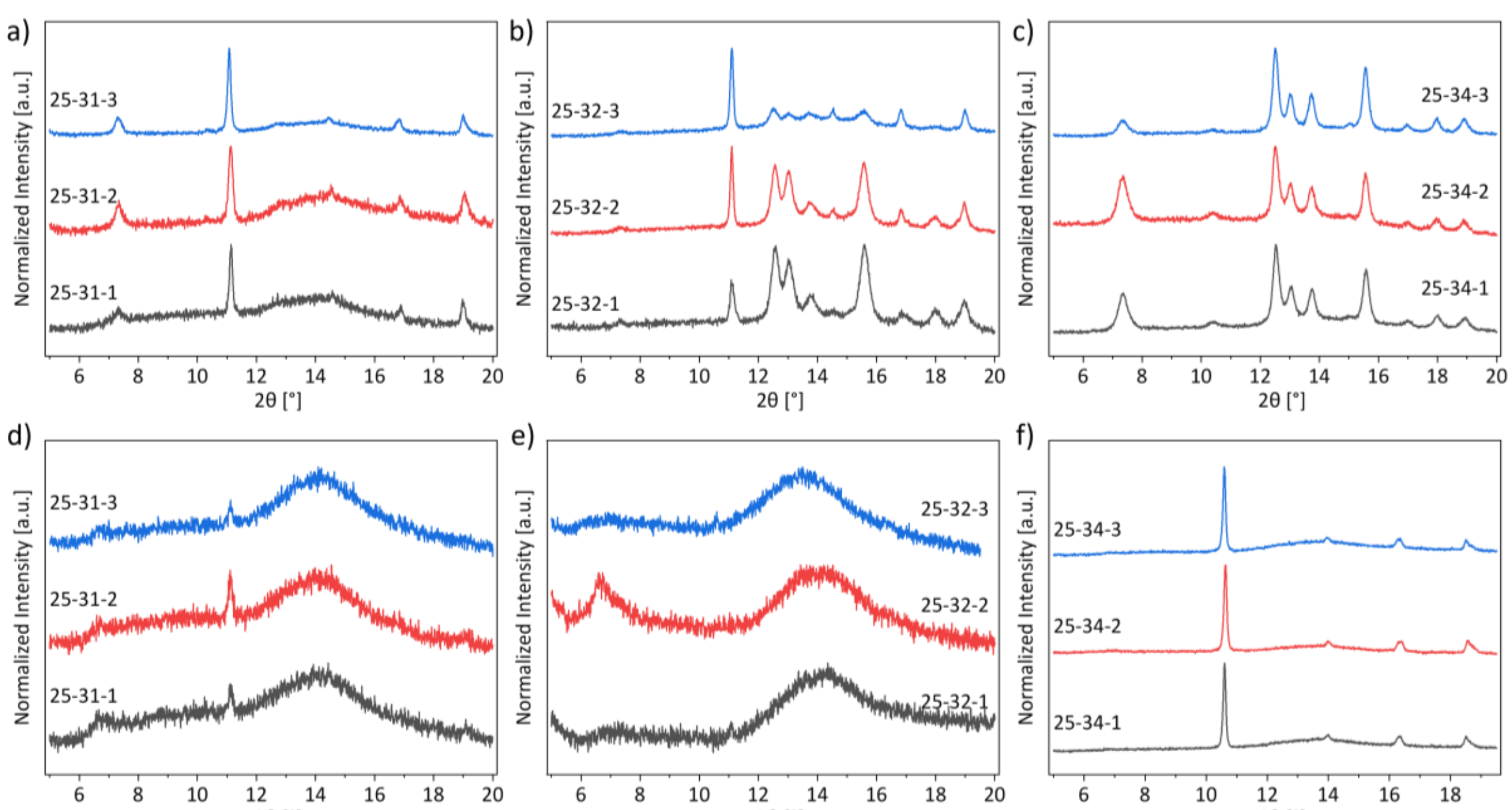

Figure S3. PXRD comparison of samples prepared with and without a stirring step at TC = 25 mg mL^{-1}. a) to c) are samples with stirring, d) to f) are samples without stirring.

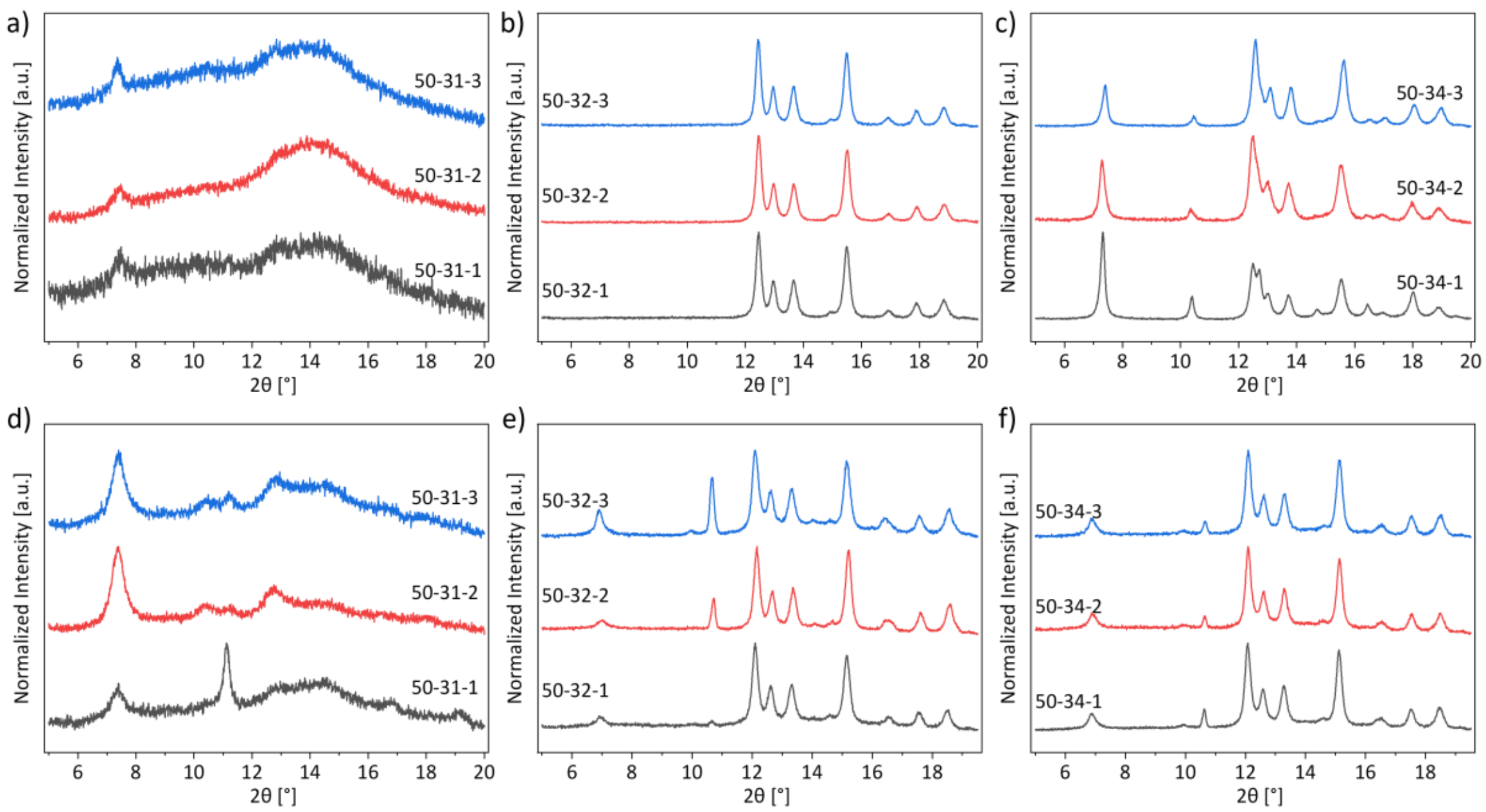

Figure S4. PXRD comparison of samples prepared with and without a stirring step at TC = 50 mg mL^{-1}. a) to c) are samples with stirring, d) to f) are samples without stirring.

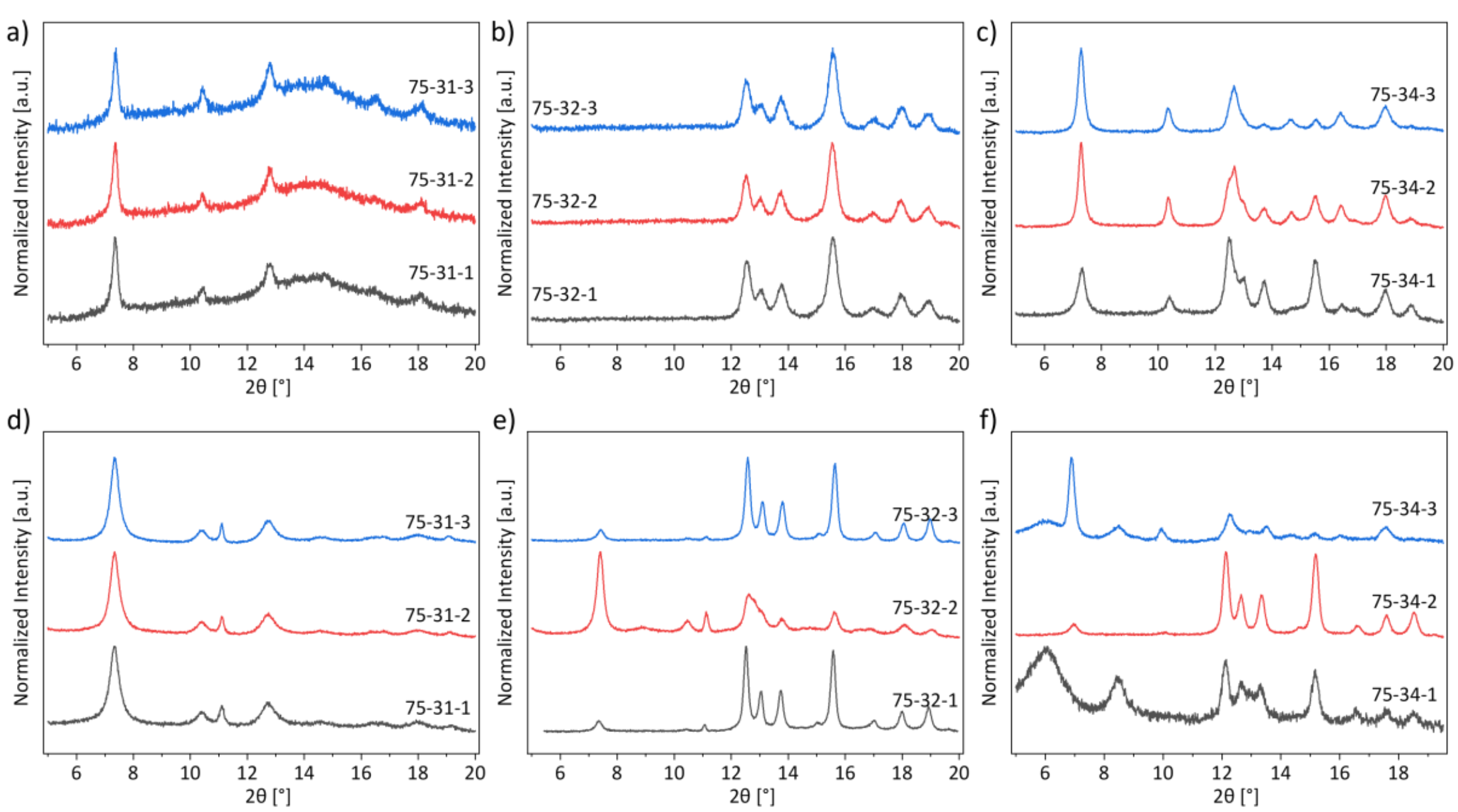

Figure S5. PXRD comparison of samples prepared with and without a stirring step at TC = 75 mg mL^{-1}. a) to c) are samples with stirring, d) to f) are samples without stirring.

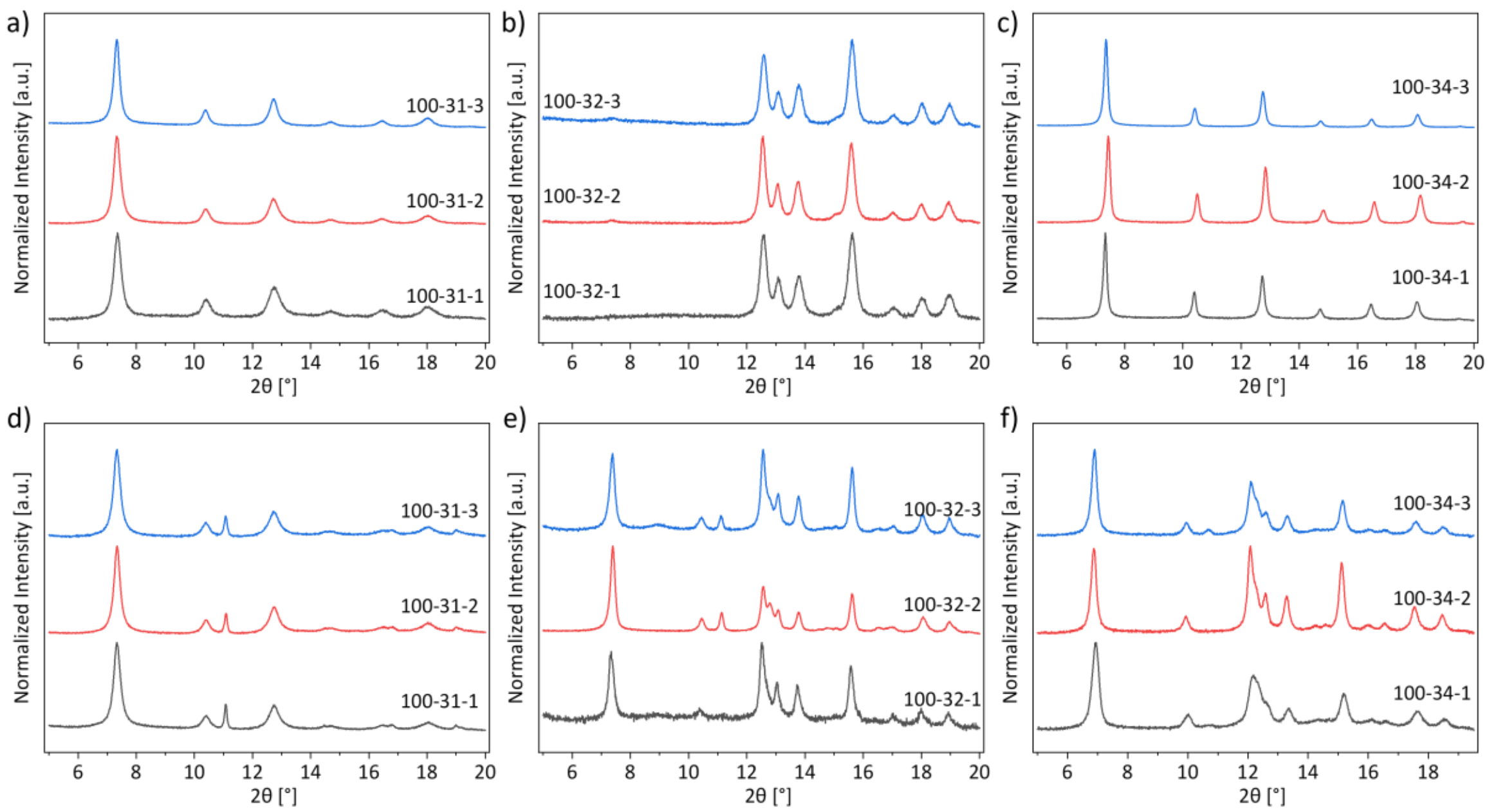

Figure S6. PXRD comparison of samples prepared with and without a stirring step at TC = 100 mg mL^{-1}. a) to c) are samples with stirring, d) to f) are samples without stirring.

Table S7. Phase percentages for samples prepared at 12.5 mg mL^{-1} with and without stirring.

	Stirring	Without stirring
12.5-31-1	100% **ZIF-C**	68% **U13** + 32% **ZIF-C**
12.5-31-2	100% **ZIF-C**	100% **U13**
12.5-31-3	100% **ZIF-C**	100% **U13**
12.5-32-1	100% **ZIF-C**	100% **U13**
12.5-32-2	100% **ZIF-C**	100% **U13**
12.5-32-3	100% **ZIF-C**	100% **U13**
12.5-34-1	100% **ZIF-C**	100% **ZIF-C**

12.5-34-2	100% **ZIF-C**	100% **ZIF-C**
12.5-34-3	100% **ZIF-C**	100% **ZIF-C**

Table S8. Phase percentages for samples prepared at 25 mg mL^{-1} with and without stirring.

	Stirring	Without stirring
25-31-1	19% **sod** + 81% **ZIF-C**	100% **ZIF-C**
25-31-2	20% **sod** + 80% **ZIF-C**	100% **ZIF-C**
25-31-3	14% **sod** + 86% **ZIF-C**	100% **ZIF-C**
25-32-1	80% **dia** + 20% **ZIF-C**	100% amorphous
25-32-2	52% **dia** + 48% **ZIF-C**	100% **U13**
25-32-3	30% **dia** + 70% **ZIF-C**	100% amorphous
25-34-1	27% **sod** + 73% **dia**	100% **ZIF-C**
25-34-2	37% **sod** + 63% **dia**	100% **ZIF-C**
25-34-3	9% **sod** + 91% **dia**	100% **ZIF-C**

Table S9. Phase percentages for samples prepared at 50 mg mL^{-1} with and without stirring.

	Stirring	Without stirring
50-31-1	100% **sod**	26% **sod** + 74% **ZIF-C**
50-31-2	100% **sod**	98% **sod** + 2% **ZIF-C**
50-31-3	100% **sod**	96% **sod** + 4% **ZIF-C**
50-32-1	100% **dia**	4% **sod** + 94% **dia** + 3% **ZIF-C**
50-32-2	100% **dia**	5% **sod** + 75% **dia** + 20% **ZIF-C**
50-32-3	100% **dia**	6% **sod** + 63% **dia** + 31% **ZIF-C**

50-34-1	39% **sod** + 61% **dia**	7% **sod** + 85% **dia** + 8% **ZIF-C**
50-34-2	24% **sod** + 76% **dia**	7% **sod** + 86% **dia** + 7% **ZIF-C**
50-34-3	15% **sod** + 85% **dia**	5% **sod** + 85% **dia** + 10% **ZIF-C**

Table S10. Phase percentages for samples prepared at 75 mg mL^{-1} with and without stirring.

	Stirring	Without stirring
75-31-1	100% **sod**	80% **sod** + 20% **ZIF-C**
75-31-2	100% **sod**	84% **sod** + 16% **ZIF-C**
75-31-3	100% **sod**	71% **sod** + 29% **ZIF-C**
75-32-1	100% **dia**	4% **sod** + 93% **dia** + 4% **ZIF-C**
75-32-2	100% **dia**	56% **sod** + 28% **dia** + 16% **ZIF-C**
75-32-3	100% **dia**	4% **sod** + 95% **dia** + 2% **ZIF-C**
75-34-1	21% **sod** + 79% **dia**	46% **dia** + 54% **U13**
75-34-2	74% **sod** + 26% **dia**	10% **sod** + 90% **dia**
75-34-3	83% **sod** + 17% **dia**	85% **sod** + 15% **dia**

Table S11. Phase percentages for samples prepared at 100 mg mL^{-1} with and without stirring.

	Stirring	Without stirring
100-31-1	100% **sod**	81% **sod** + 19% **ZIF-C**
100-31-2	100% **sod**	83% **sod** + 17% **ZIF-C**
100-31-3	100% **sod**	84% **sod** + 16% **ZIF-C**
100-32-1	100% **dia**	32% **sod** + 68% **dia**
100-32-2	100% **dia**	42% **sod** + 46% **dia** + 12% **ZIF-C**
100-32-3	100% **dia**	32% **sod** + 61% **dia** + 8% **ZIF-C**

100-34-1	100% **sod**	74% **sod** + 26% **dia**
100-34-2	100% **sod**	52% **sod** + 48% **dia**
100-34-3	100% **sod**	63% **sod** + 37% **dia**

1.11 Comparison of semi-automated and manual workflows

A systematic comparison of the effect of total concentration (TC = 25, 50, 75 mg mL^{-1}) under identical L/BSA/M conditions (**Figure S7a–f**) reveals pronounced differences between the semi-automated workflow and two independent manual protocols (manual 1 and manual 2). All samples were subjected to the same water washing procedure. In the P17 system (**Figure S7a–c**), the semi-automated approach reproducibly yields mixed **ZIF-C/sod** phases at 25-17 to 75-17 across all three replicates. In contrast, manual 2 produces pure **ZIF-C** under all conditions (25-17 to 75-17), indicating different phase selection under these conditions. Although manual 1 occasionally yields **ZIF-C/sod** mixtures at 25-17 and 75-17, the results are not reproducible, as pure **ZIF-C** is obtained in Round 1 for both conditions. A similar trend is observed in the P34 system (**Figure S7d–f**). The semi-automated workflow consistently produces **sod/dia** mixed phases at 25-34, 50-34, and 75-34 across all replicates. By contrast, manual 2 yields pure **dia** at 25-34, indicating a clear shift in phase selection. For manual 1, although a **sod/dia** mixture is observed in Round 1, the **sod** fraction is extremely low, and pure **dia** is obtained in Rounds 2 and 3. Overall, manual 1 also tends toward single-phase **dia** formation under this condition, further demonstrating greater variability in the results in the mixed-phase region.

Further comparison at a fixed total concentration (TC = 50 mg mL^{-1}) with varying L/BSA/M ratios (**Figure S7g–i**) shows that P31, P32, and P33 synthesized via semi-automated and manual 2 workflows are largely consistent, yielding pure **sod** (P31 and P33) or **dia** (P32) phases. This suggests that the system becomes less sensitive to the mode of addition when operating away from

phase competition boundaries. However, manual 1 exhibits clear reproducibility issues in the P31 system: although **sod** is obtained in two replicates (consistent with semi-automated and manual 2), **ZIF-C** is formed in Round 1, indicating that manual operation introduces stochastic variability near phase boundaries.

This variability becomes more evident when considering the EE% and its relative standard deviation (RSD), where RSD is calculated by dividing the standard deviation (SD) by the mean EE%. The semi-automated workflow maintains consistently low RSD values across most conditions with ~90% of the tested conditions showing moderate RSDs (e.g. values below 0.01 at 25-34 and 50-34), indicating improved reproducibility with respect to the manual procedure: both manual 1 and manual 2 exhibit significantly higher RSD values, particularly under low EE% conditions. For example, at 75-34, manual 2 shows an SD of 8.4%, comparable to other conditions; however, due to the low EE% value (20.6%), the resulting RSD is substantially amplified. This demonstrates that absolute variability (i.e., SD) alone is insufficient to evaluate system stability, whereas RSD provides a more reliable measure of reproducibility. Overall, compared to manual methods, the semi-automated workflow exhibits reduced variation in EE% and significantly lower RSD values, indicating a more stable and reproducible synthesis process. This improvement can be attributed to the precise control of precursor addition provided by the syringe pump, which ensures a constant dosing rate and could minimize fluctuations in local supersaturation during nucleation and growth steps. In contrast, manual pipetting inevitably introduces variations in addition rate and mixing conditions, leading to increased experimental variability.

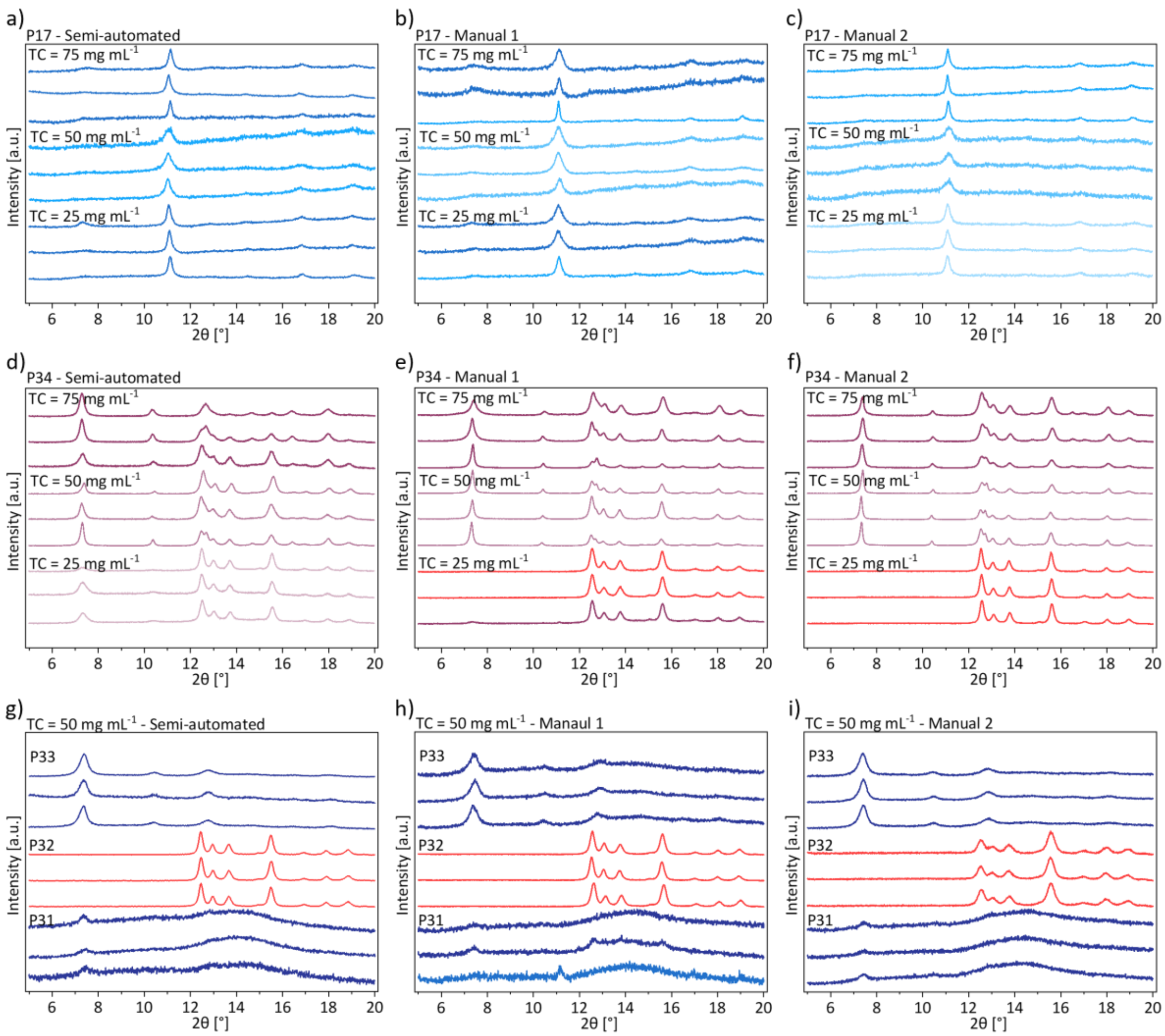

Figure S7. Comparison of triplicate PXRD patterns obtained using semi-automated, manual 1, and manual 2 workflows. a)–c) P17 at TC = 25, 50, and 75 mg mL^{-1}; d)–f) P34 at TC = 25, 50, and 75 mg mL^{-1}; and g)–i) P31, P32, and P33 at TC = 50 mg mL^{-1}.

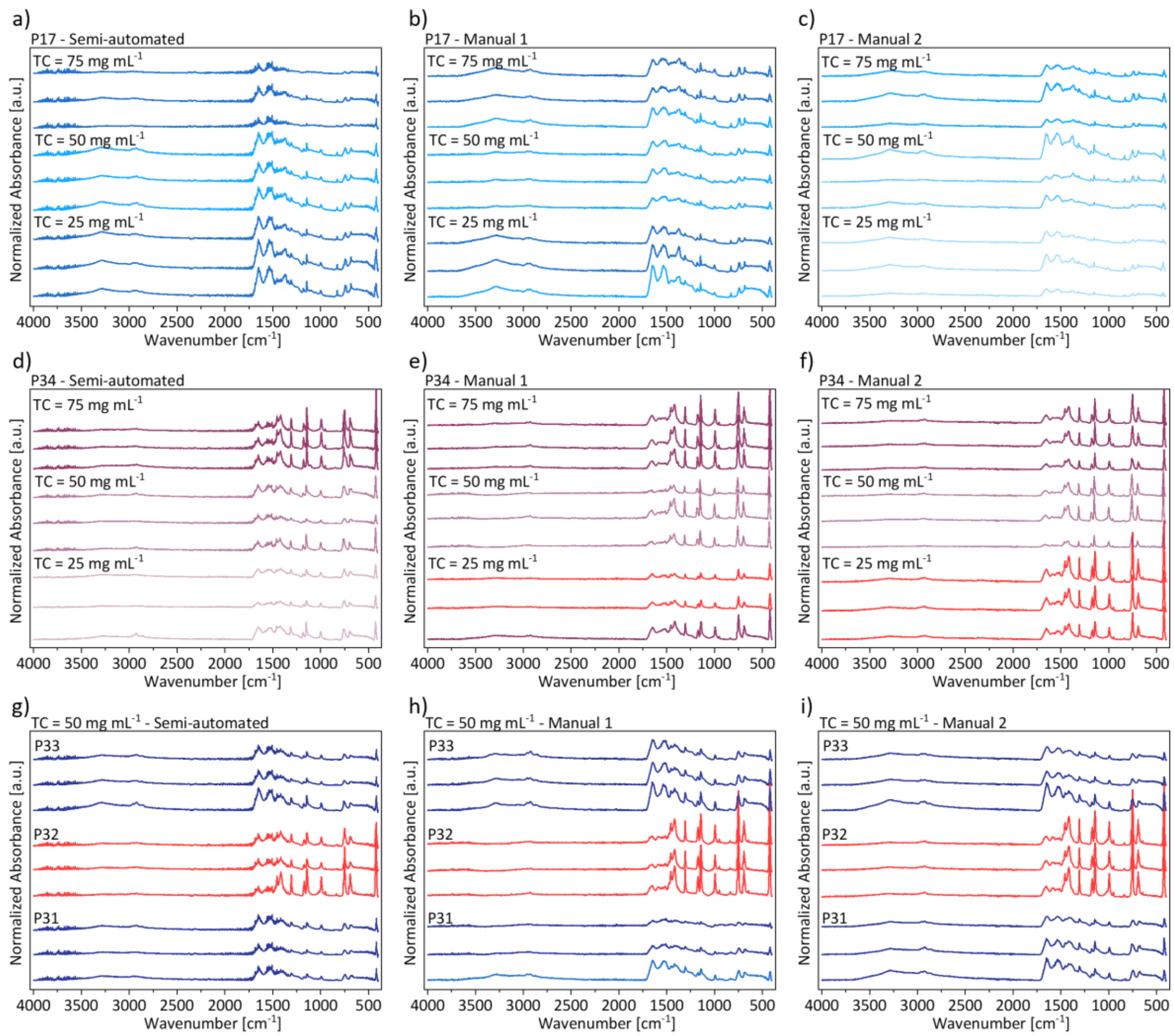

Figure S8. Comparison of triplicate IR spectra obtained using semi-automated, manual 1, and manual 2 workflows. a)–c) P17 at TC = 25, 50, and 75 mg mL^{-1}; d)–f) P34 at TC = 25, 50, and 75 mg mL^{-1}; and g)–i) P31, P32, and P33 at TC = 50 mg mL^{-1}.

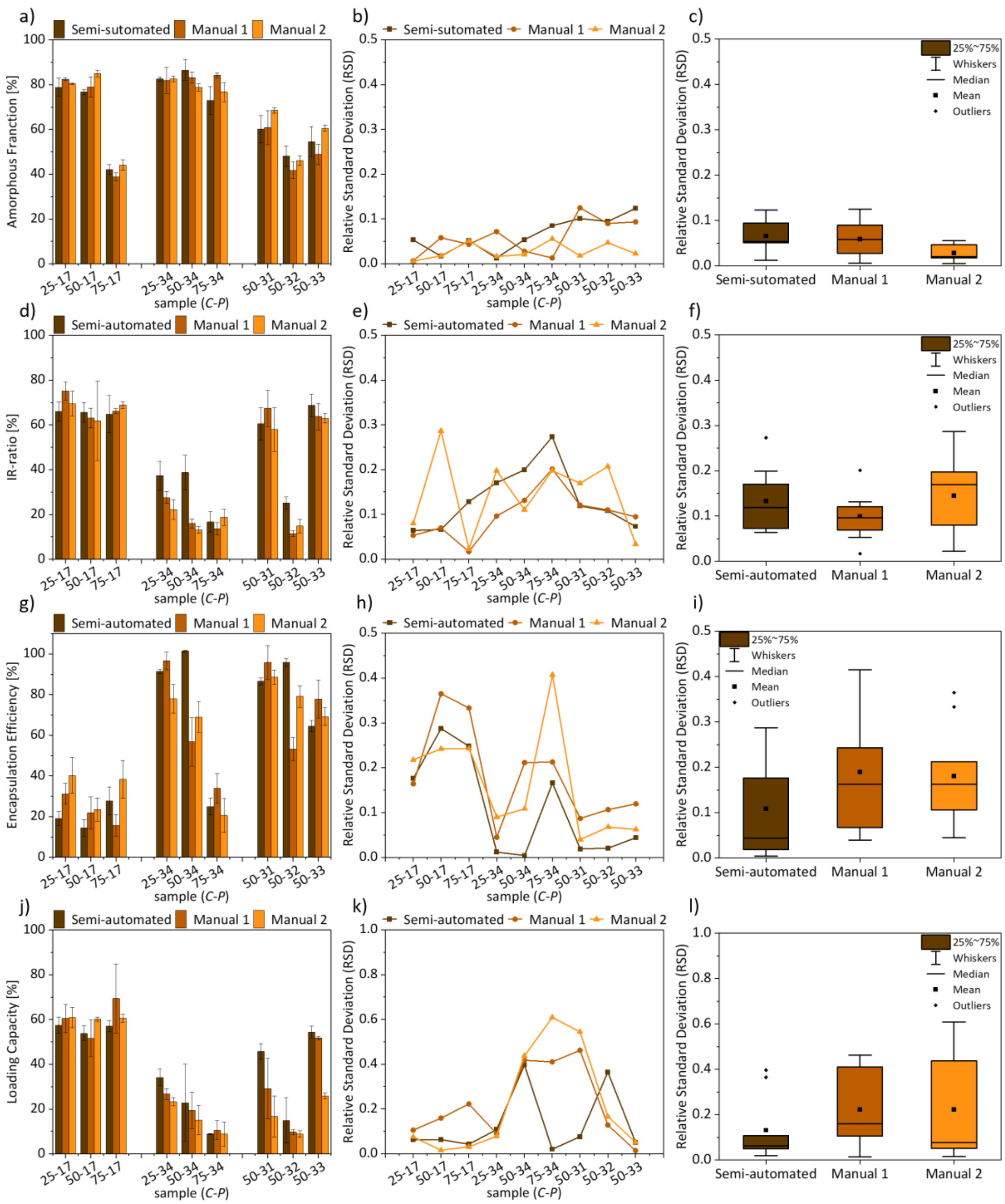

Figure S9. Comparison of triplicate measurements and their relative standard deviations (RSDs) for PXRD-derived amorphous fraction (a-c), IR ratio (d-f), EE% (g-i), and LC% (j–l). a, d, g, and j show the average values with SDs; b, e, h, and k) show the corresponding RSDs as line plots; and c, f, i, and l) show the RSD distributions as box plots. In the box plots, the box represents the

interquartile range (IQR), corresponding to the central 50% of the data; the center line indicates the median, the square marker indicates the mean, and the whiskers extend to the most extreme data points within 1 × IQR from the lower and upper quartiles. SDs represent the standard deviation of triplicate measurements ($n = 3$). We note that EE% and LC% show reduced operator-dependent variability under controlled reagent delivery. In contrast, the RSDs of the PXRD-derived amorphous fraction and IR ratio show no clear differences between manual and controlled reagent delivery. This may be related to the semi-quantitative nature of these two measurements, which are likely less sensitive to small variations between replicates than EE% and LC%, which are determined using quantitative methods (Bradford assay and ICP-OES, respectively).

2 The offline version of ZIF Biocomposite Explorer App

2.1 User guidelines for the offline version of ZIF Biocomposite Explorer App

The offline version of the ZIF Biocomposite Explorer App (ZIF-BEA) is an interactive platform designed for the visualization and exploration of high-throughput ZIF biocomposite datasets. The application enables users to investigate compositional, structural, and performance-related data through layered ternary diagrams and integrated characterization results. Figures and datasets can be exported for further analysis and reporting.

2.1.1 Getting started

The offline version of ZIF Biocomposite Explorer App operates as a standalone application and does not require installation. After extracting the provided ZIP archive, launch the application by opening the corresponding HTML file in a web browser.

2.1.2 Interface overview

The interface consists of four main sections:

Navigation Bar (top): Displays the application title, institutional logos, and export functions. The Download Page PNG button exports the entire current interface as a high-resolution image.

Control Panel (left): Provides options for dataset selection, visualization settings, and data filtering.

Diagram Display Panel (center): Displays layered ternary diagrams corresponding to different total precursor concentrations.

Results Presentation Panel (right): Shows detailed information and characterization data for the selected sample.

Users can search for sample positions and select the dataset (TD-H_2O or TD-EtOH), total precursor concentration (all concentrations or a specific concentration), and visualization mode. Samples can be visualized according to crystal phase, EE%, LC%, IR ratio, or amorphous fraction.

2.1.3 Data Exploration and Visualization

The control panel allows users to filter samples according to formulation parameters (M, L, and BSA) and characterization metrics. The "Reset Filters" button restores the default settings. In "Phase" mode, samples are color-coded according to their assigned phase (amorphous, **ZIF-C**, **sod**, **dia**, **U13**, **U12**, or mixed). Mixed-phase samples are represented by segmented colors indicating the relative proportion of each phase. In EE%, LC%, IR ratio, amorphous fraction and framework ratio modes, samples are colored according to the corresponding numerical values.

The toolbar above the ternary diagrams provides additional display controls. Users can show or hide sample numbers, concentration labels, axis labels, and axis values, and adjust their display size. The "Rotate" and "Pan" functions control mouse interaction, while the "Layer Spacing" slider adjusts the separation between ternary-diagram layers. Predefined viewing directions can be

accessed using the “X”, “Y”, “Z”, and “L” buttons. Zooming can be performed using the mouse wheel or the “+” and “−” controls. Each TD layer represents a specific total precursor concentration and contains 36 samples. Selecting a sample displays its detailed information in the Results Presentation Panel. Double-clicking an empty region of the diagram clears the current selection.

2.1.4 Results Presentation and Data Export

The Results Presentation Panel displays the phase composition, formulation parameters, EE%, LC%, IR ratio, amorphous fraction and framework ratio of the selected sample. For mixed-phase samples, the average phase distribution obtained from triplicate syntheses is reported. A summary table compares the same sample across different total precursor concentrations, facilitating the evaluation of concentration-dependent trends. PXRD patterns and ATR-IR spectra associated with the selected sample are displayed in the lower section of the panel. Figures can be exported in PNG format using the corresponding download buttons, as shown in **Figure S11** and **Figure S12**. Normalized PXRD and ATR-IR datasets can be downloaded as CSV files for further analysis. The “Reset” function restores the default spectral view, while the “+” and “−” controls allow zooming of the displayed spectra.

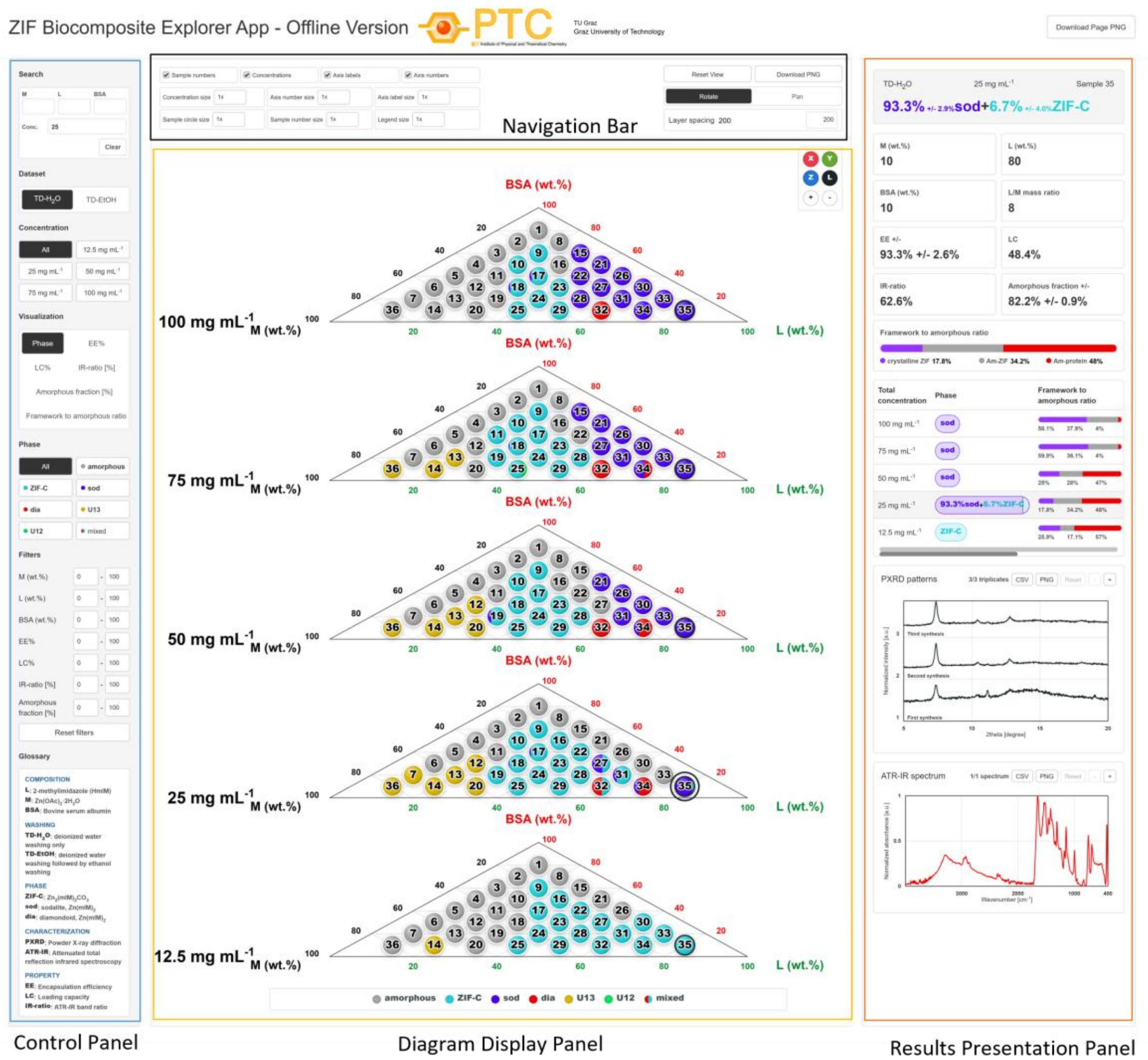

Figure S10. Interface of the offline version of the ZIF Biocomposite Explorer App. The app displays ZIF biocomposite synthesis data in a stacked ternary-diagram format, where different layers correspond to different TCs. The left control panel allows selection of washing procedures, concentration, visualization, and filters. The central panel shows the stacked ternary diagrams, while the right panel provides detailed information for the selected sample, including composition, phase, EE%, LC%, PXRD patterns, and ATR-IR spectrum.

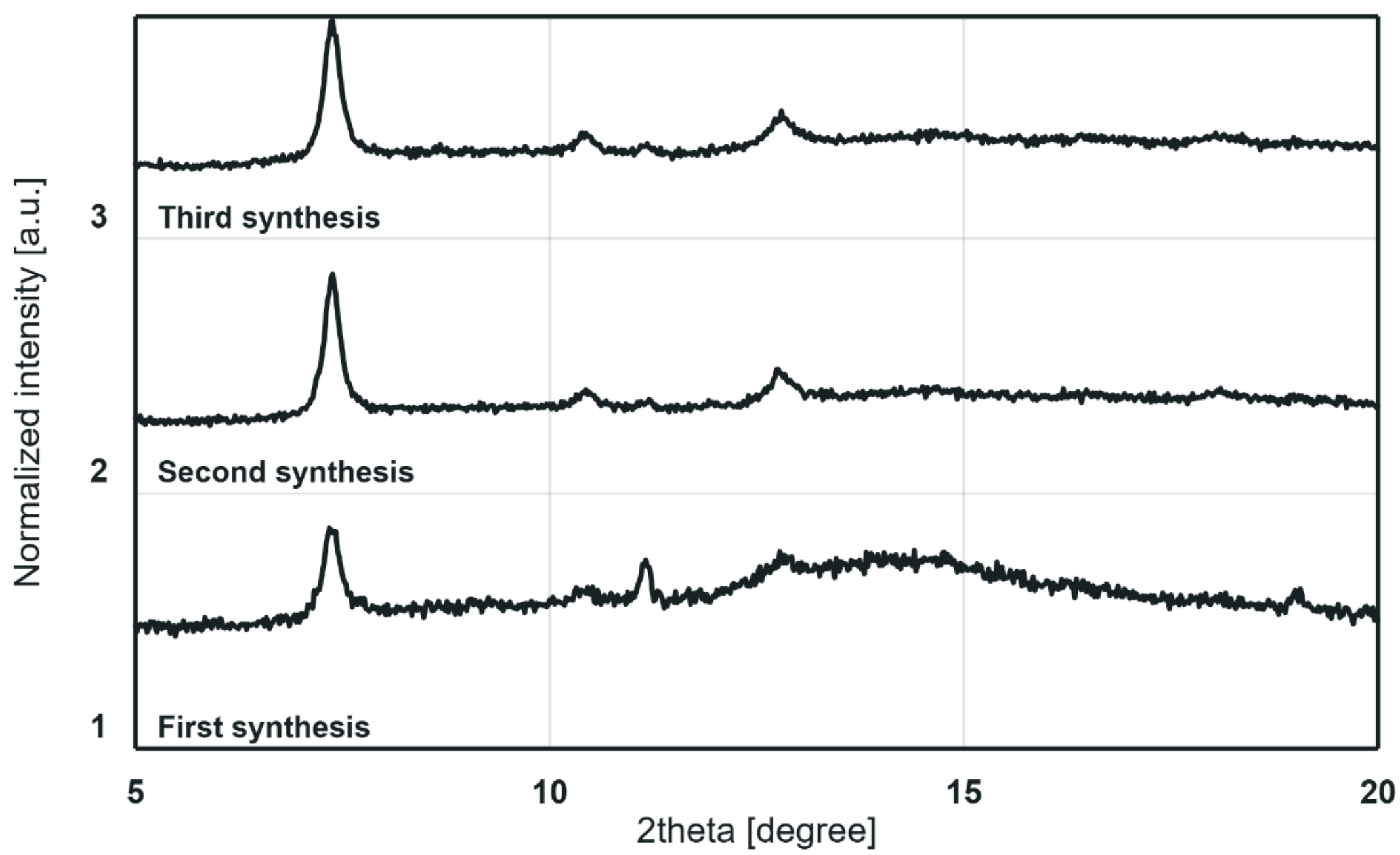

Figure S11. PXRD patterns displayed in the Results Presentation Panel of the offline ZIF Biocomposite Explorer App for sample 35 prepared at the TD-H_2O when TC = 25 mg mL^{-1}. The three patterns correspond to triplicate syntheses.

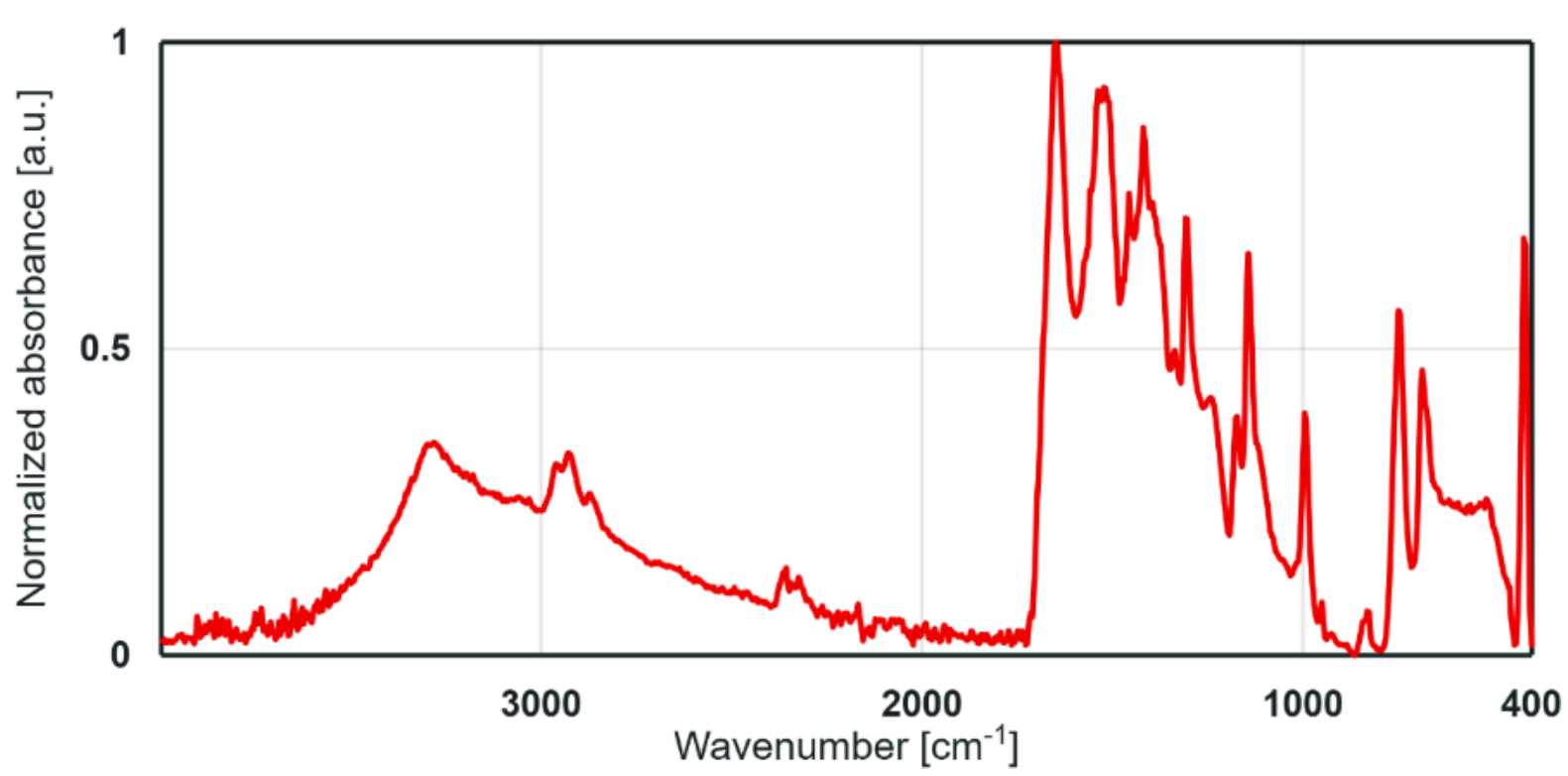

Figure S12. ATR-IR spectrum displayed in the Results Presentation Panel of the offline ZIF Biocomposite Explorer App for sample 35 prepared at the TD-H_2O when TC = 25 mg mL^{-1}.

2.2 Development of the offline ZIF Biocomposite Explorer App

The offline ZIF Biocomposite Explorer App was developed as an interactive platform for the visualization and exploration of ZIF biocomposite datasets. All phase assignments, EE%, LC%, IR ratios, amorphous fractions, framework ratio, PXRD patterns, and ATR-IR spectra displayed in the application originate from experimentally acquired data. Raw experimental results were curated, processed, and organized using Microsoft Excel and Origin, and were stored as Excel spreadsheets and TXT files.

Phase assignment was performed using the ZIF Phase Analysis platform developed at Graz University of Technology (https://rapps.tugraz.at/apps/porousbiotech/ZIFphaseanalysis/)[3]. The processed experimental data were subsequently consolidated into structured Excel datasets and converted into JavaScript data files (data/*.js), which serve as the data source for the interactive ternary diagrams and sample information panels implemented in the offline ZIF Biocomposite Explorer App. The application incorporates the open-source JavaScript library html2canvas v1.4.1 (vendor/html2canvas.min.js) for page-level PNG export. This library is distributed under the MIT License, and additional packages present in the local dependency tree are transitive dependencies of html2canvas that support text layout, Unicode segmentation, and binary encoding utilities.

Table S12. Open-source software used in the offline ZIF biocomposite explorer.

Project	Version	License	Role in this work	Notes
html2canvas[5]	1.4.1	MIT	Runtime page-to-canvas rendering for full-page PNG export	Bundled locally as vendor/html2canvas.min.js. Used for the "Download Page PNG" function.
css-line-break[6]	2.1.0	MIT	Transitive dependency of html2canvas	Supports CSS/text line breaking behavior during DOM rendering.
text-segmentation[7]	1.0.3	MIT	Transitive dependency of html2canvas	Supports Unicode grapheme segmentation during text rendering.
Utrie[8]	1.0.2	MIT	Transitive dependency	Provides Unicode trie data structures used by text segmentation and line-breaking utilities.
base64-arraybuffer[9]	1.0.2	MIT	Transitive dependency	Provides base64 encoding and decoding utilities for ArrayBuffer data.

3 PXRD patterns and analysis

3.1 PXRD patterns

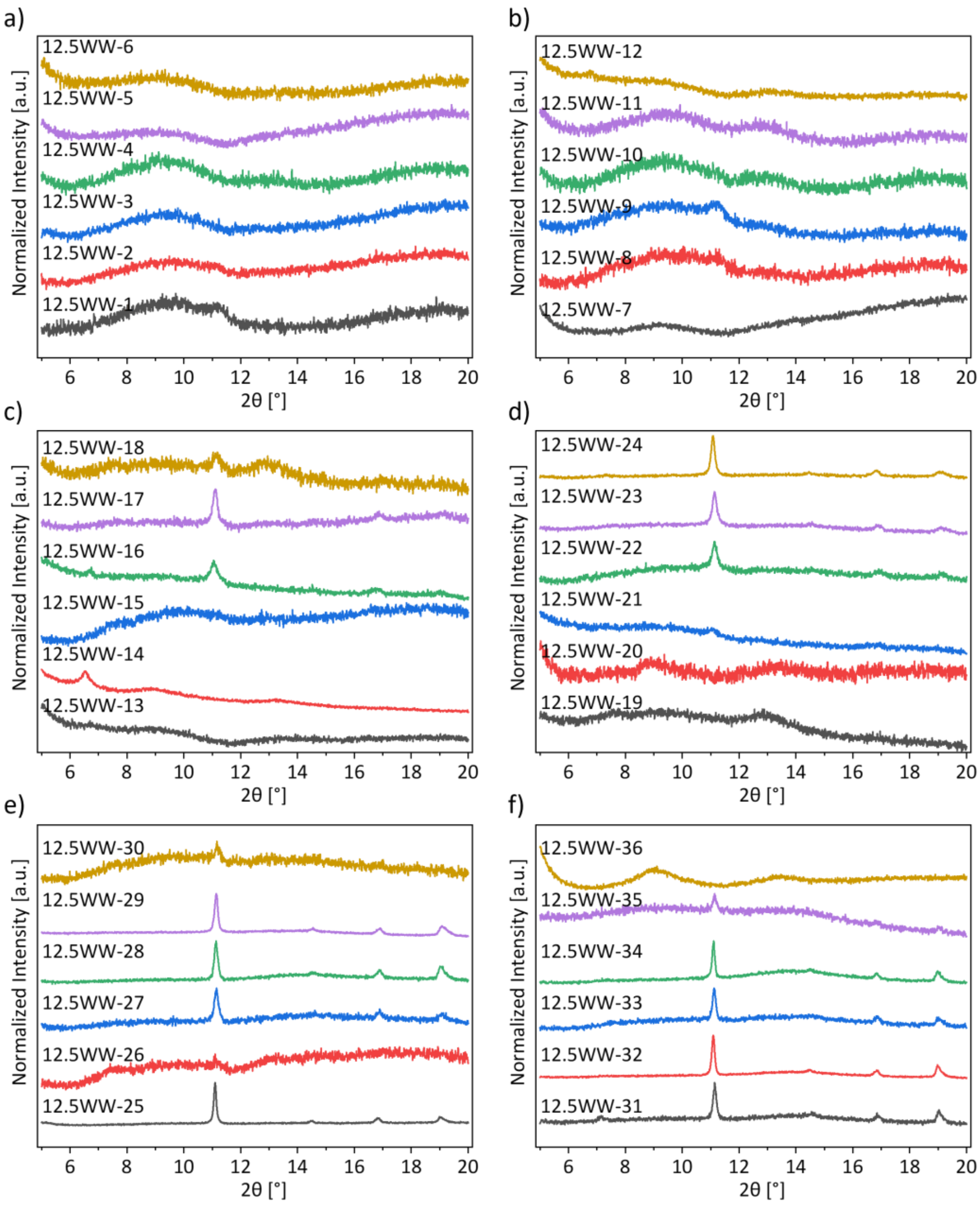

Figure S13. PXRD patterns of WW (TD-H_2O) system prepared at TC = 12.5 mg mL^{-1}.

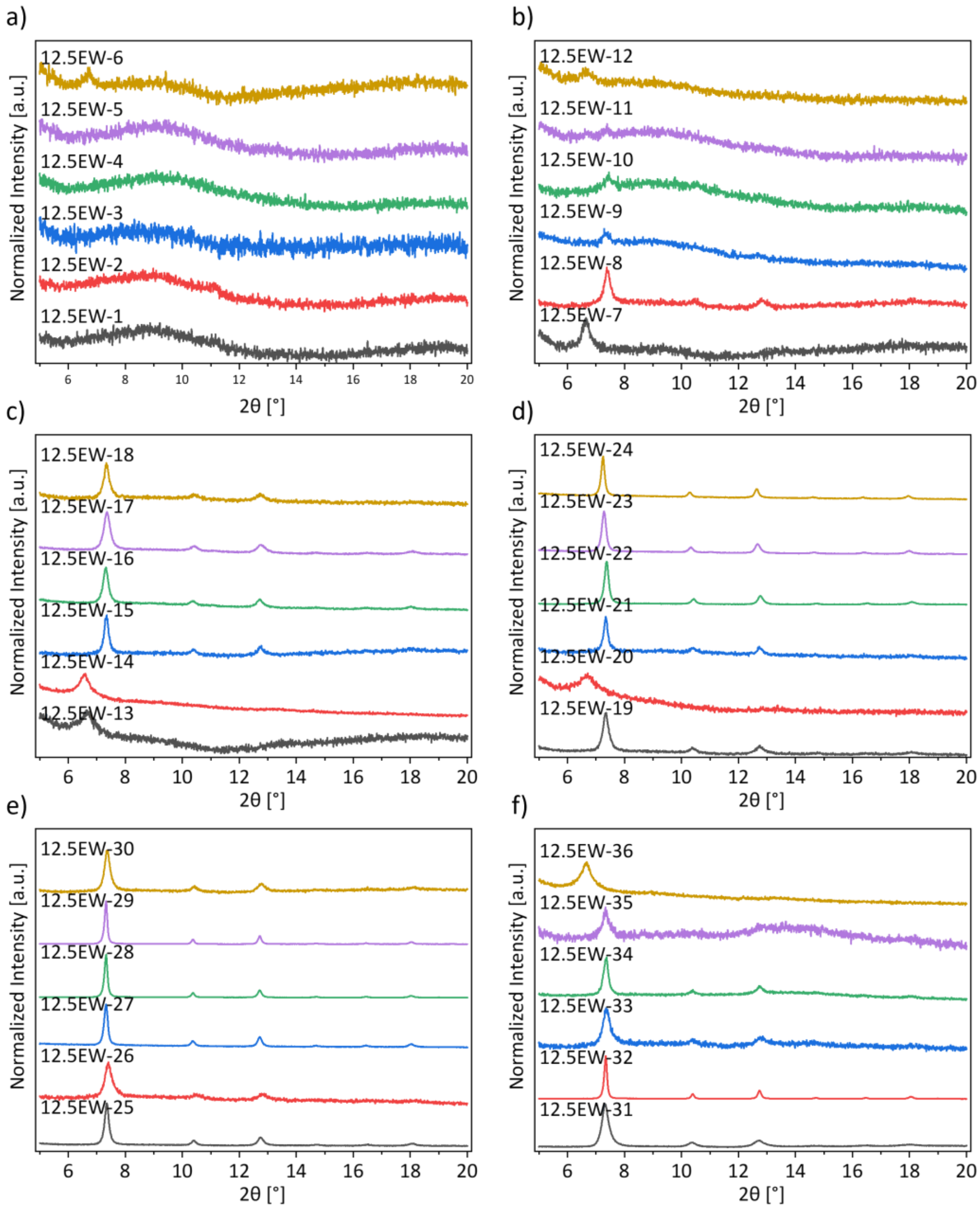

Figure S14. PXRD patterns of EW system prepared at TC = 12.5 mg mL^{-1}.

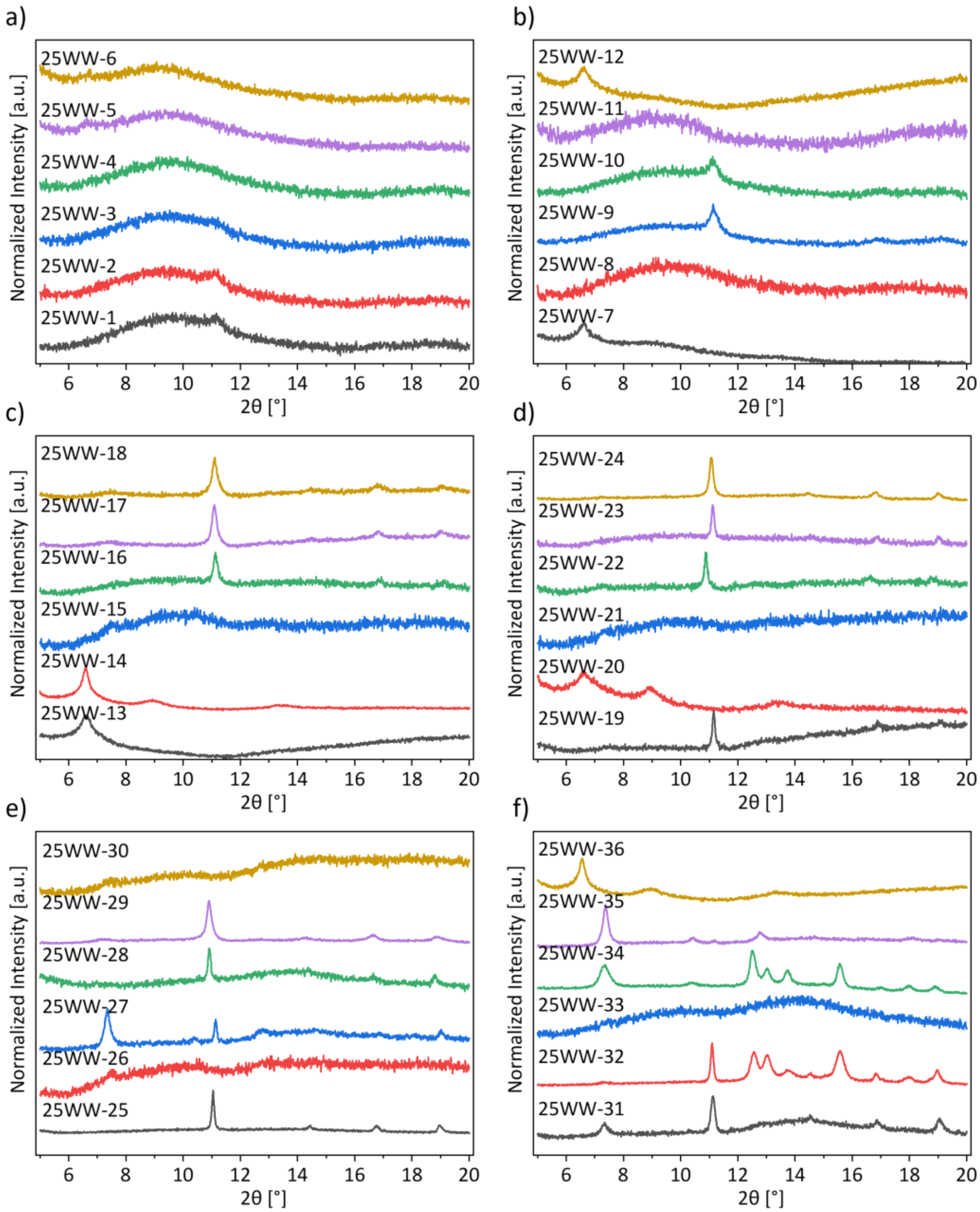

Figure S15. PXRD patterns of WW (TD-H_2O) system prepared at TC = 25 mg mL^{-1}.

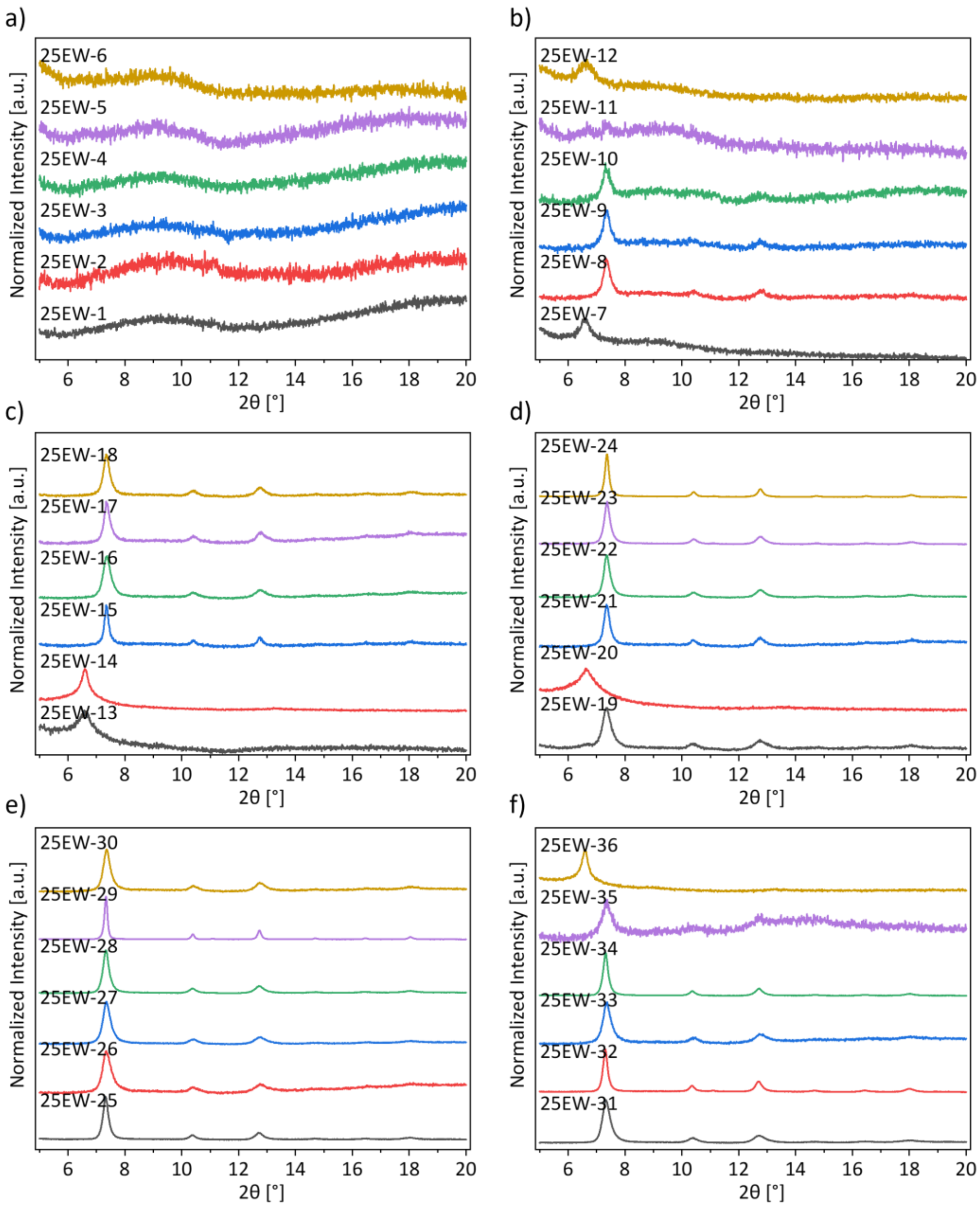

Figure S16. PXRD patterns of EW (TD-EtOH) system prepared at TC = 25 mg mL^{-1}.

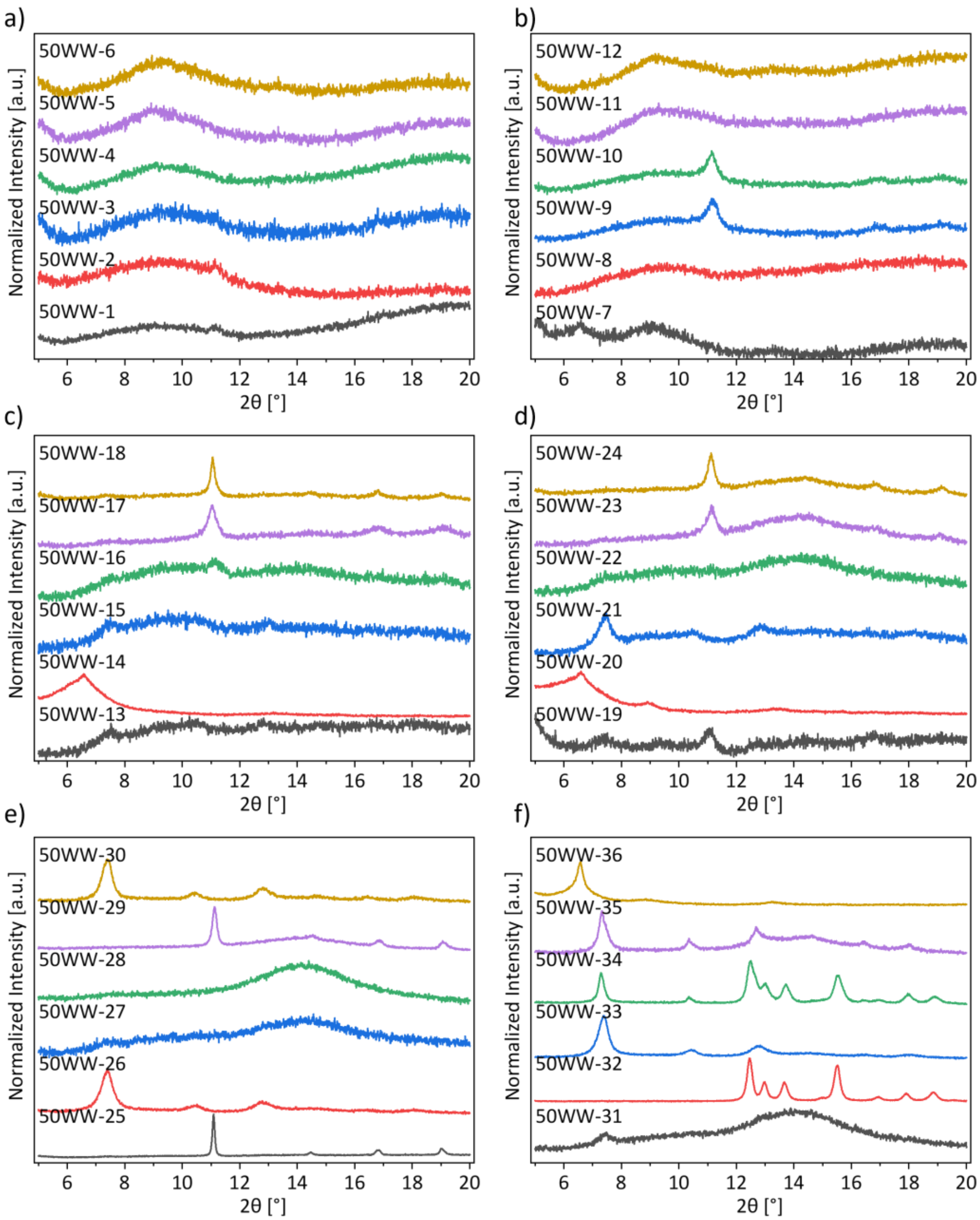

Figure S17. PXRD patterns of WW (TD-H_2O) system prepared at TC = 50 mg mL^{-1}.

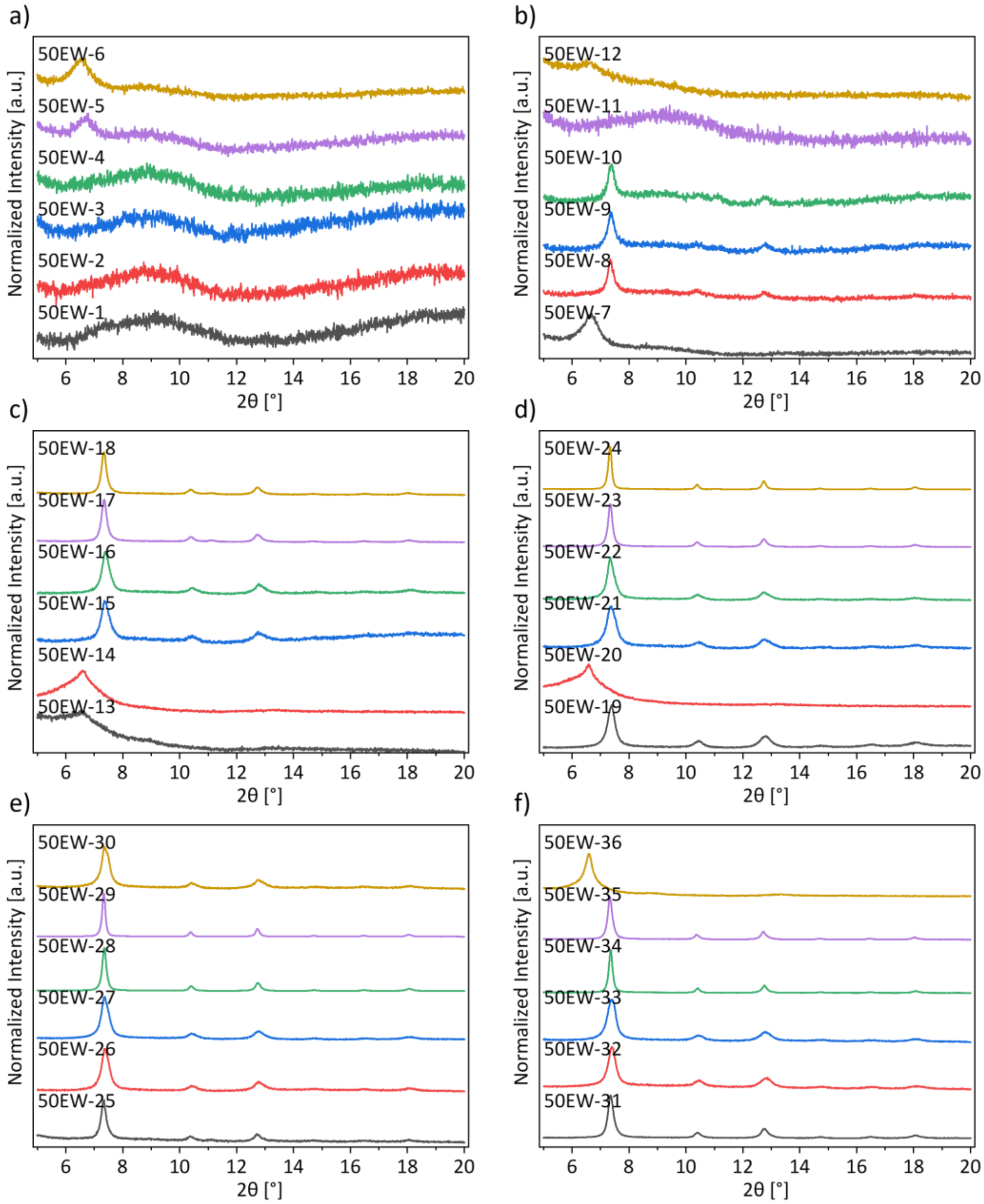

Figure S18. PXRD patterns of EW (TD-EtOH) system prepared at TC = 50 mg mL^{-1}.

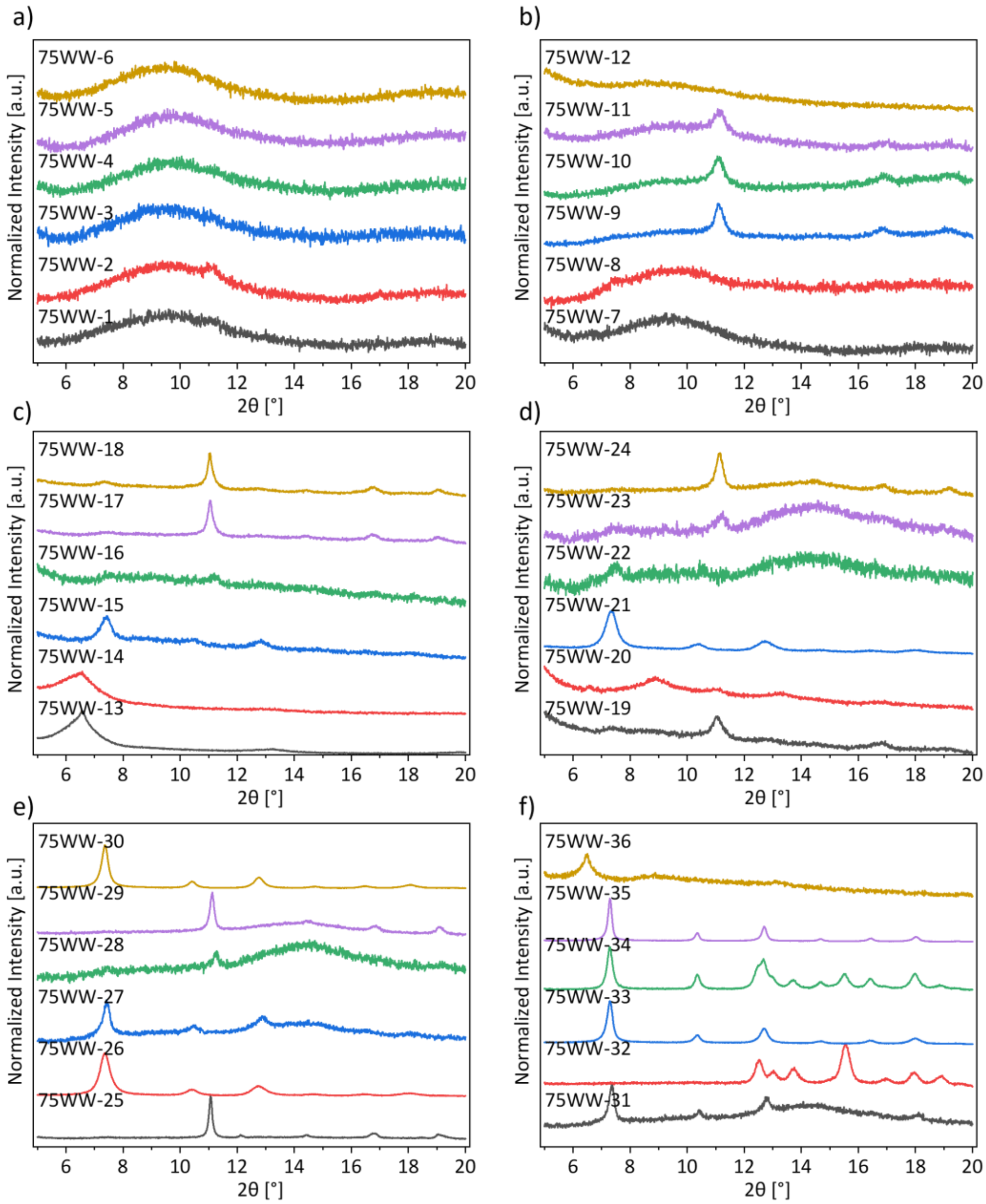

Figure S19. PXRD patterns of WW (TD-H_2O) system prepared at TC = 75 mg mL^{-1}.

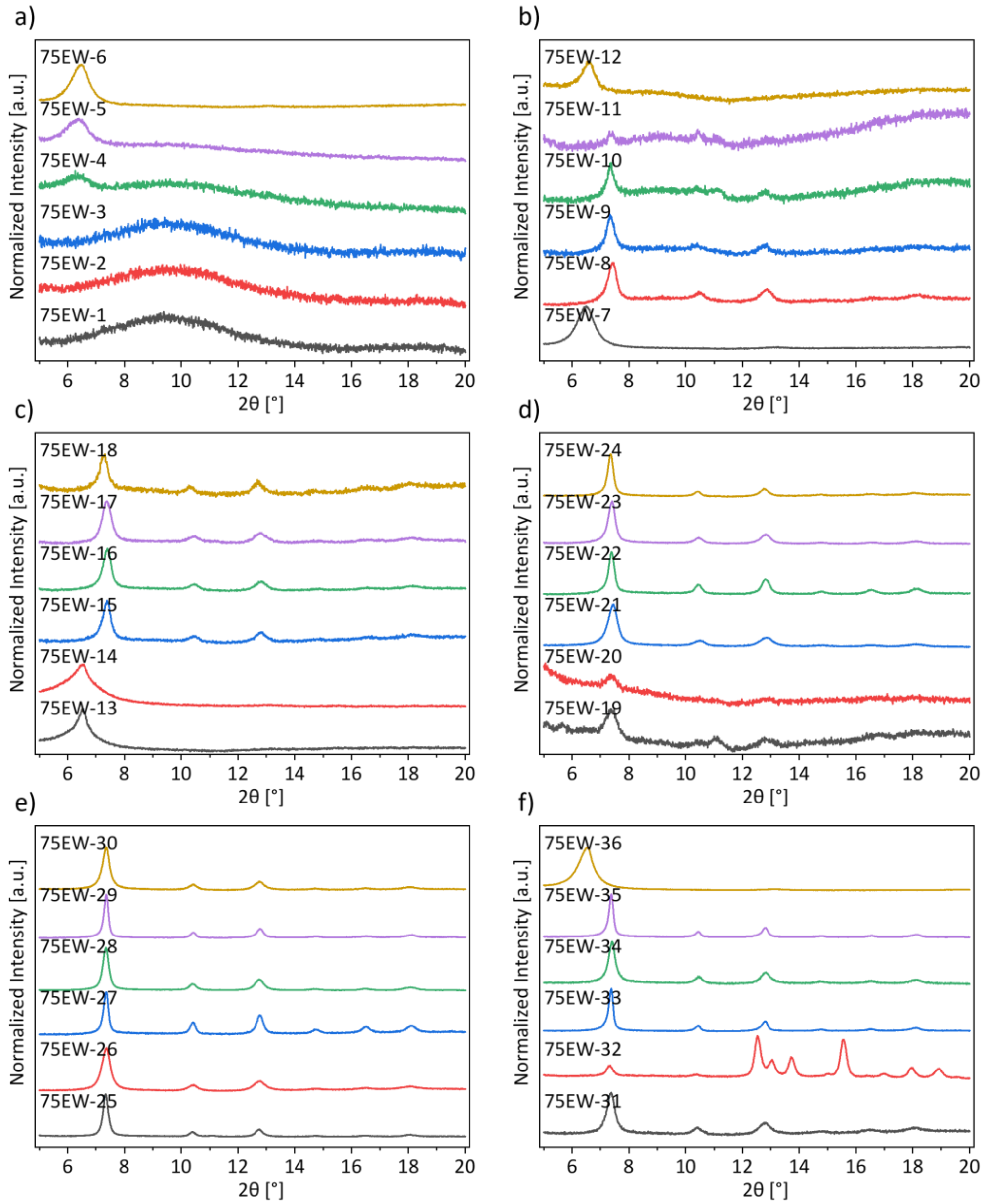

Figure S20. PXRD patterns of EW (TD-EtOH) system prepared at TC = 75 mg mL^{-1}.

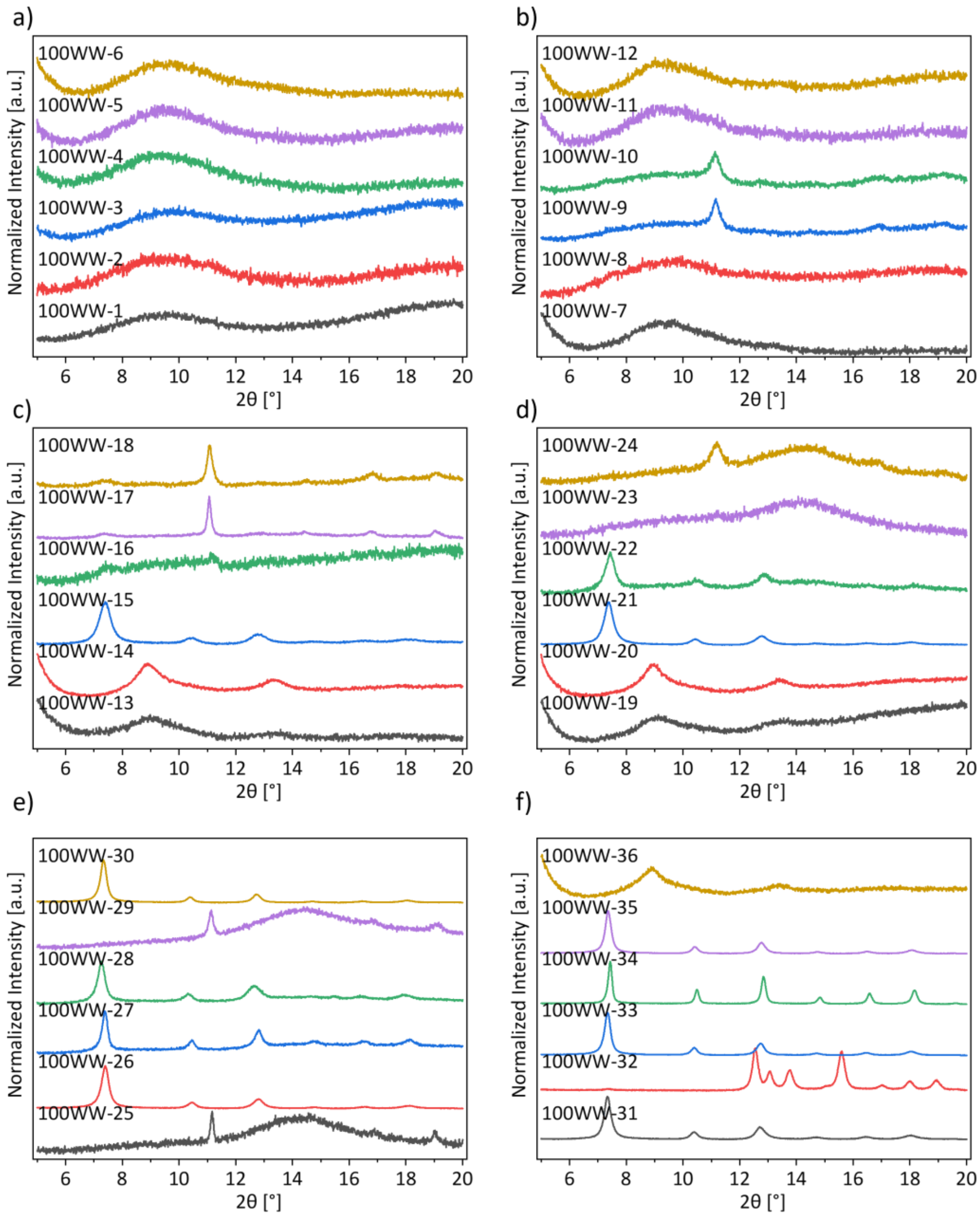

Figure S21. PXRD patterns of WW (TD-H_2O) system prepared at TC = 100 mg mL^{-1}.

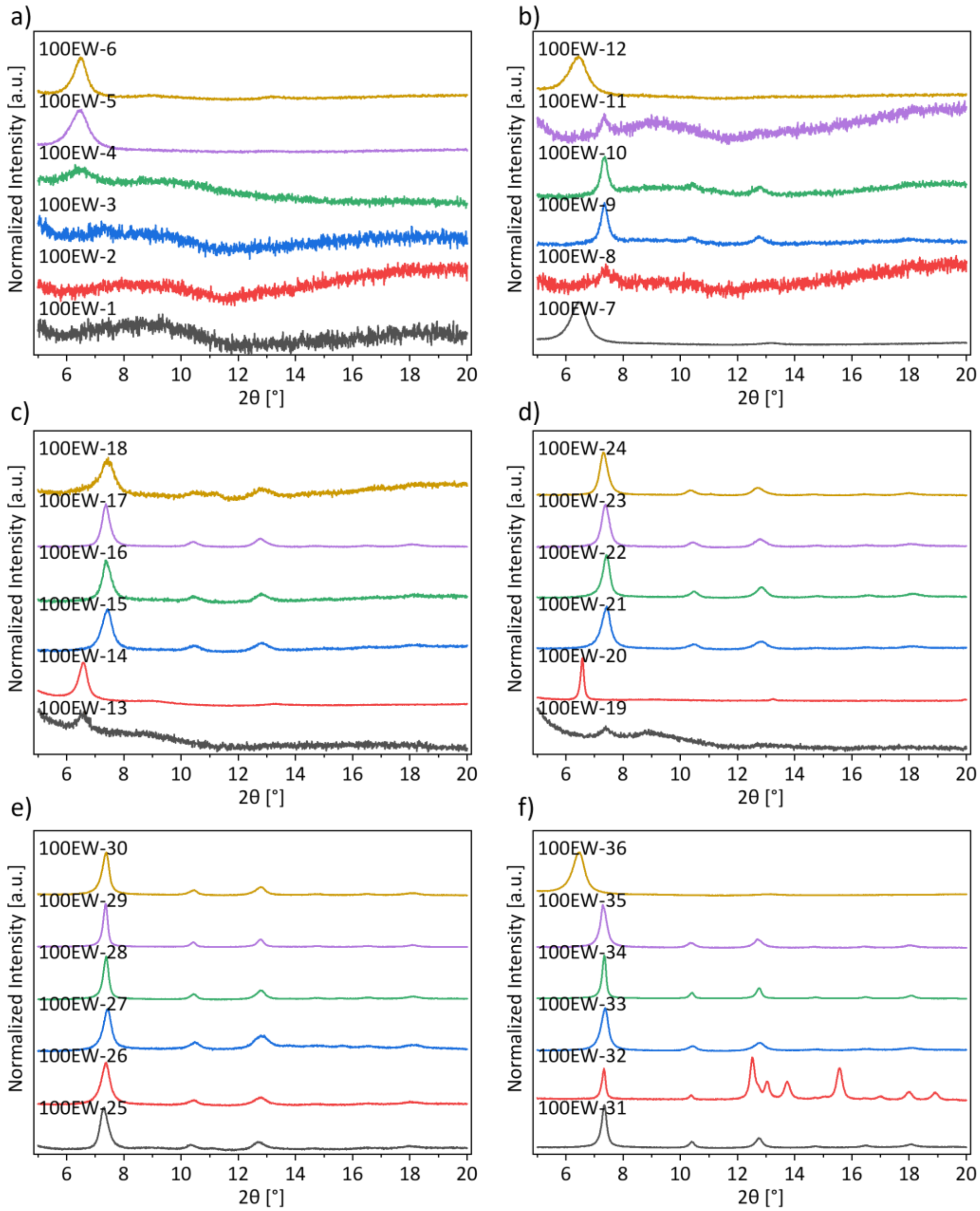

Figure S22. PXRD patterns of EW (TD-EtOH) system prepared at TC = 100 mg mL^{-1}.

3.2 TDs of phases

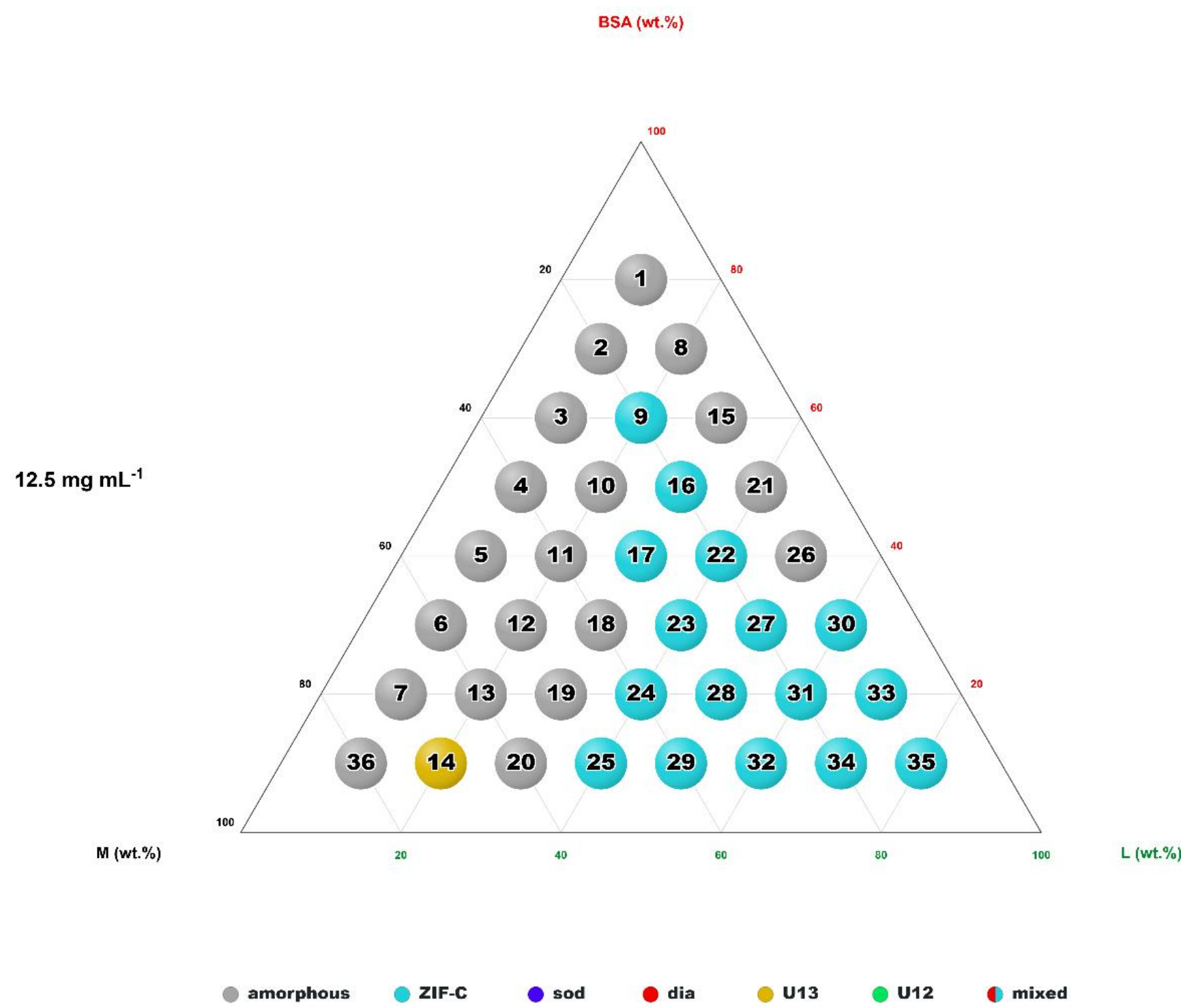

Figure S23. TD-phase for the WW (TD-H_2O) system prepared at TC = 12.5 mg mL^{-1}.

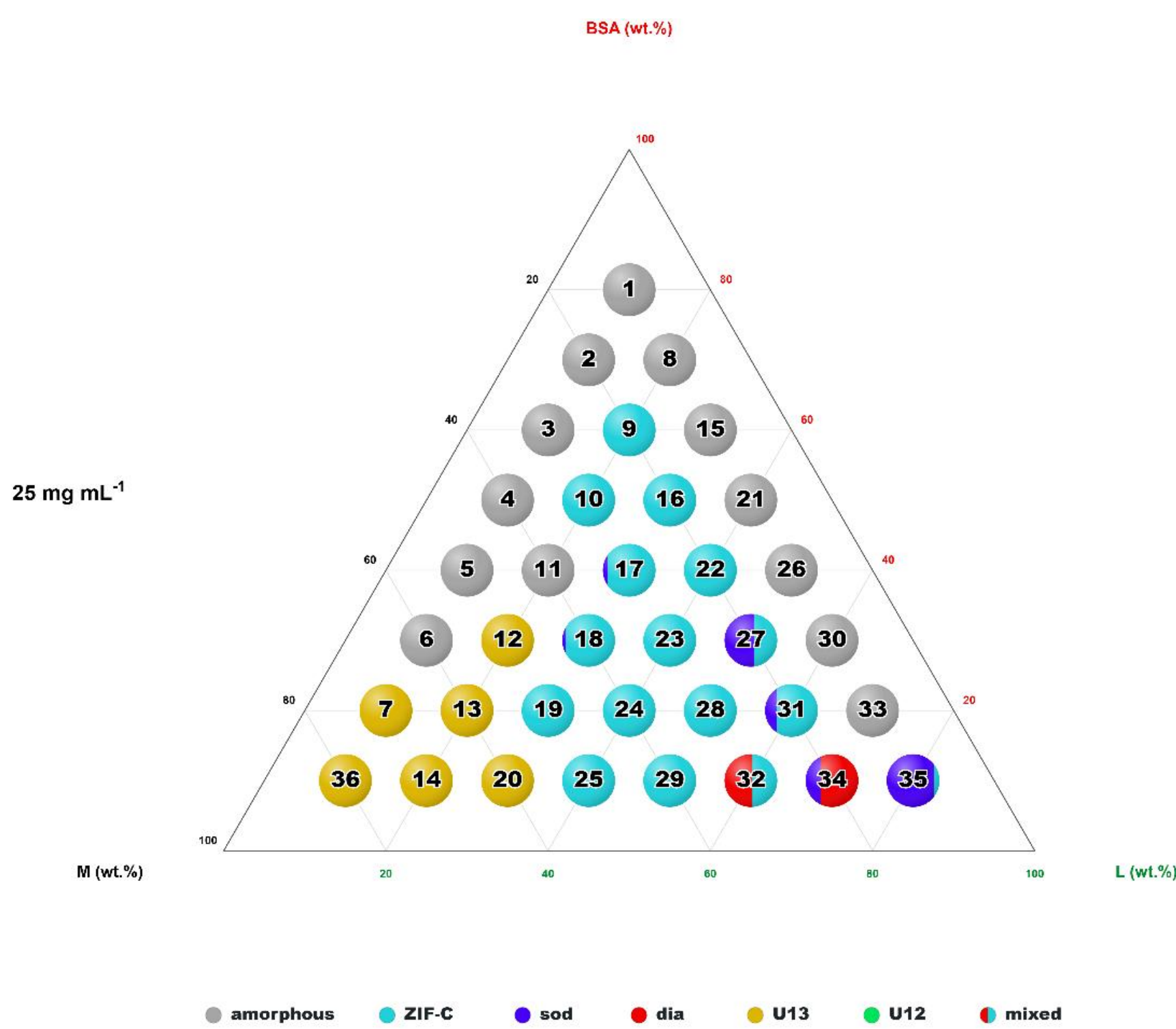

Figure S24. TD-phase for the WW (TD-H_2O) system prepared at TC = 25 mg mL^{-1}.

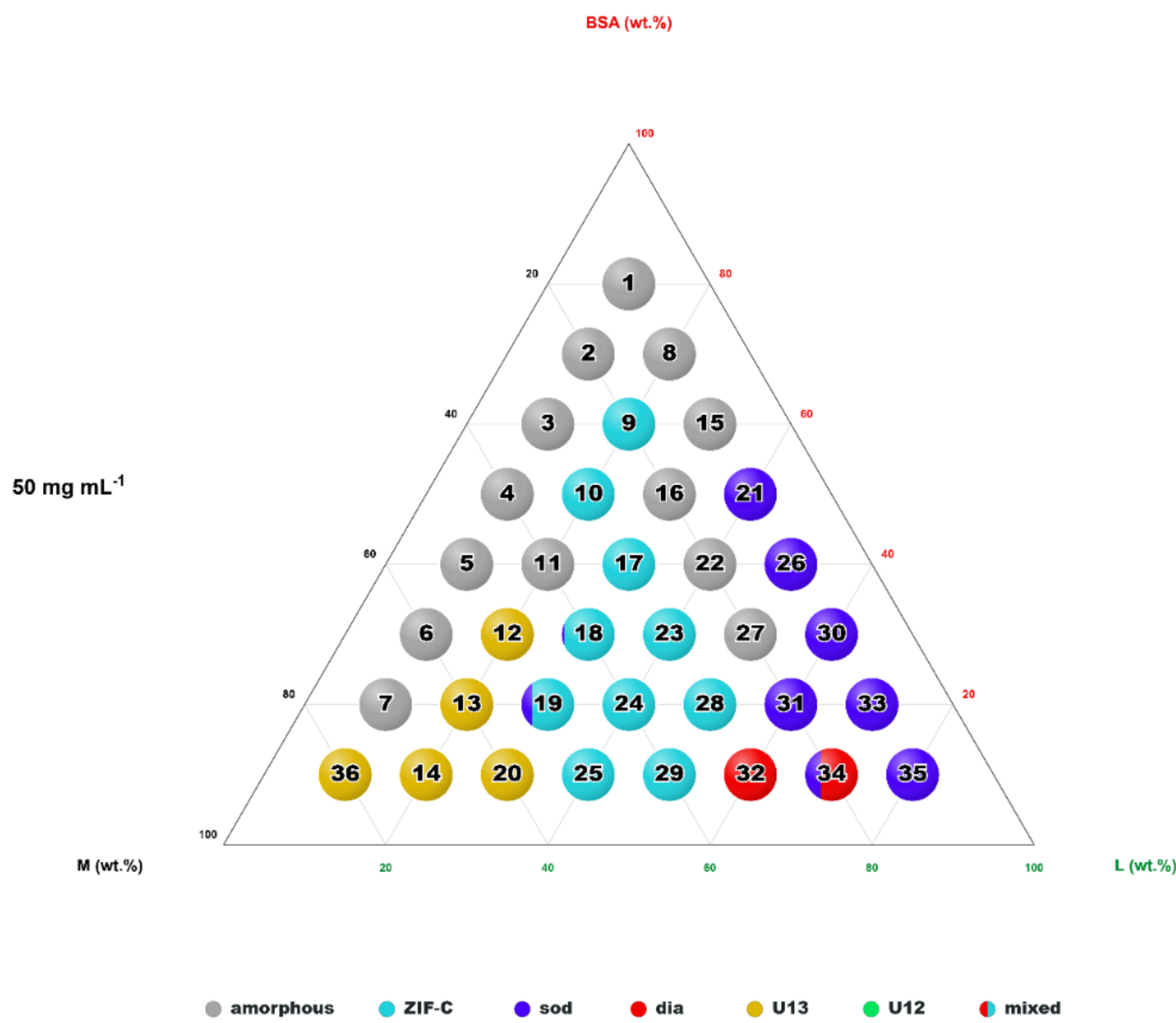

Figure S25. TD-phase for the WW (TD-H_2O) system prepared at TC = 50 mg mL^{-1}.

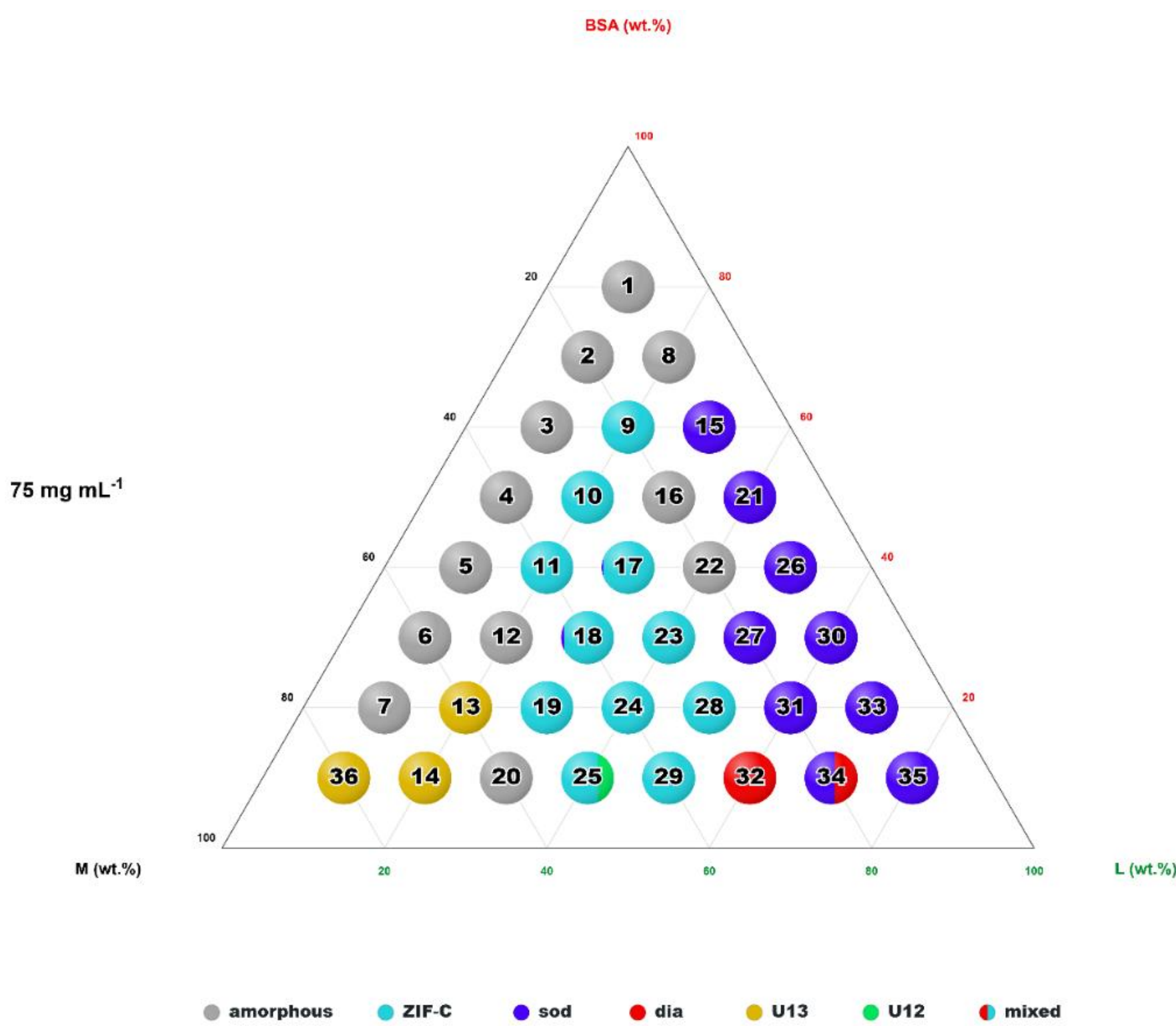

Figure S26. TD-phase for the WW (TD-H_2O) system prepared at TC = 75 mg mL^{-1}.

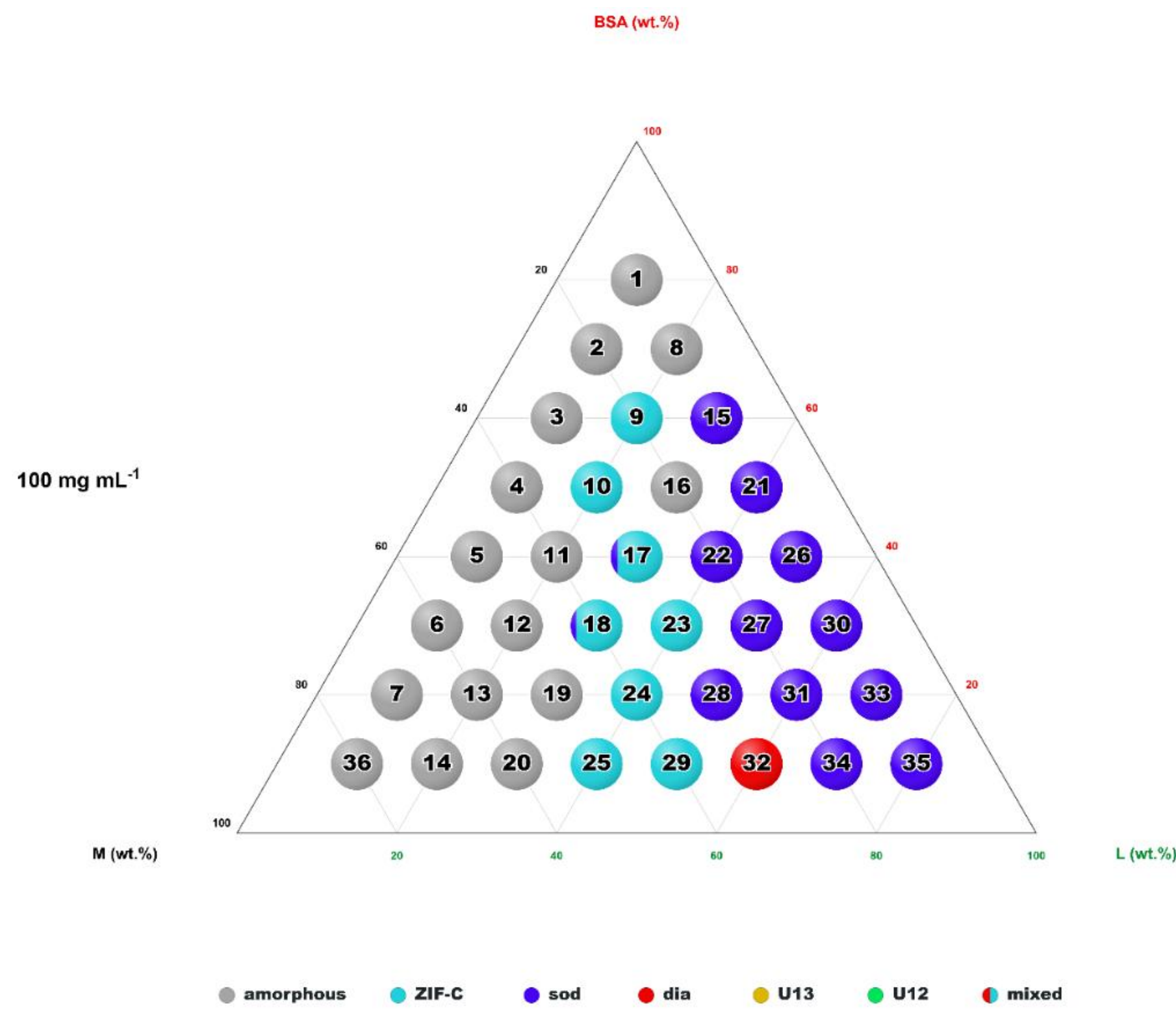

Figure S27. TD-phase for the WW (TD-H_2O) system prepared at TC = 100 mg mL^{-1}.

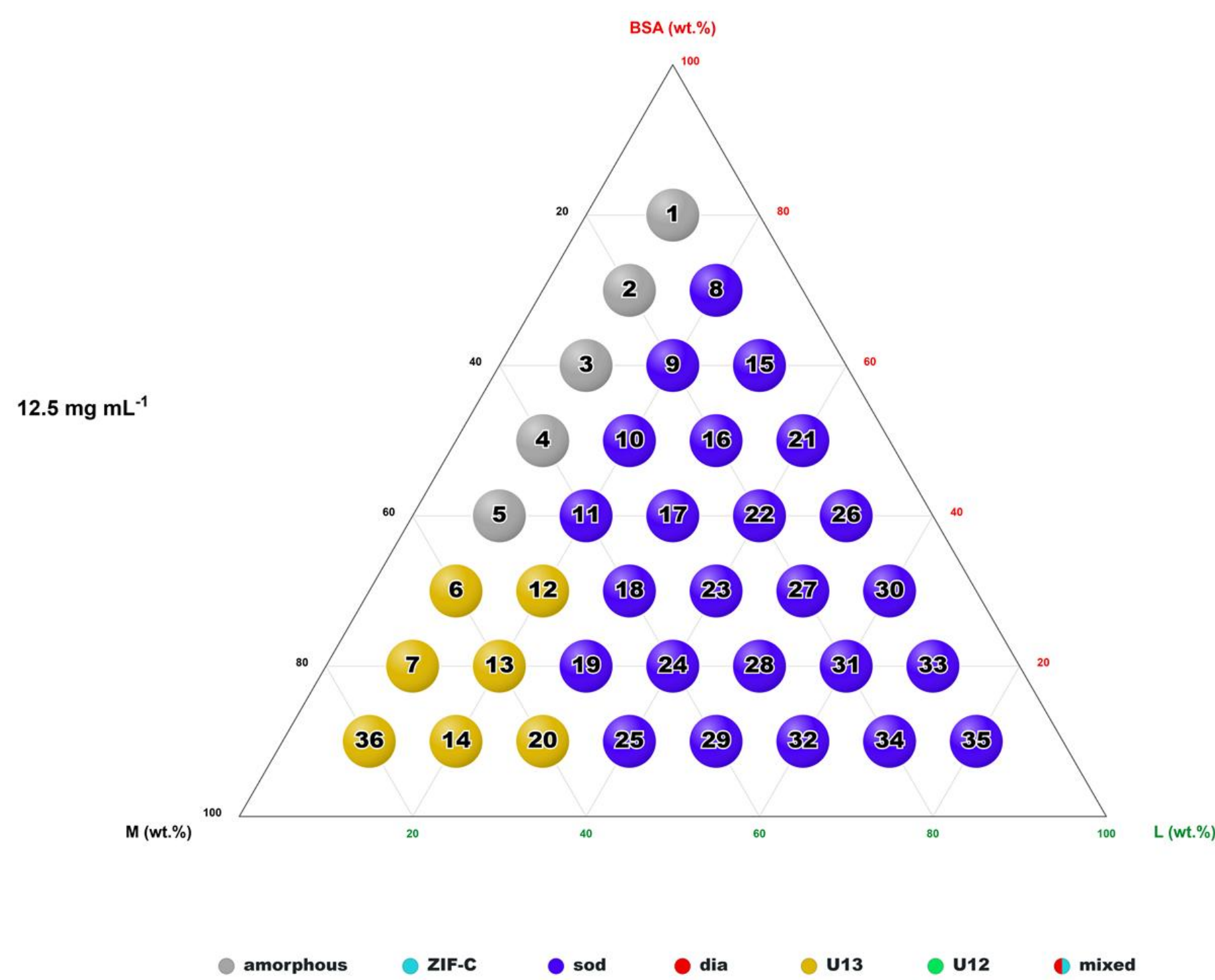

Figure S28. TD-phase for the EW (TD-EtOH) system prepared at TC = 12.5 mg mL^{-1}.

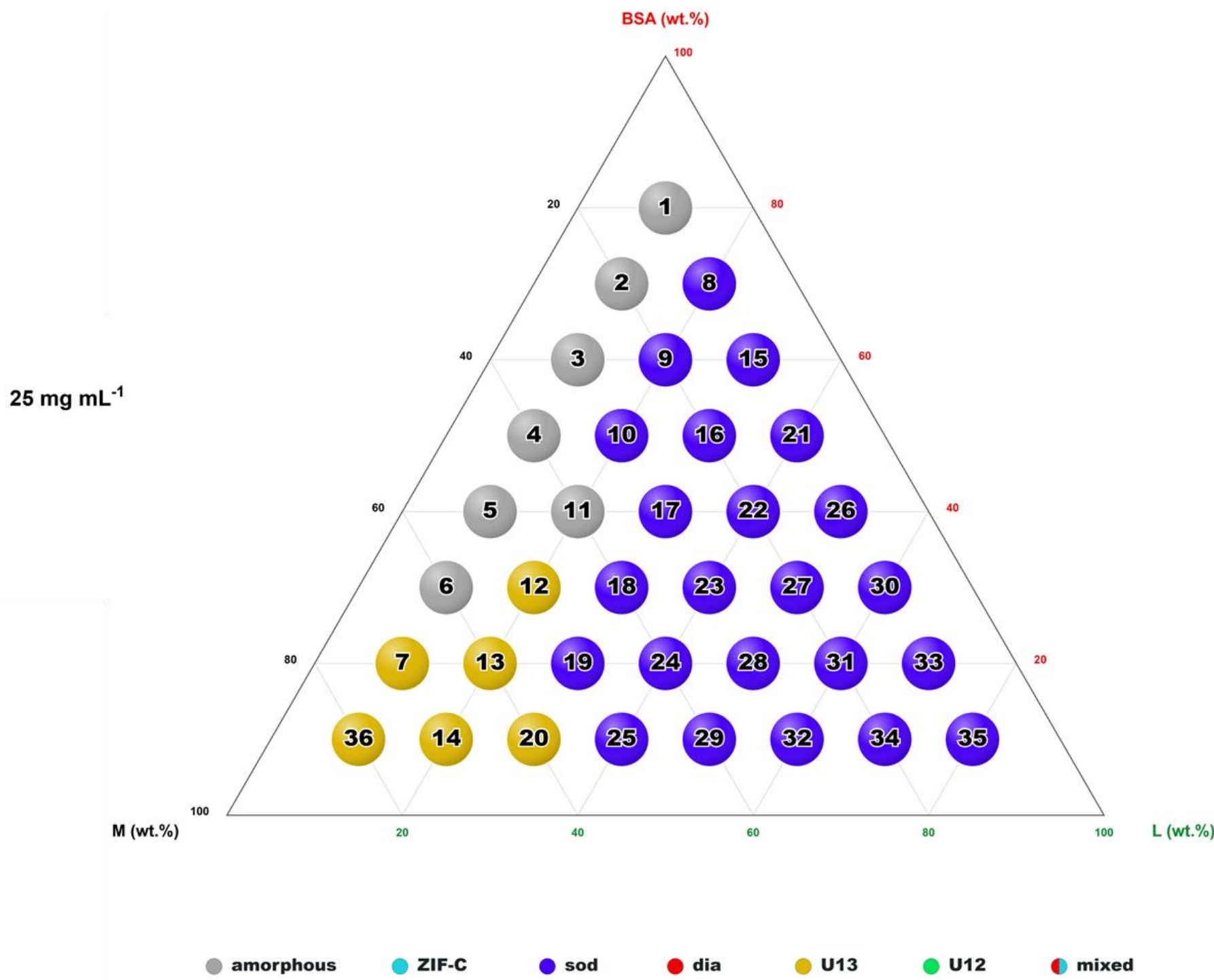

Figure S29. TD-phase for the EW (TD-EtOH) system prepared at TC = 25 mg mL^{-1}.

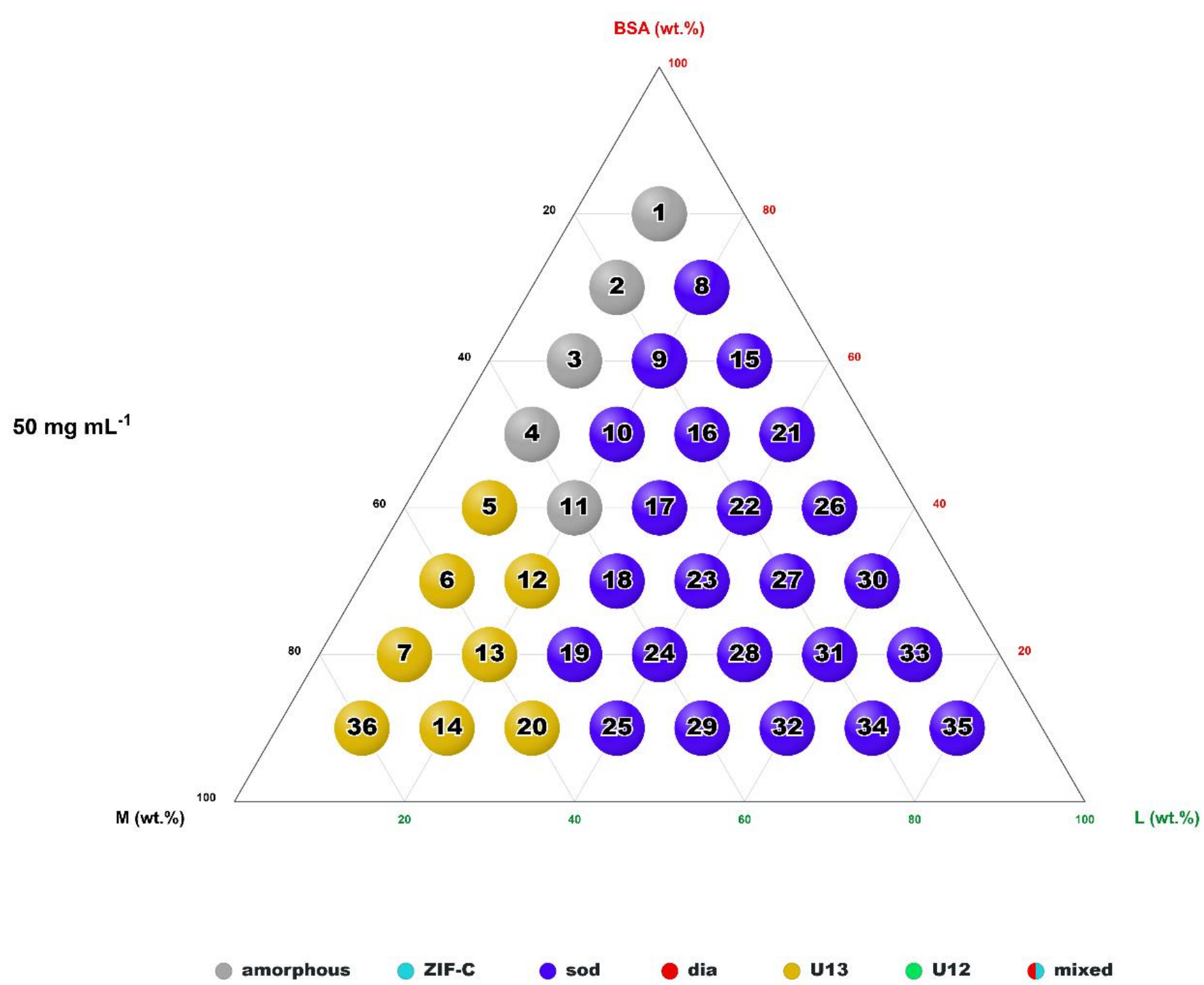

Figure S30. TD-phase for the EW (TD-EtOH) system prepared at TC = 50 mg mL^{-1}.

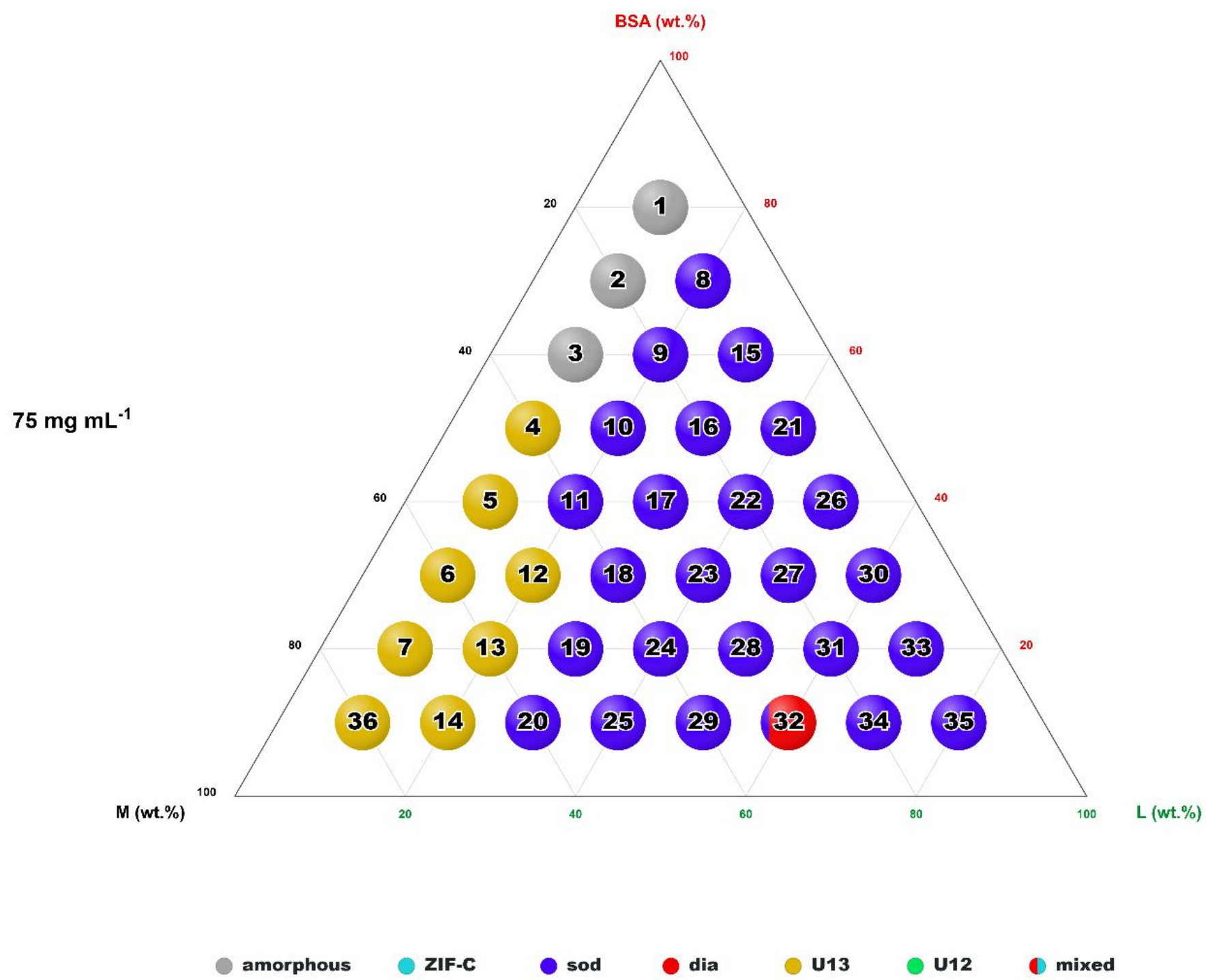

Figure S31. TD-phase for the EW (TD-EtOH) system prepared at TC = 75 mg mL^{-1}.

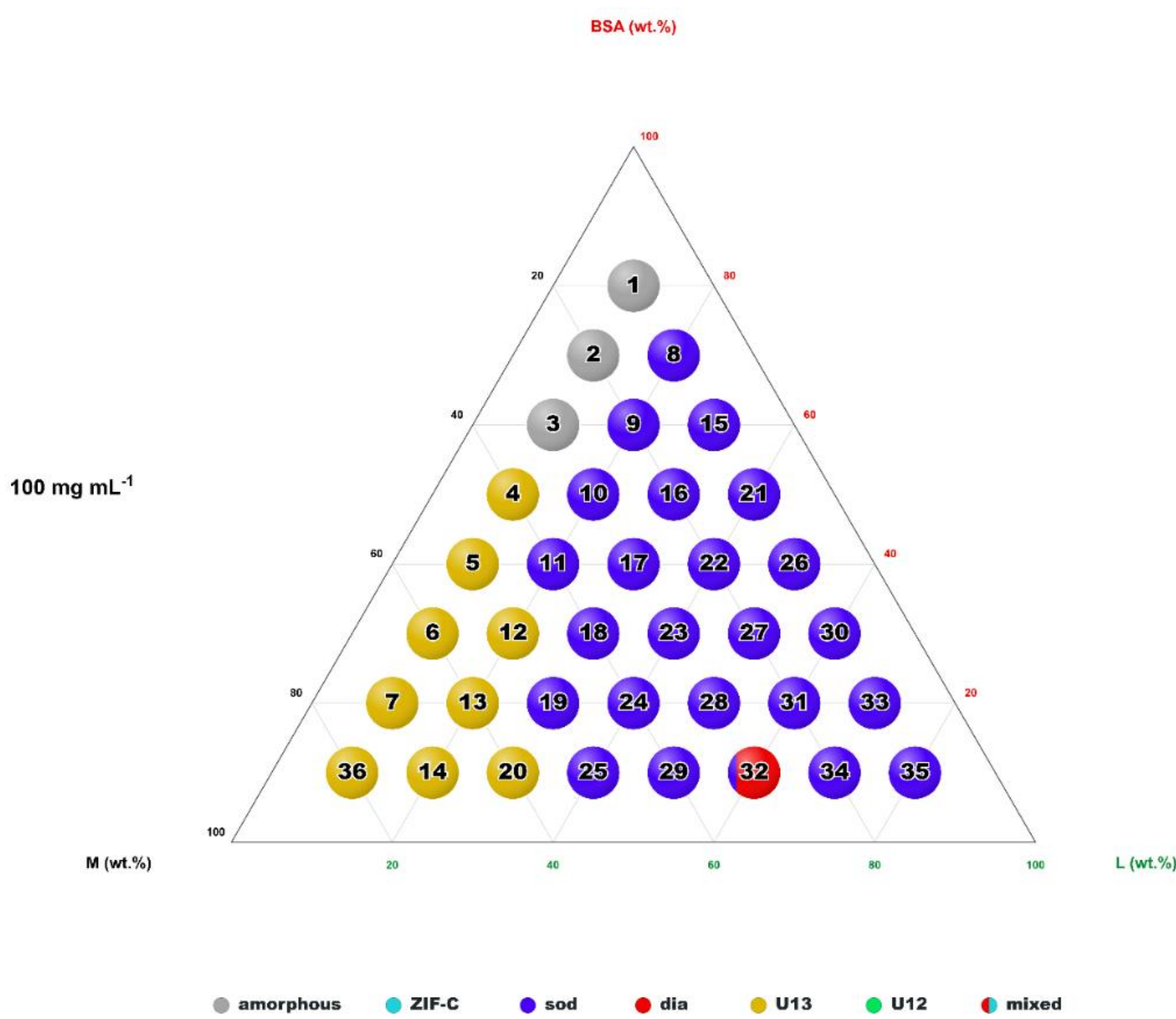

Figure S32. TD-phase for the EW (TD-EtOH) system prepared at TC = 100 mg mL^{-1}.

3.3 Phase summary

Table S13. Phase distribution and composition of ZIF biocomposites in the WW (TD-H_2O) system. Values are given as the number of samples and percentages in parentheses.

WW	**Am**	**Am-ZIF**	**ZIF-C**	**sod**	**dia**	**U13**	Mixed	total
12.5WW	6 (16.7%)	13 (36.1%)	16 (44.4%)	0 (0%)	0 (0%)	1 (2.8%)	0 (0%)	36
25WW	6 (16.7%)	7 (19.4%)	10 (27.8%)	0 (0%)	0 (0%)	6 (16.7%)	7 (19.4%)	36
50WW	7 (19.4%)	6 (16.7%)	8 (22.2%)	6 (16.7%)	1 (2.8%)	5 (13.9%)	3 (8.3%)	36
75WW	8 (22.2%)	4 (11.1%)	8 (22.2%)	8 (22.2%)	1 (2.8%)	3 (8.3%)	4 (11.1%)	36
100WW	9 (25.0%)	7 (19.4%)	6 (16.7%)	11 (30.6%)	1 (2.8%)	0 (0%)	2 (5.6%)	36
Total	36 (20.0%)	37 (20.6%)	48 (26.7%)	25 (13.9%)	3 (1.7%)	15 (8.3%)	16 (8.9%)	180

Table S14. Phase distribution and composition of ZIF biocomposites in the EW (TD-EtOH) system. Values are given as the number of samples and percentages in parentheses.

EW	**sod**	**U13**	**Am**	**sod+dia**	total
12.5EW	24 (66.7%)	7 (19.4%)	5 (13.9%)	0 (0%)	36
25EW	23 (63.9%)	6 (16.7%)	7 (19.4%)	0 (0%)	36
50EW	23 (63.9%)	8 (22.2%)	5 (13.9%)	0 (0%)	36
75EW	24 (66.7%)	8 (22.2%)	3 (8.3%)	1 (2.8%)	36
100EW	23 (63.9%)	9 (25.0%)	3 (8.3%)	1 (2.8%)	36
Total	117 (65.0%)	38 (21.1%)	23 (12.8%)	2 (1.1%)	180

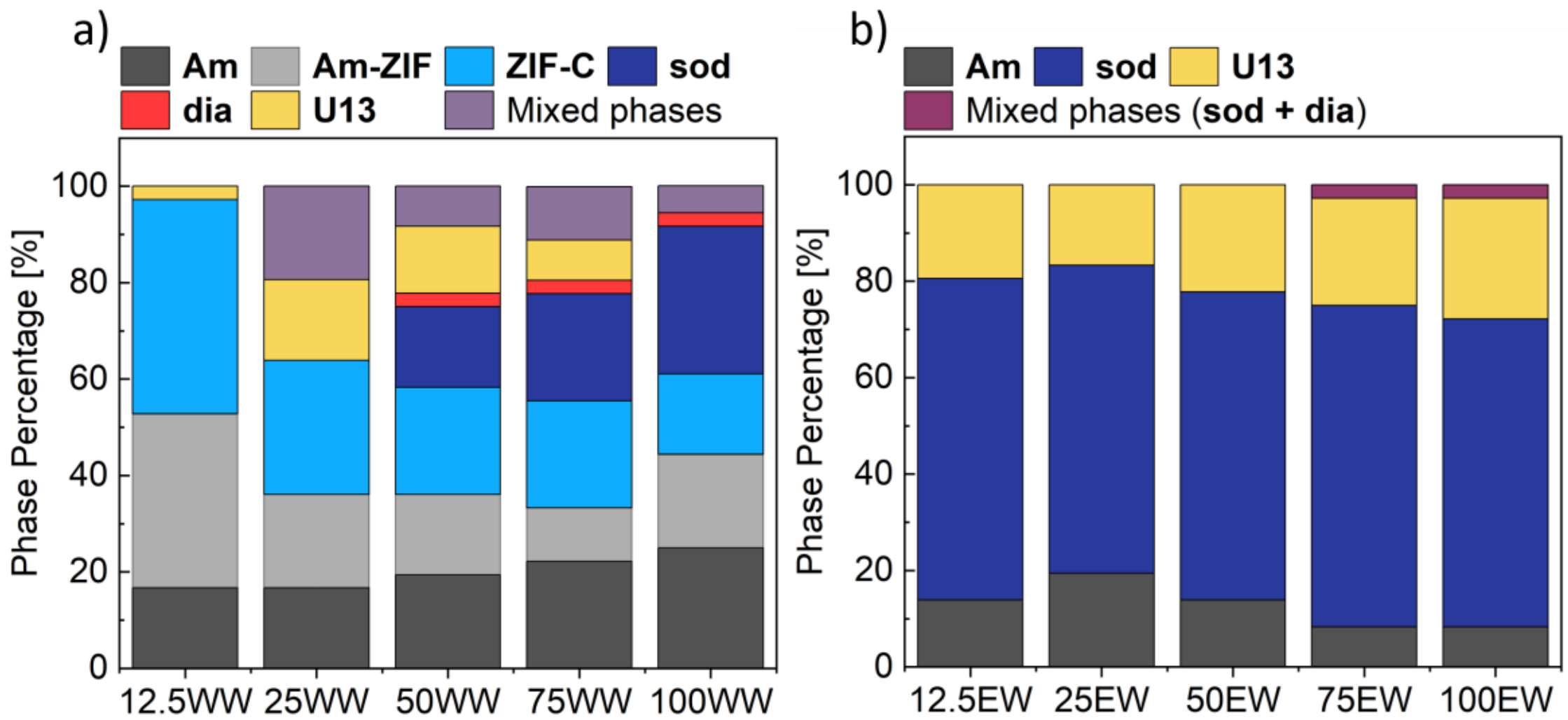

Figure S33. Stacked bar charts showing the phase distribution and composition of ZIF biocomposites in the WW (TD-H_2O) and EW (TD-EtOH) systems.

4 Quantification of the protein immobilization

4.1 TDs of EE%

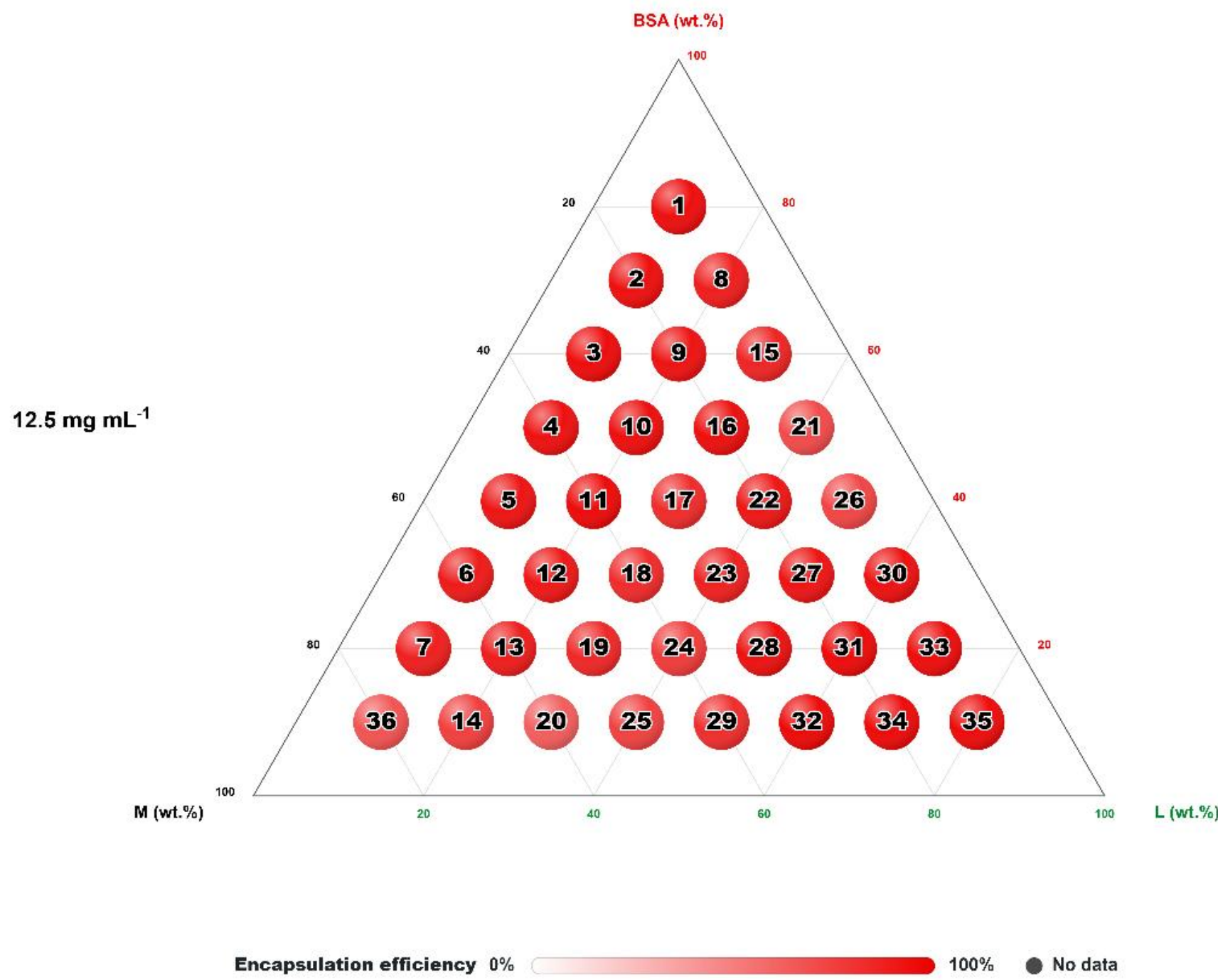

Figure S34. TD-EE% for the WW (TD-H_2O) system prepared at TC = 12.5 mg mL^{-1}.

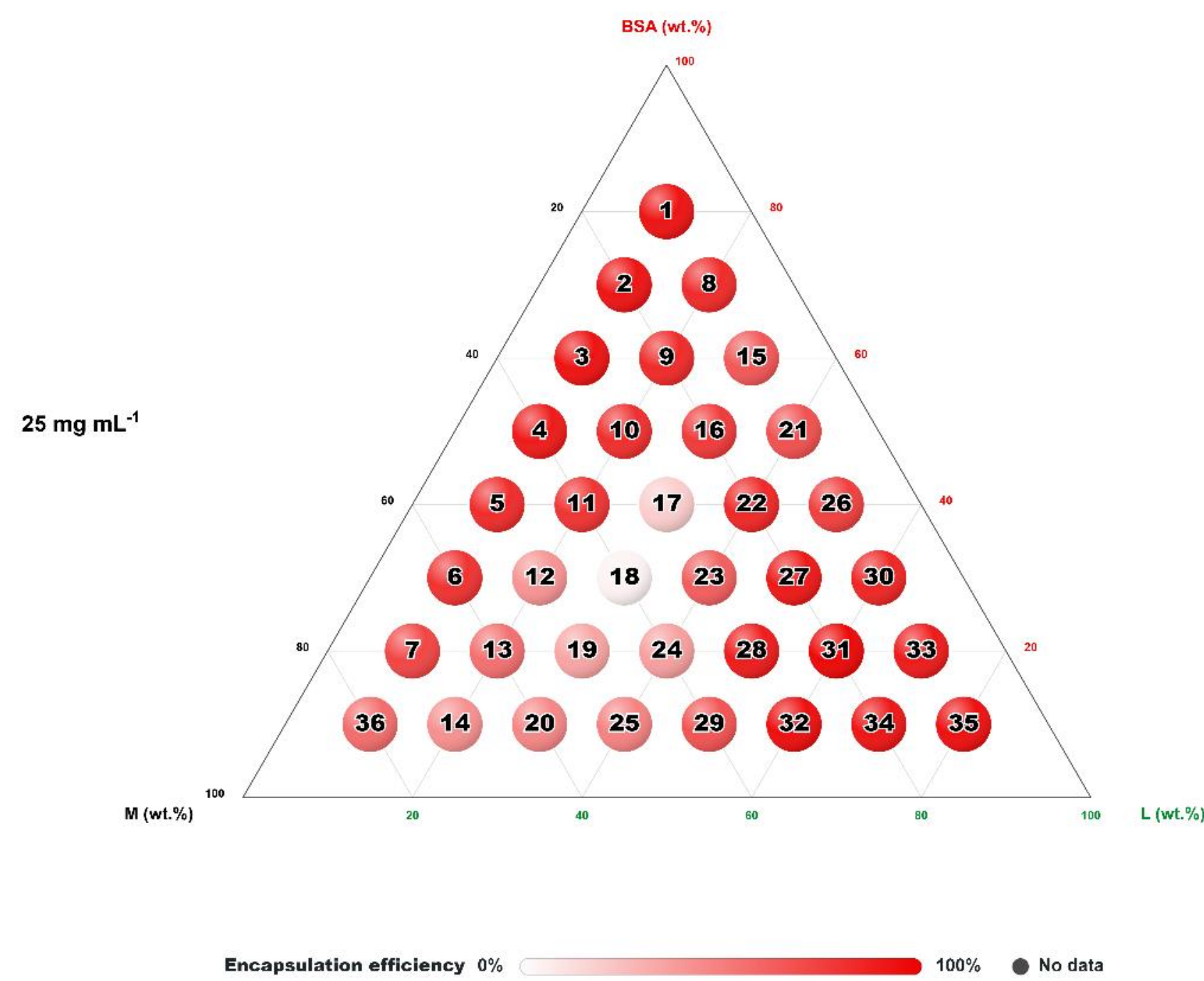

Figure S35. TD-EE% for the WW (TD-H_2O) system prepared at TC = 25 mg mL^{-1}.

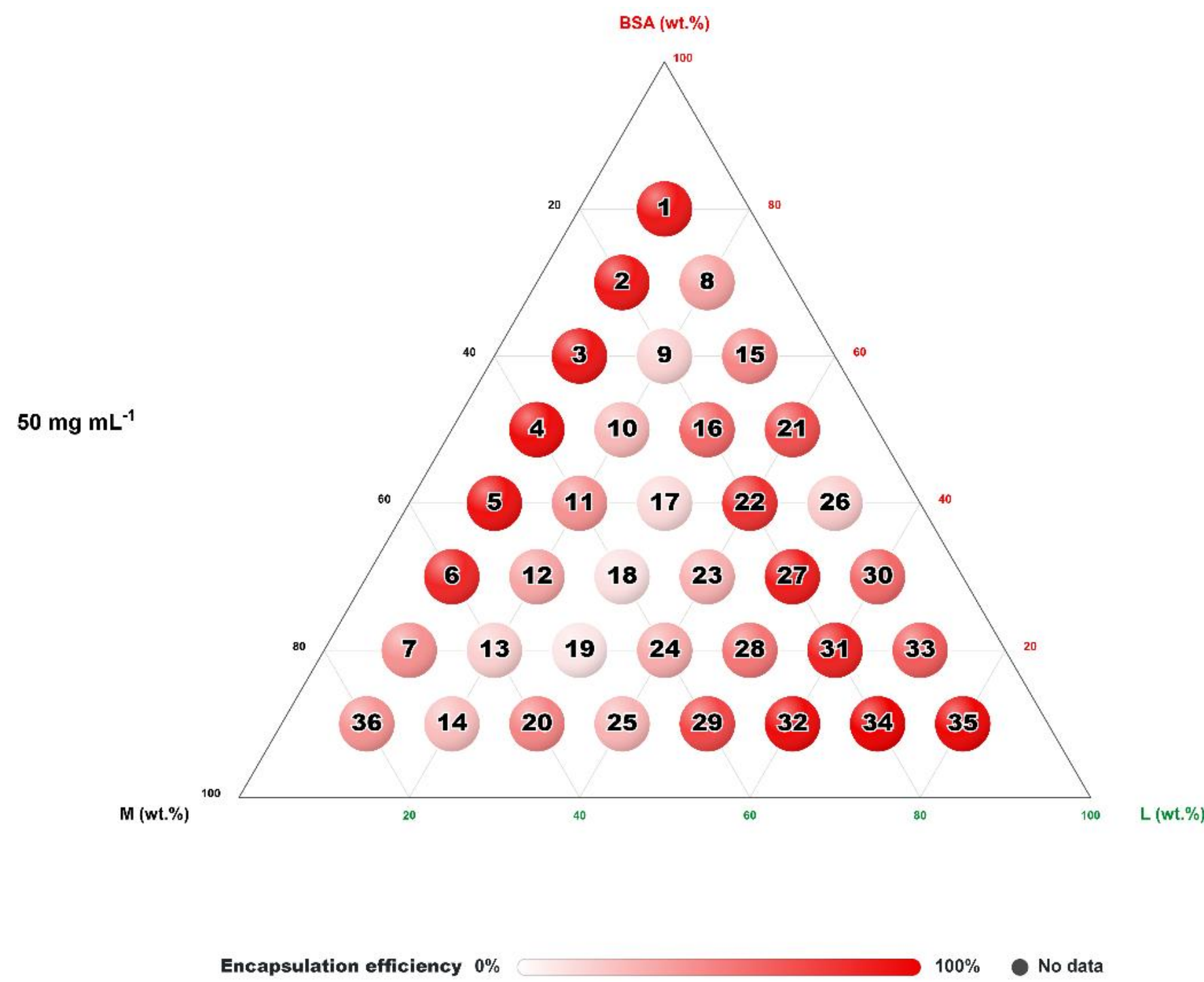

Figure S36. TD-EE% for the WW (TD-H_2O) system prepared at TC = 50 mg mL^{-1}.

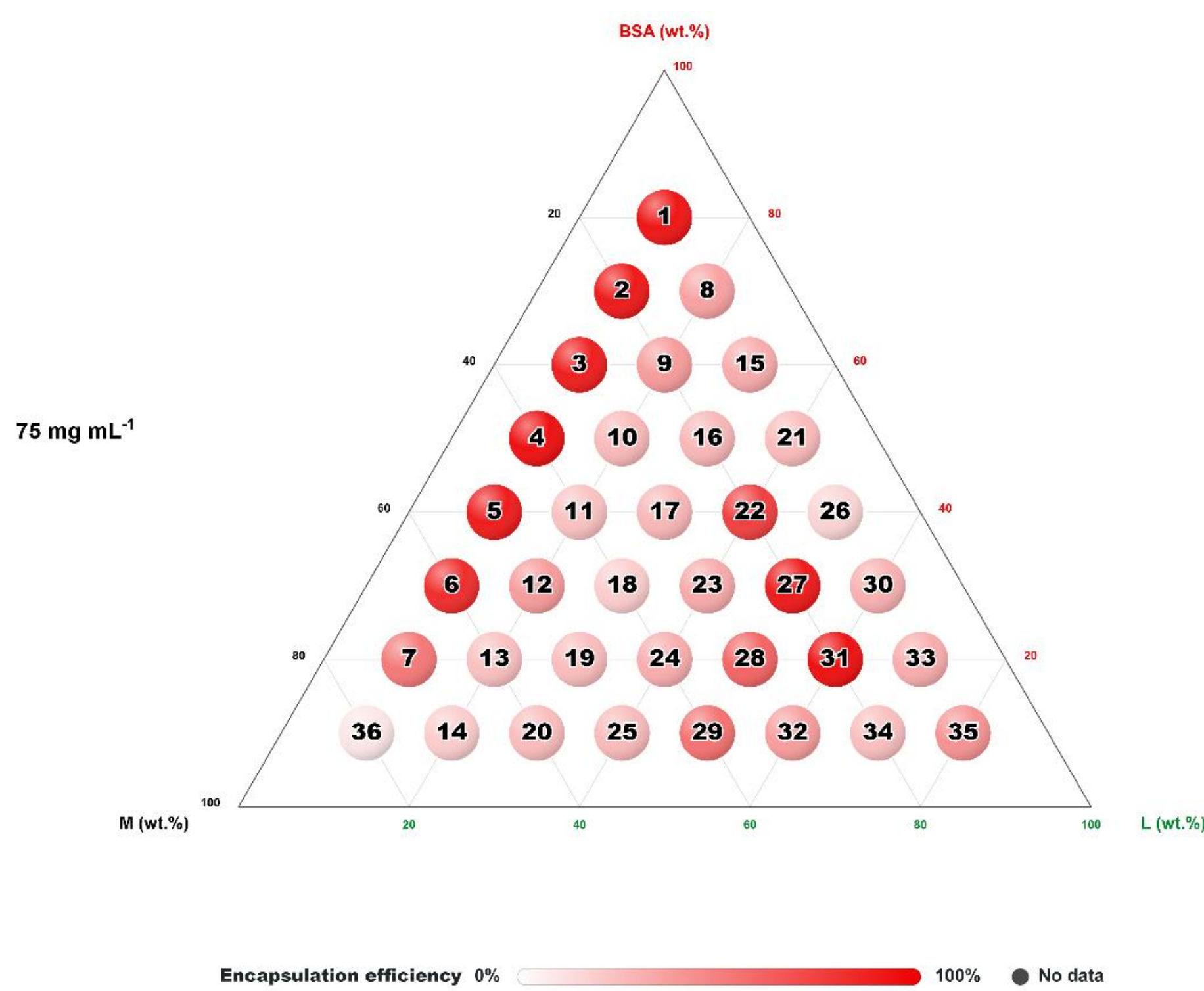

Figure S37. TD-EE% for the WW (TD-H_2O) system prepared at TC = 75 mg mL^{-1}.

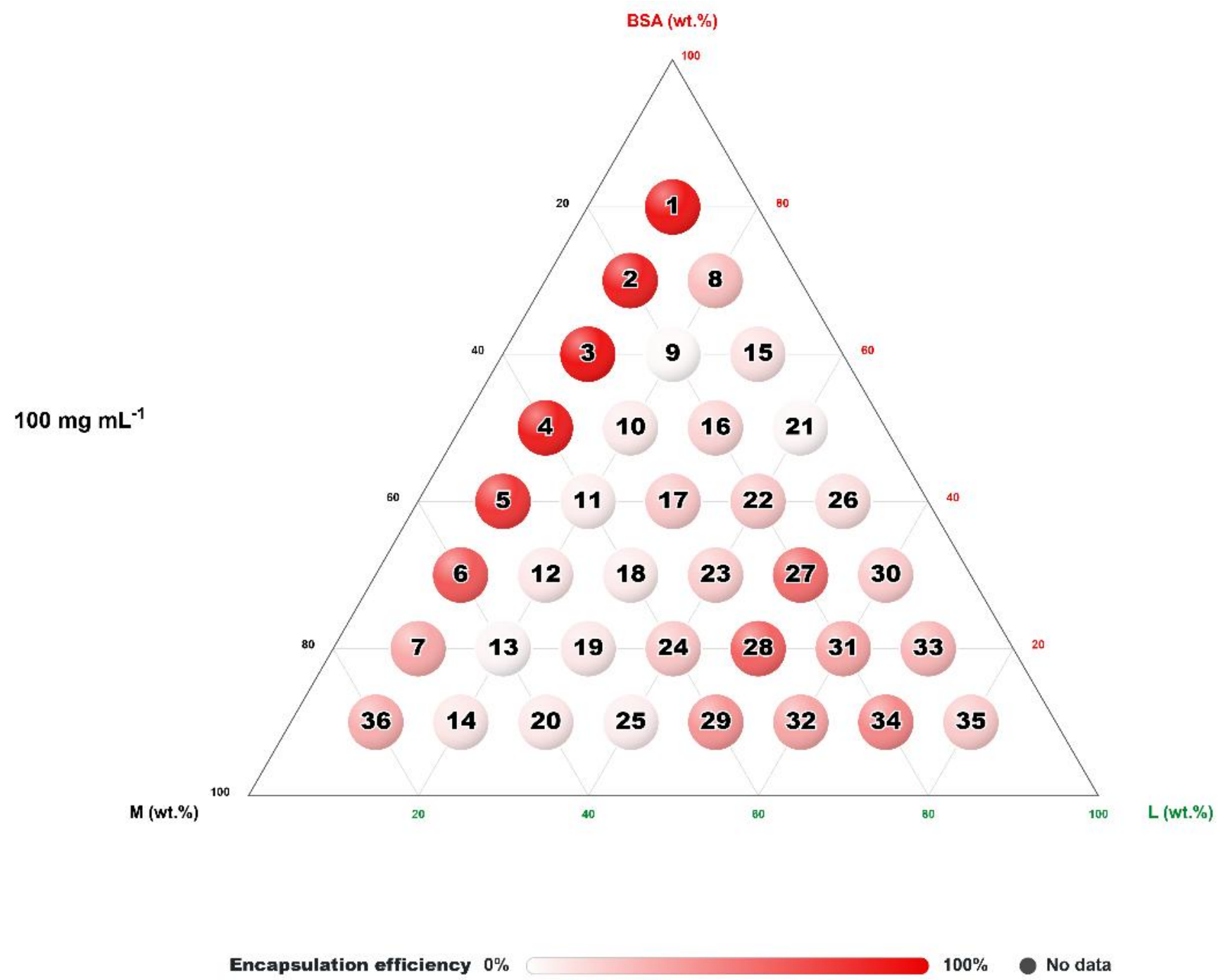

Figure S38. TD-EE% for the WW (TD-H_2O) system prepared at TC = 100 mg mL^{-1}.

4.2 TDs of LC%

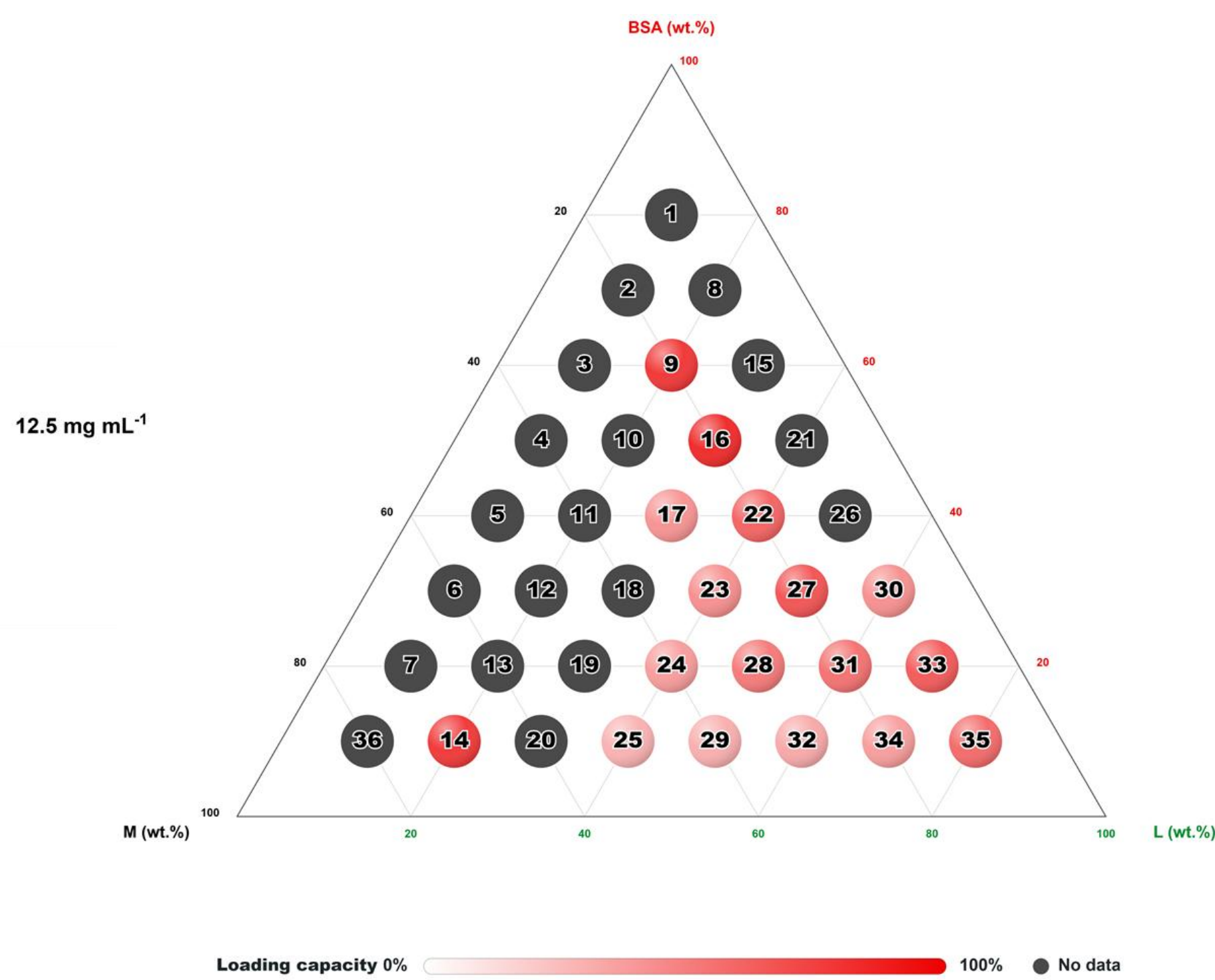

Figure S39. TD-LC% for the WW (TD-H_2O) system prepared at TC = 12.5 mg mL^{-1}.

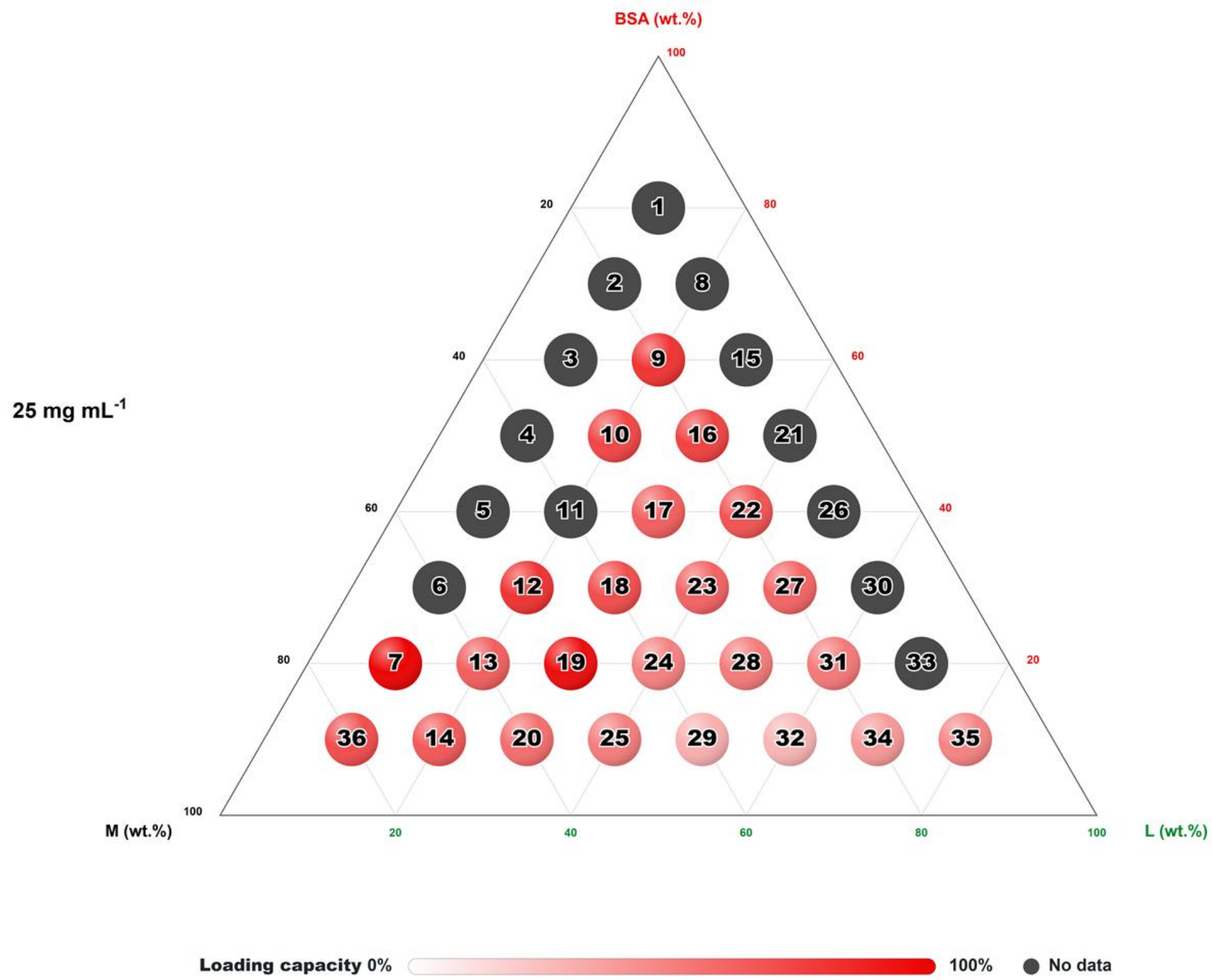

Figure S40. TD-LC% for the WW (TD-H_2O) system prepared at TC = 25 mg mL^{-1}.

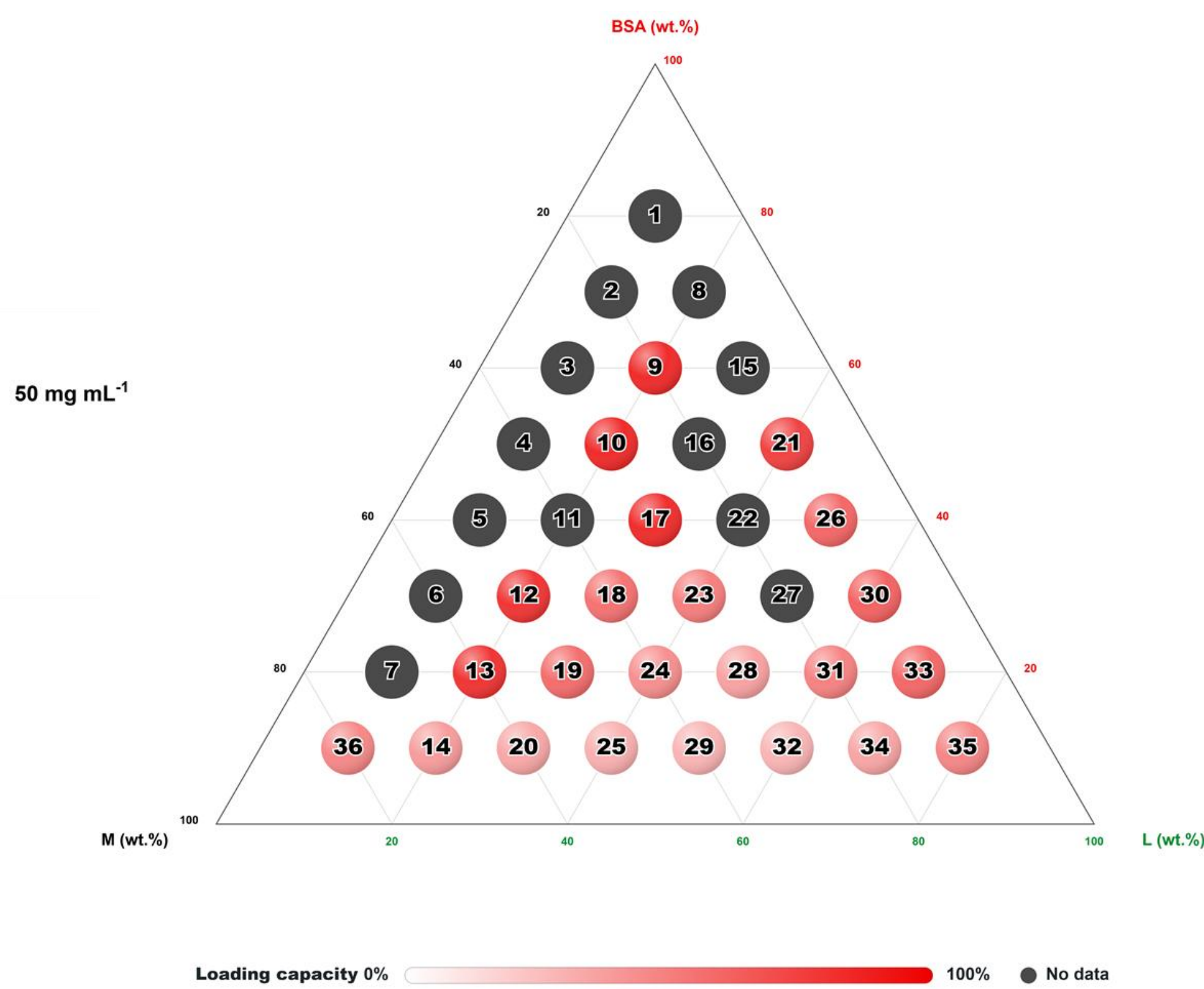

Figure S41. TD-LC% for the WW (TD-H_2O) system prepared at TC = 50 mg mL^{-1}.

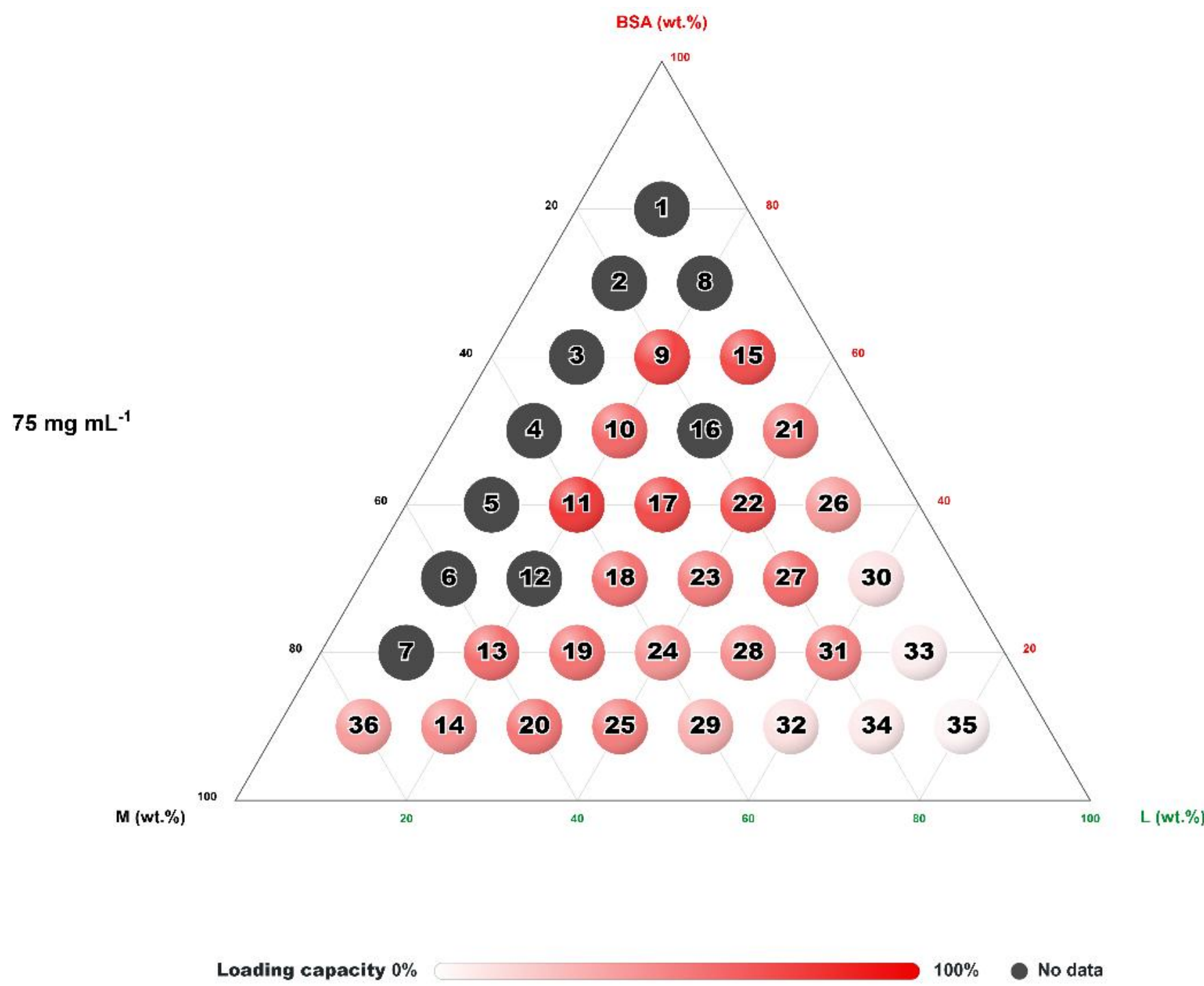

Figure S42. TD-LC% for the WW (TD-H_2O) system prepared at TC = 75 mg mL^{-1}.

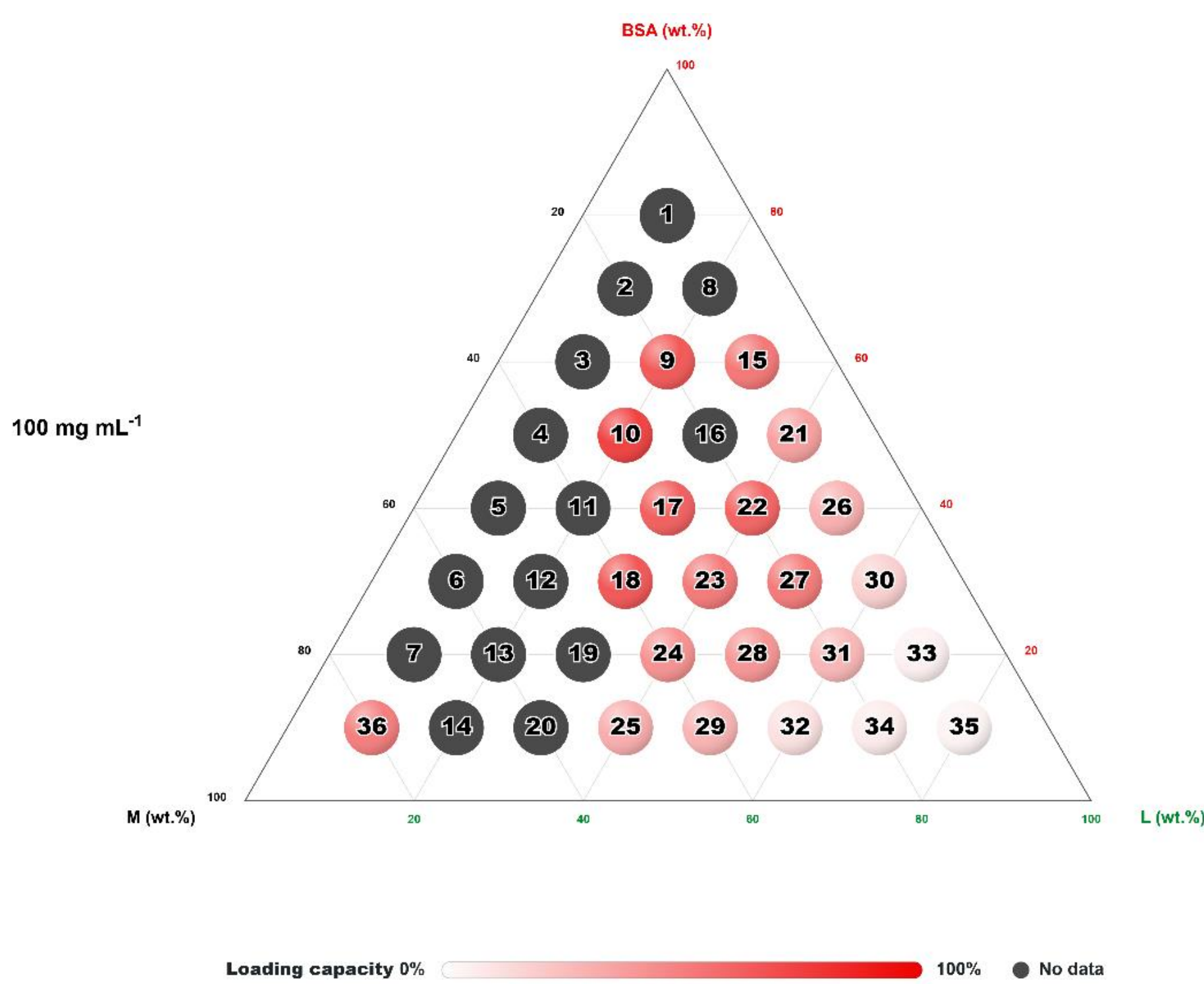

Figure S43. TD-LC% for the WW (TD-H_2O) system prepared at TC = 100 mg mL^{-1}.

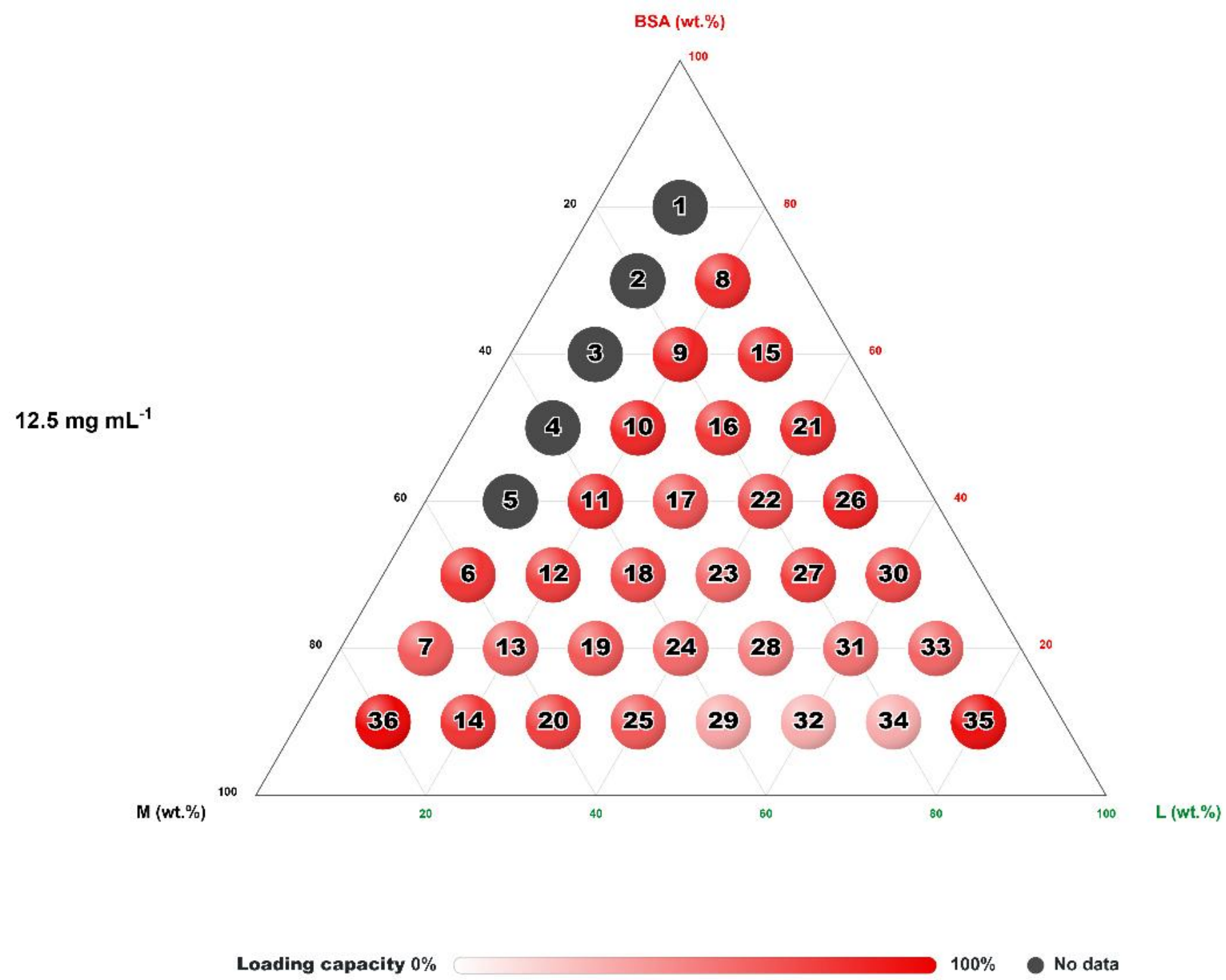

Figure S44. TD-LC% for the EW (TD-EtOH) system prepared at TC = 12.5 mg mL^{-1}.

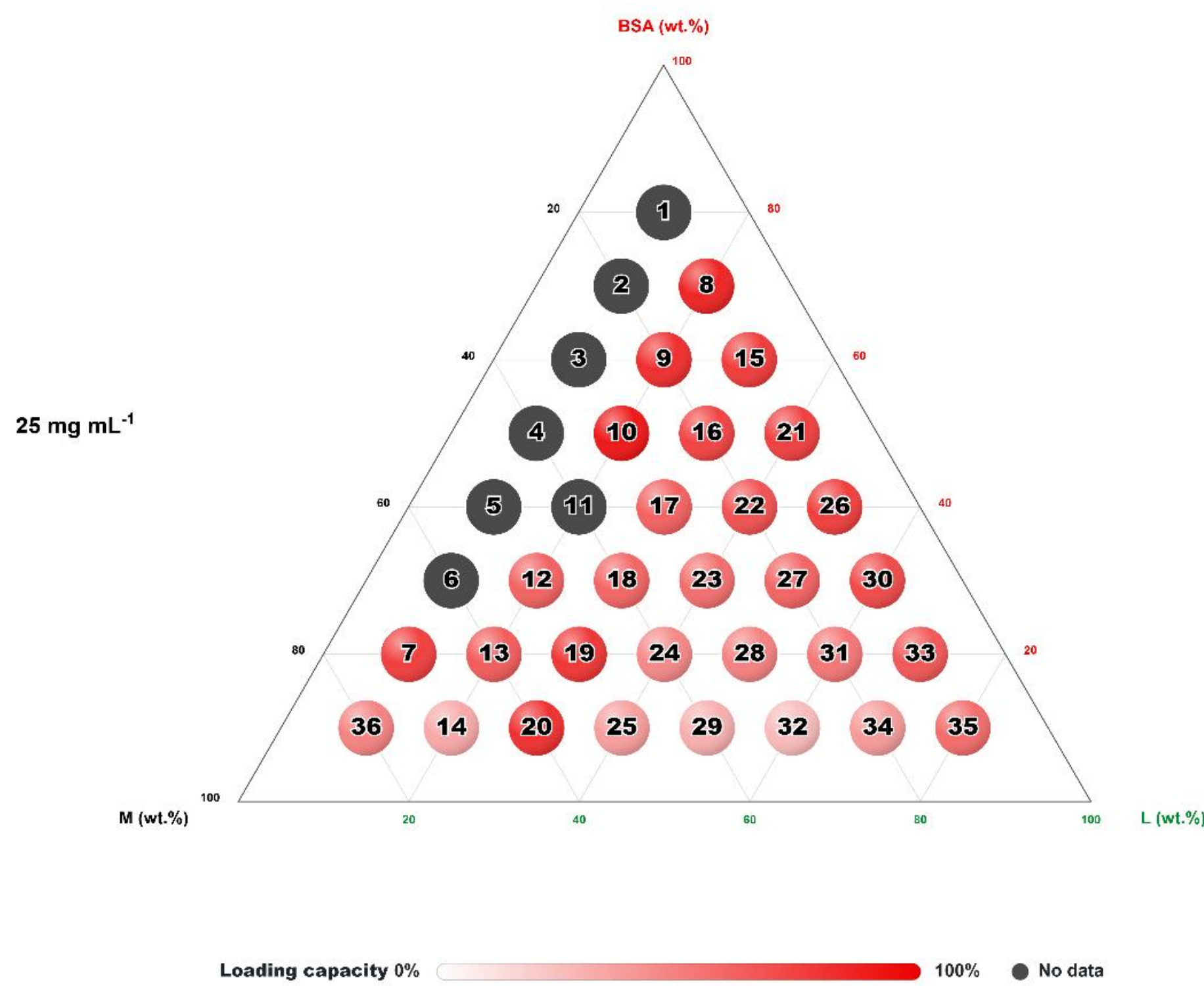

Figure S45. TD-LC% for the EW (TD-EtOH) system prepared at TC = 25 mg mL^{-1}.

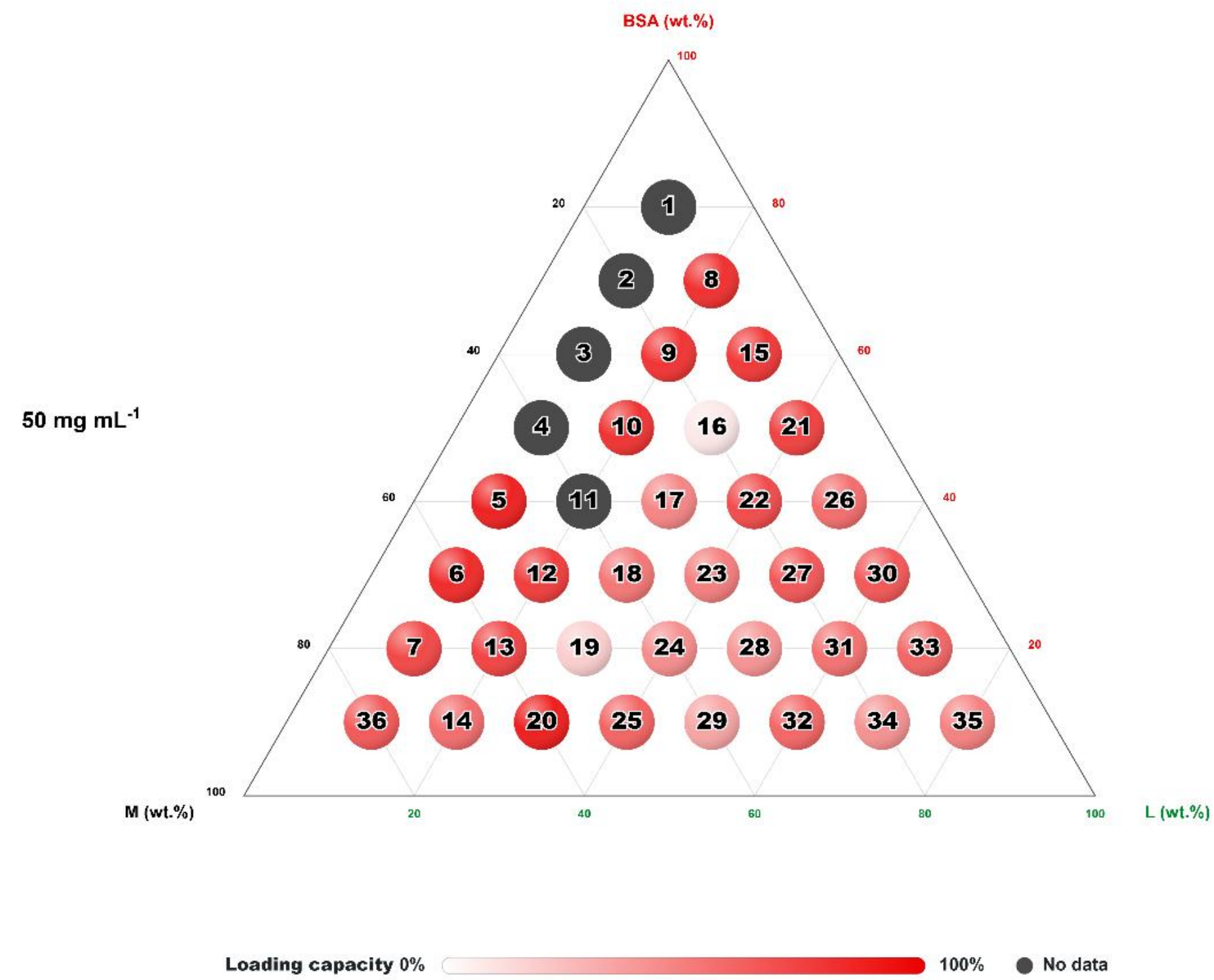

Figure S46. TD-LC% for the EW (TD-EtOH) system prepared at TC = 50 mg mL^{-1}.

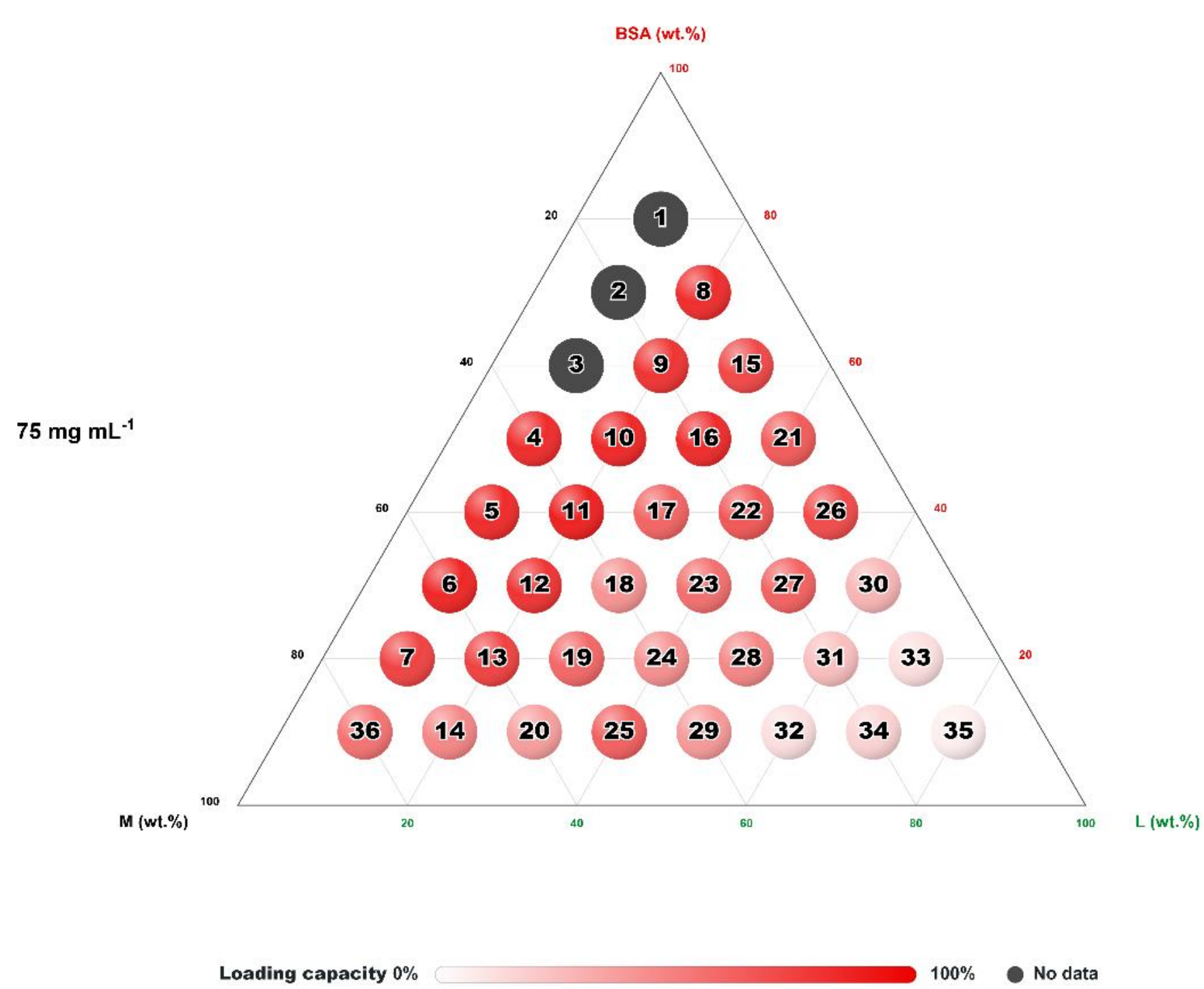

Figure S47. TD-LC% for the EW (TD-EtOH) system prepared at TC = 75 mg mL^{-1}.

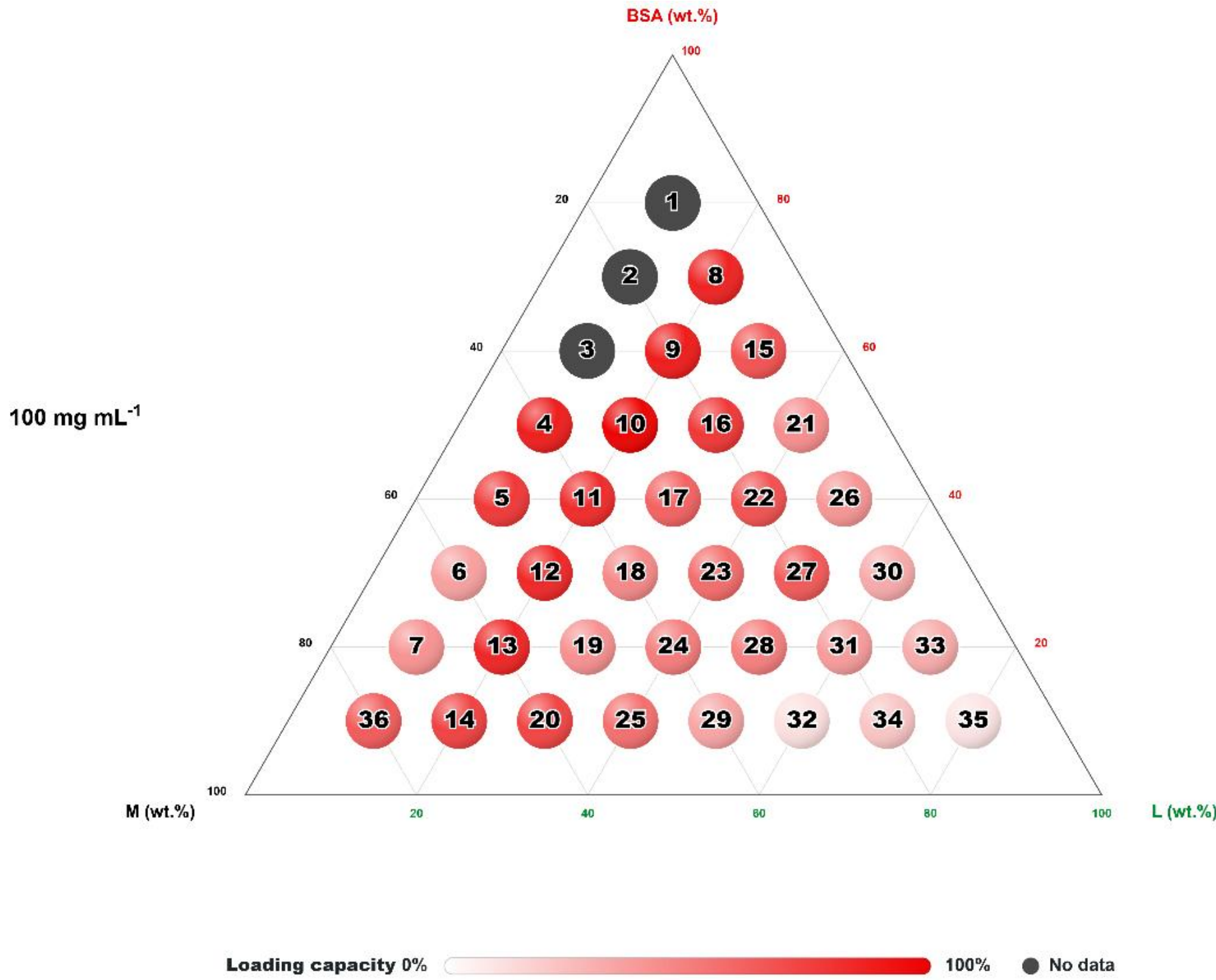

Figure S48. TD-LC% for the EW (TD-EtOH) system prepared at TC = 100 mg mL^{-1}.

4.3 Relationship Between Amorphous Fraction, IR ratio, EE% and LC%

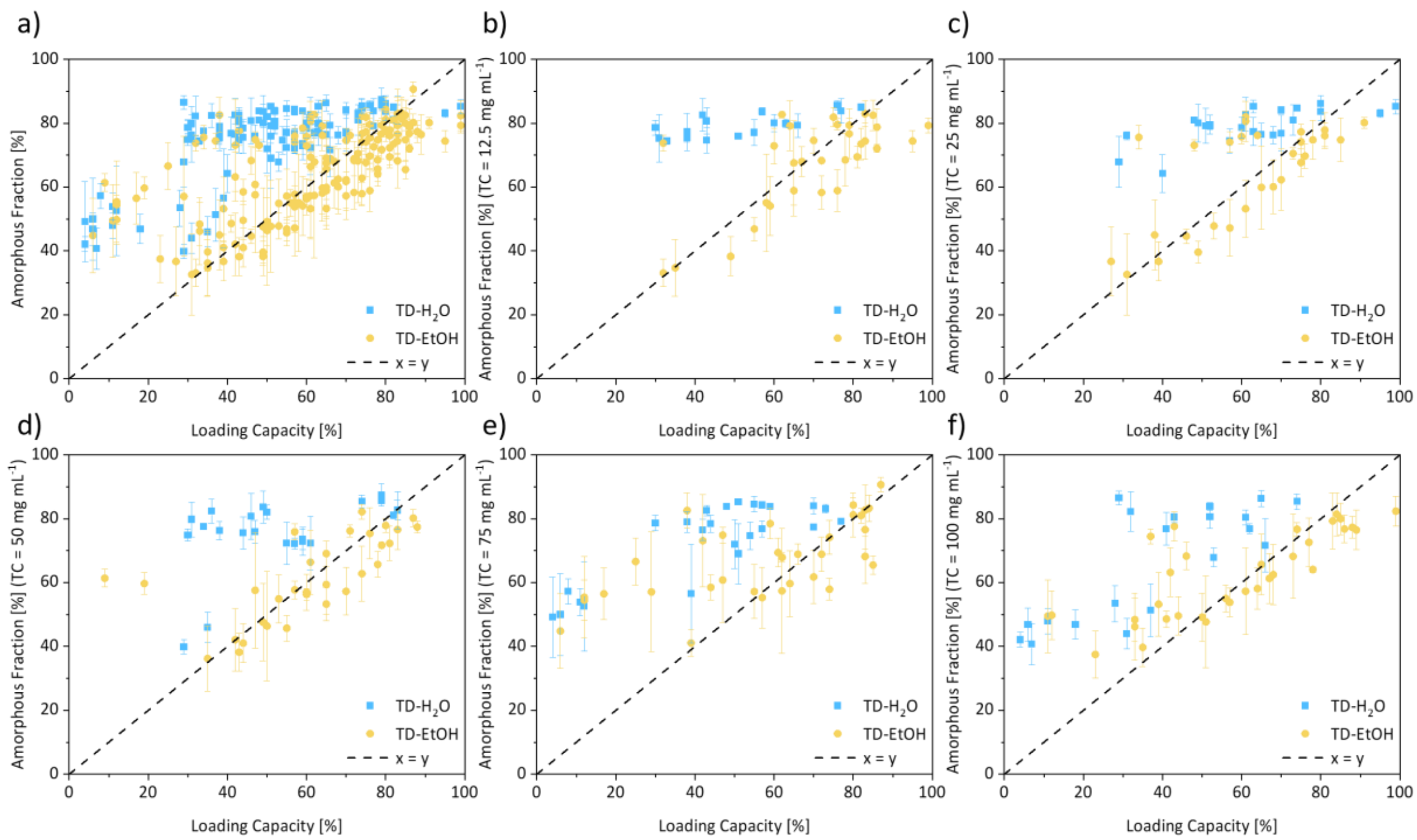

Figure S49. Relationship between amorphous fraction and LC% for the synthesized samples. a) All samples, excluding those with an amorphous fraction of 100% (94 samples in total). b–f) Samples grouped by each TC, excluding those with an amorphous fraction of 100%: b) 12.5 mg mL^{-1}, c) 25 mg mL^{-1}, d) 50 mg mL^{-1}, e) 75 mg mL^{-1}, and f) 100 mg mL^{-1}. Blue squares represent TD-H$_2$O, and yellow circles represent TD-EtOH. The black dashed line indicates the reference line $x = y$. The TD-H$_2$O samples generally lie above the x=y line, indicating that their PXRD-derived amorphous fraction exceeds the LC% and may therefore include contributions from both BSA and amorphous ZIF. In contrast, the TD-EtOH samples are distributed closer to the (x=y) line, consistent with the ethanol-induced crystallization of amorphous ZIF into the sod phase, suggesting that BSA is the major contributor to the residual amorphous fraction.

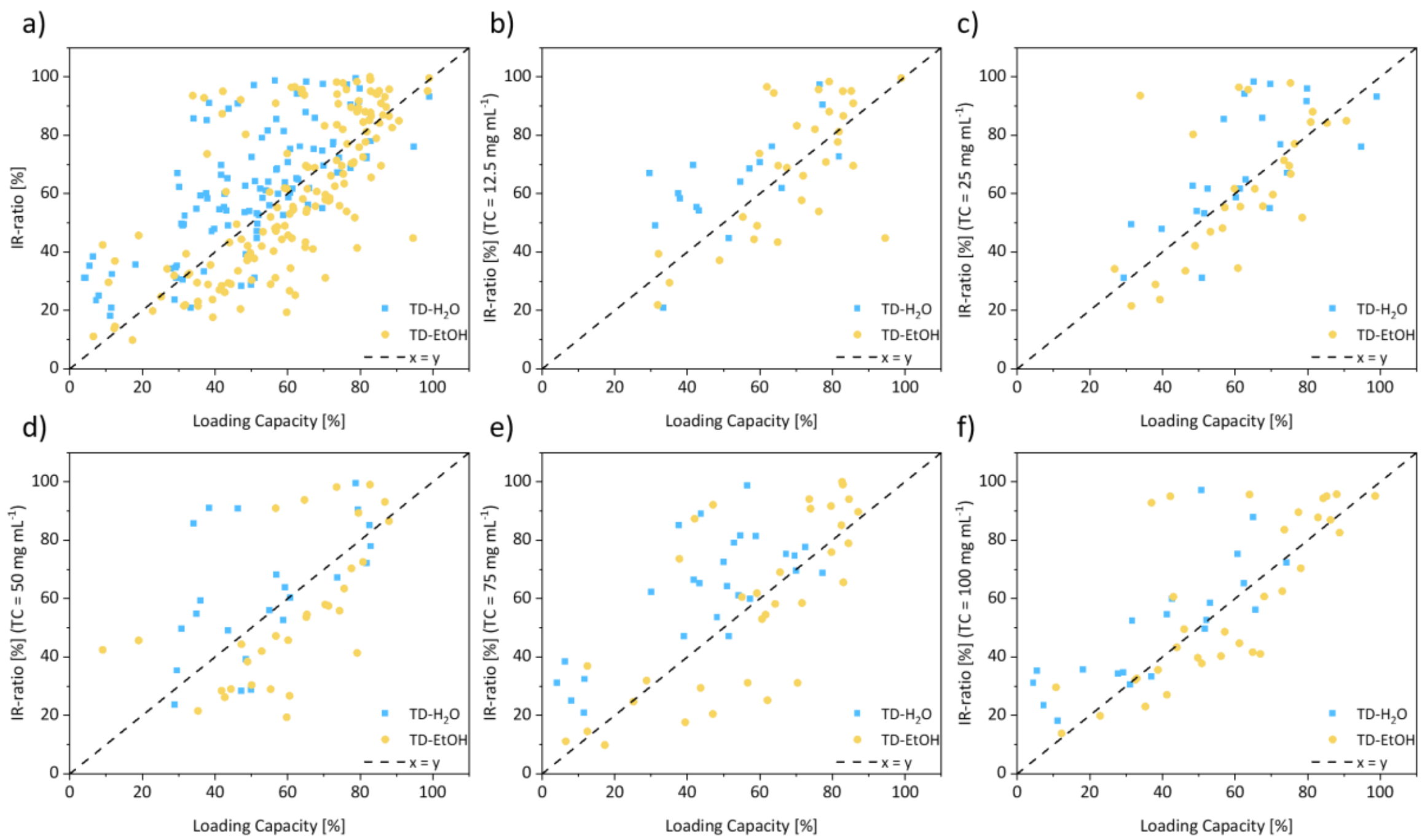

Figure S50. Relationship between IR ratio and LC% for the synthesized samples. a) All samples, excluding those with an amorphous fraction of 100% (94 samples in total). b–f) Samples grouped by each TC, excluding those with an amorphous fraction of 100%: b) 12.5 mg mL^{-1}, c) 25 mg mL^{-1}, d) 50 mg mL^{-1}, e) 75 mg mL^{-1}, and f) 100 mg mL^{-1}. Blue squares represent TD-H_2O, and yellow circles represent TD-EtOH. The black dashed line indicates the reference line x = y. Defined as $ABS_{amide\ I}$ / ($ABS_{amide\ I}$ + $ABS_{Zn–N}$), the IR ratio reflects the relative contribution of BSA to the combined absorbance of representative BSA and ZIF bands, and cannot be considered a quantitative determination of the protein mass fraction. It is therefore a qualitative indicator of the protein loading within the biocomposites. The general increase in the IR ratio with ICP-OES-derived LC% supports its use as a qualitative indicator of protein loading. It should be noted that, beyond some scattering, the most IR ratio values of TD-H_2O samples lie above the LC% values, while the most IR ratio values of TD-EtOH samples lie close to the LC% values. This could

partially be ascribed to the presence of residual water in the TD-H_2O samples, which might increase the absorbance measured at 1650 cm^{-1} and lead to an overestimation of the protein content.

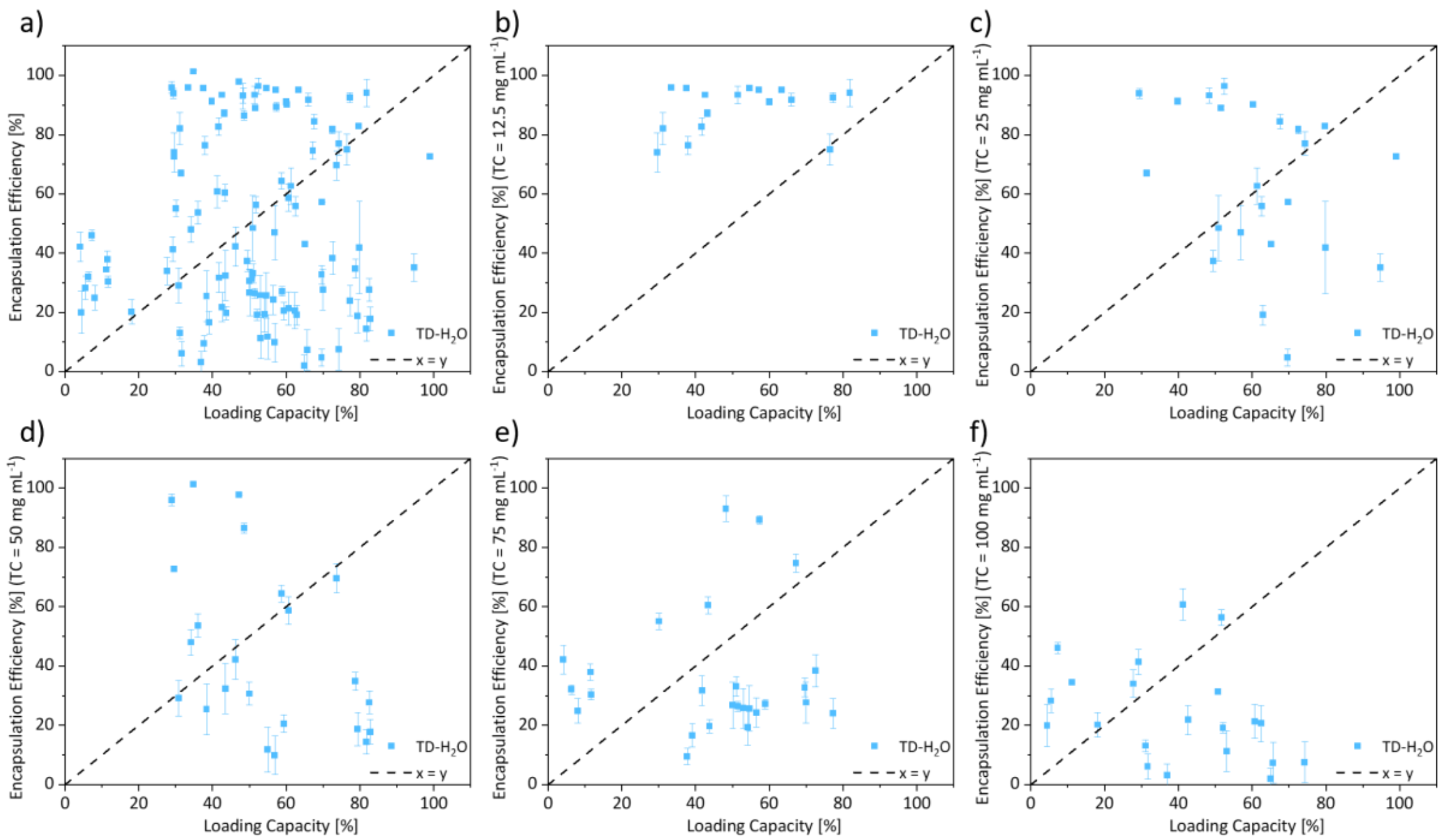

Figure S51. Relationship between EE% and LC% for the synthesized samples. a) All TD-H_2O samples, excluding those with an amorphous fraction of 100% (80 samples in total). b–f) Samples grouped by each TC, excluding those with an amorphous fraction of 100%: b) 12.5 mg mL^{-1}, c) 25 mg mL^{-1}, d) 50 mg mL^{-1}, e) 75 mg mL^{-1}, and f) 100 mg mL^{-1}. Blue squares represent TD-H_2O, and the black dashed line indicates the reference line x = y. No correlation was observed between LC% and EE%. A direct correlation is not necessarily expected because EE% quantifies the fraction of BSA immobilized before washing relative to the amount of BSA initially added, and does not explicitly include the mass of solid ZIF produced. In contrast, LC% represents the mass fraction of BSA relative to the total mass of the recovered biocomposite. Consequently, LC% depends on both the amount of BSA encapsulated and the amount of ZIF formed during the synthesis.

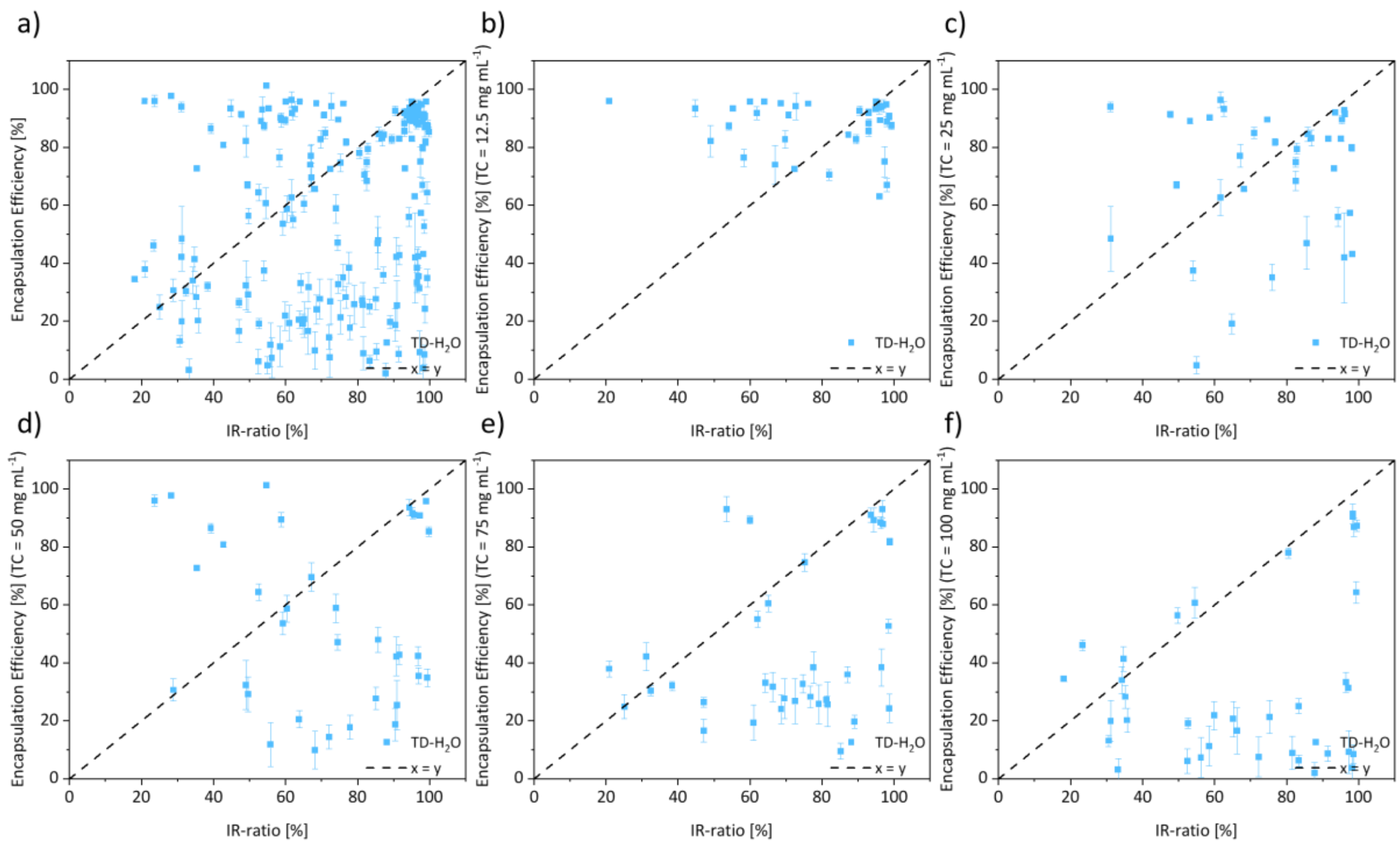

Figure S52. Relationship between IR ratio and EE% for the synthesized samples. a) All TD-H_2O samples. b–f) Samples grouped by each TC: b) 12.5 mg mL^{-1}, c) 25 mg mL^{-1}, d) 50 mg mL^{-1}, e) 75 mg mL^{-1}, and f) 100 mg mL^{-1}. Blue squares represent TD-H_2O, the black dashed line indicates the reference line x = y. No correlation was observed between IR ratio and EE%. This is expected because EE% quantifies the fraction of BSA that is immobilized in the recovered biocomposite relative to the amount of BSA initially added, and is therefore independent of the mass of solid ZIF produced. In contrast, the IR ratio is indicative of the fraction of BSA within the recovered biocomposite and is therefore influenced by the amount of BSA immobilized and the amount of ZIF formed during the synthesis.

5 ATR–IR Spectral Data

5.1 Spectra

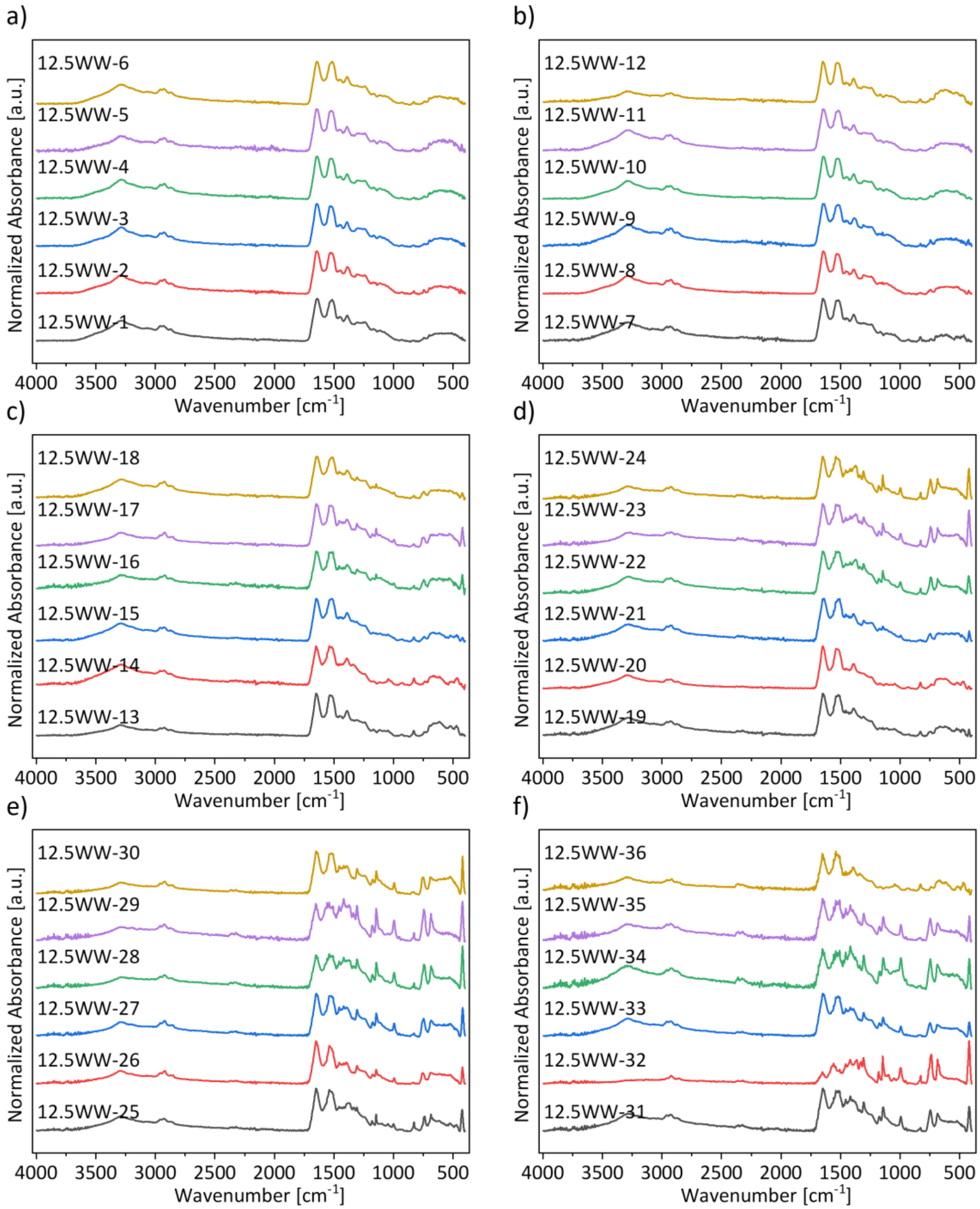

Figure S53. ATR–IR spectra for the WW (TD-H_2O) system prepared at TC = 12.5 mg mL^{-1}.

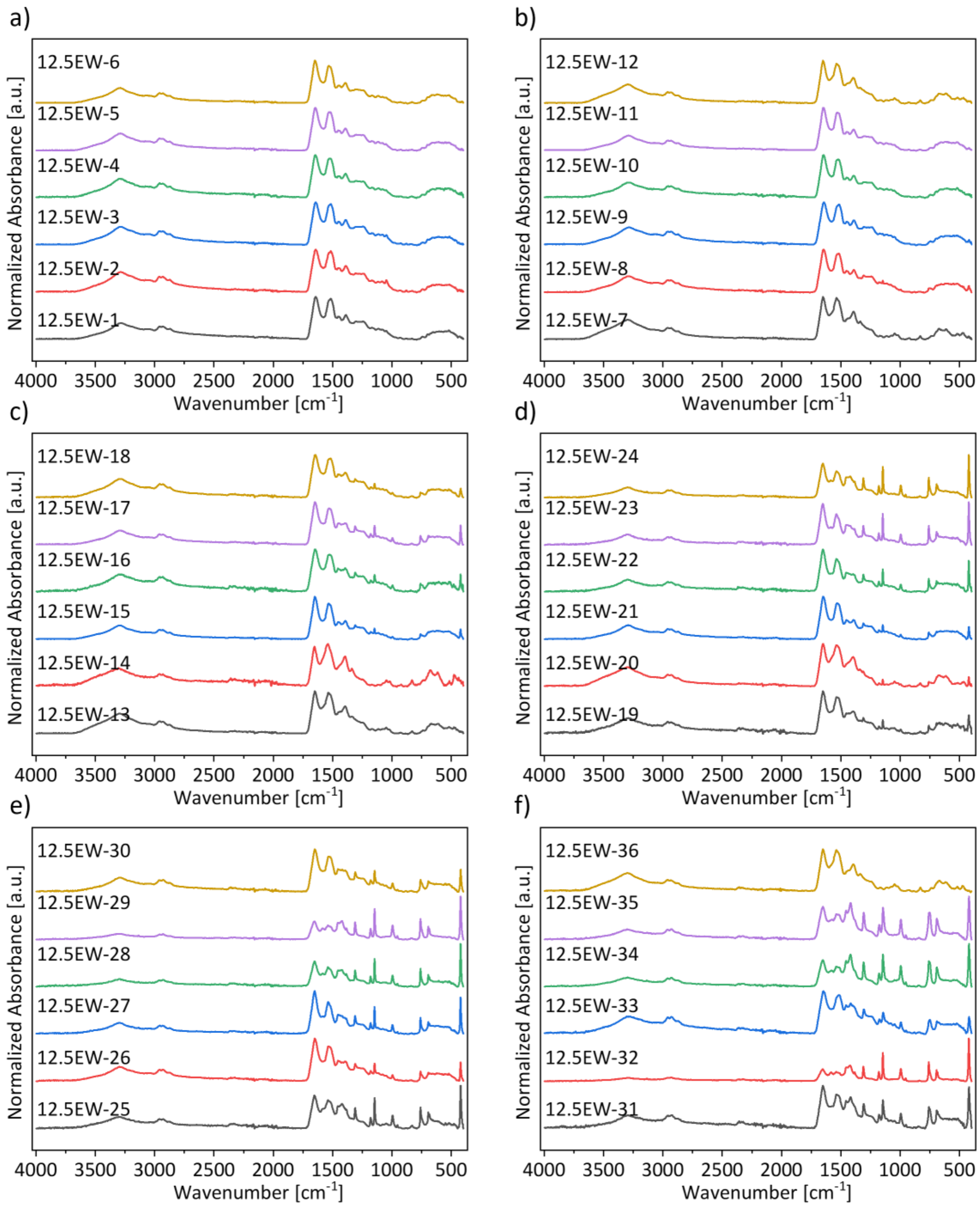

Figure S54. ATR–IR spectra for the EW (TD-EtOH) system prepared at TC = 12.5 mg mL^{-1}.

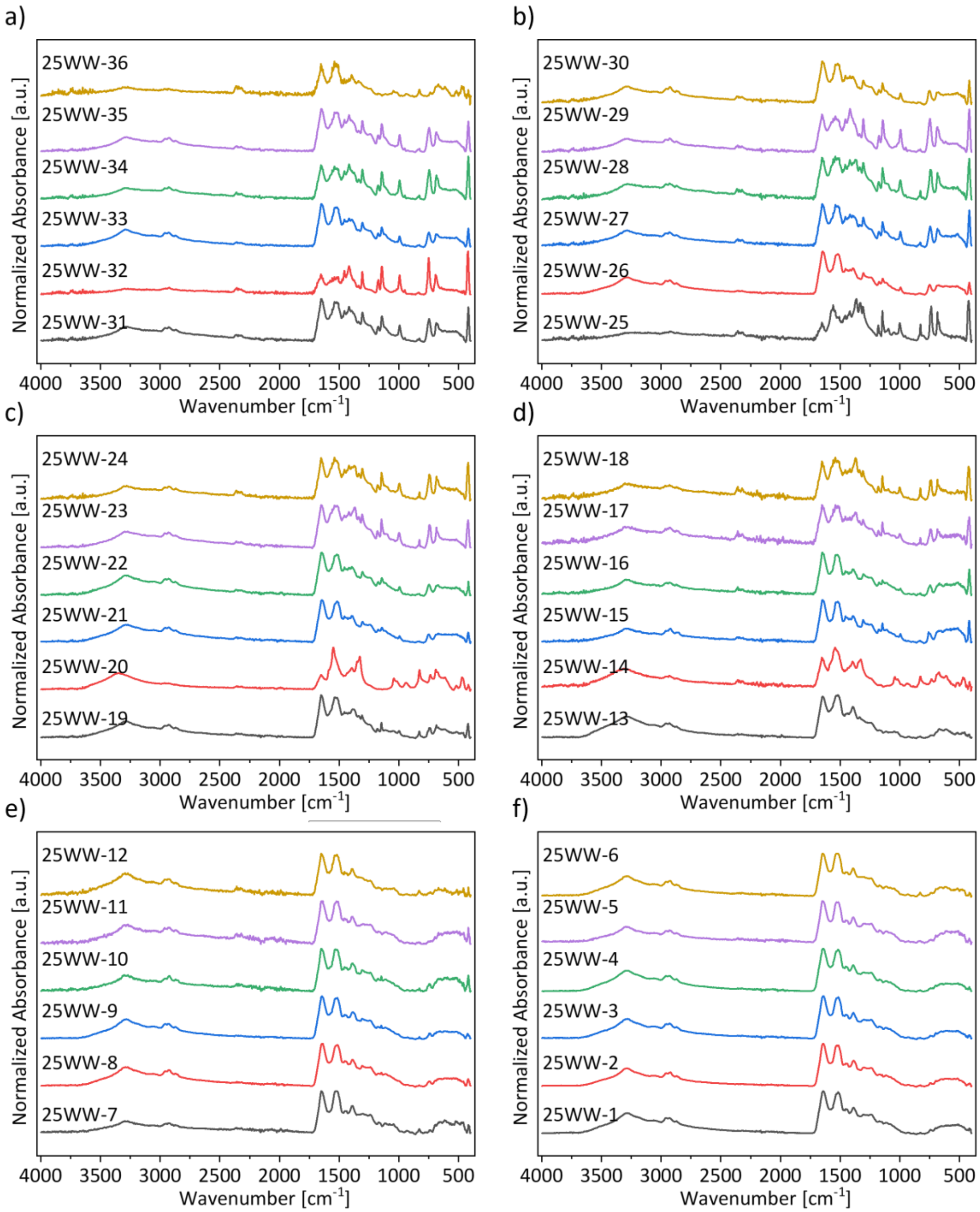

Figure S55. ATR–IR spectra for the WW (TD-H_2O) system prepared at TC = 25 mg mL^{-1}.

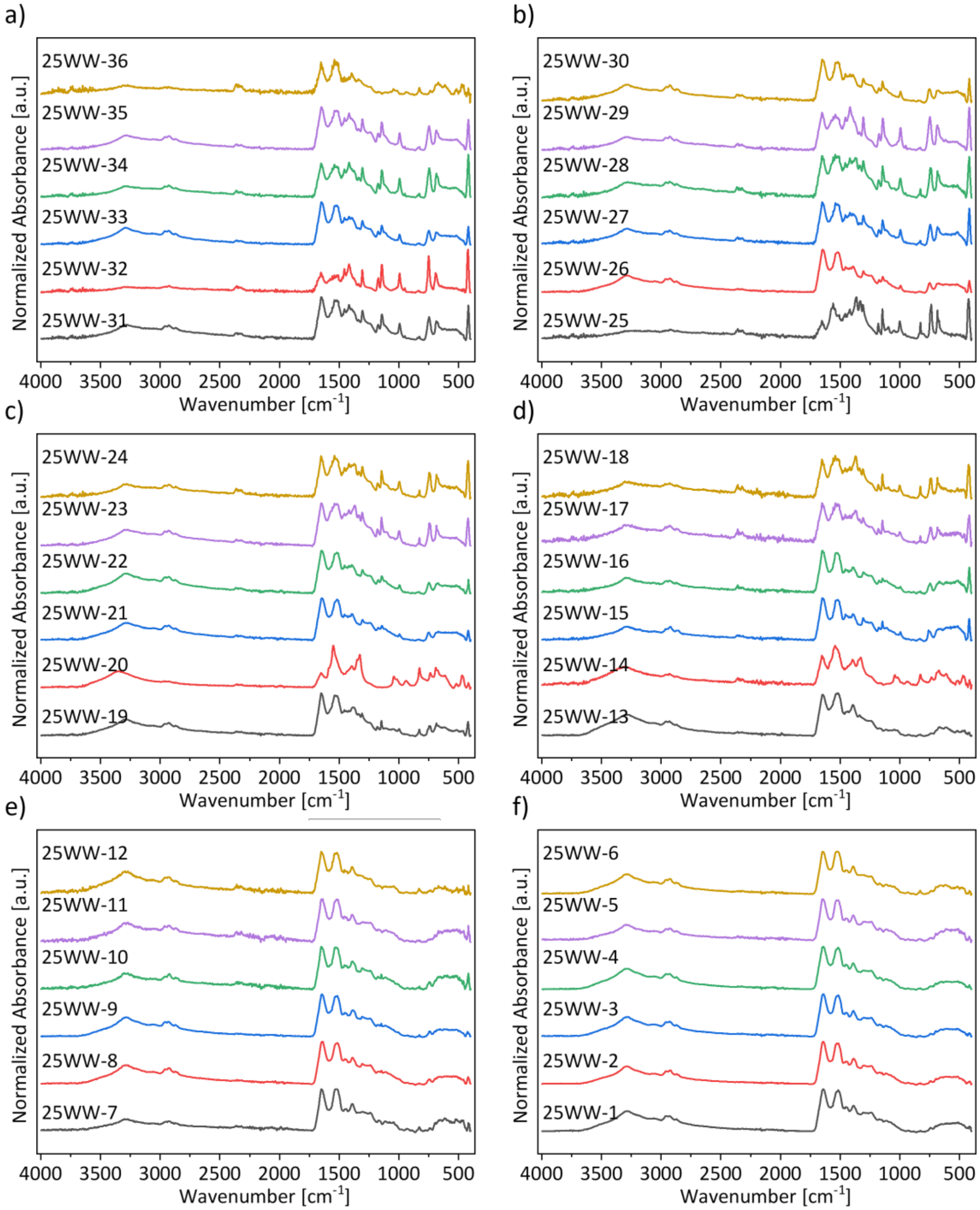

Figure S56. ATR–IR spectra for the EW (TD-EtOH) system prepared at TC = 25 mg mL^{-1}.

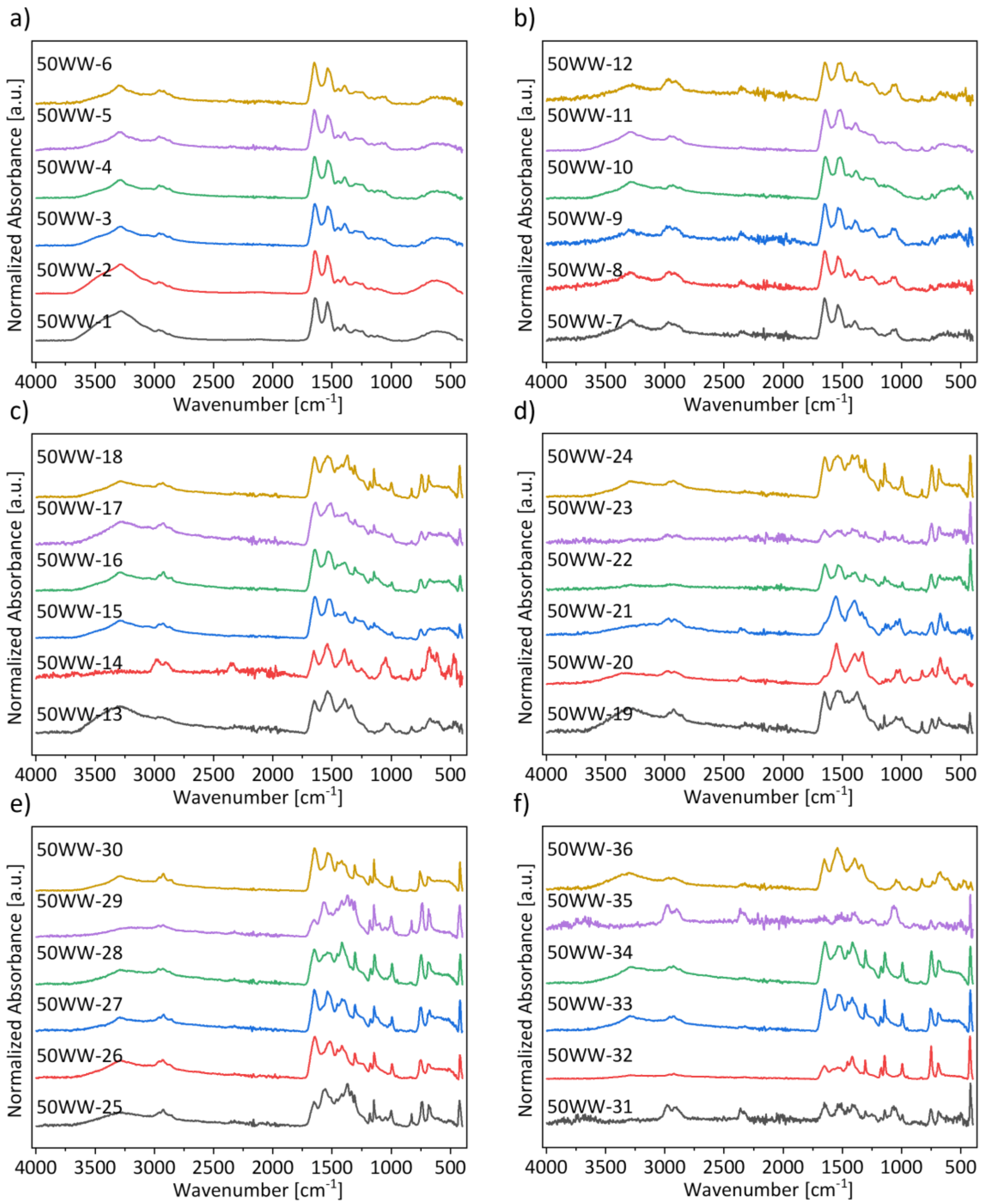

Figure S57. ATR–IR spectra for the WW (TD-H_2O) system prepared at TC = 50 mg mL^{-1}.

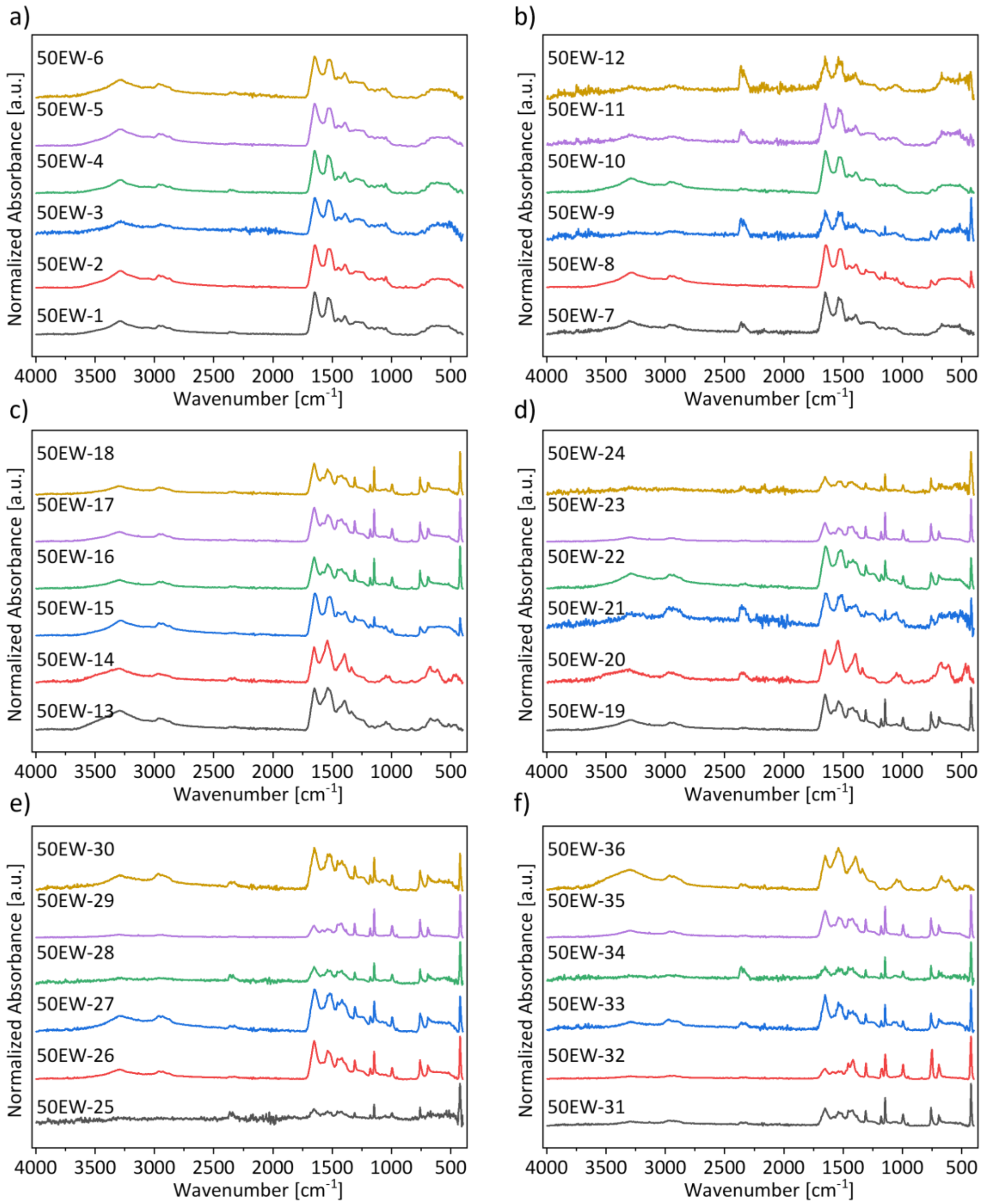

Figure S58. ATR–IR spectra for the EW (TD-EtOH) system prepared at TC = 50 mg mL^{-1}.

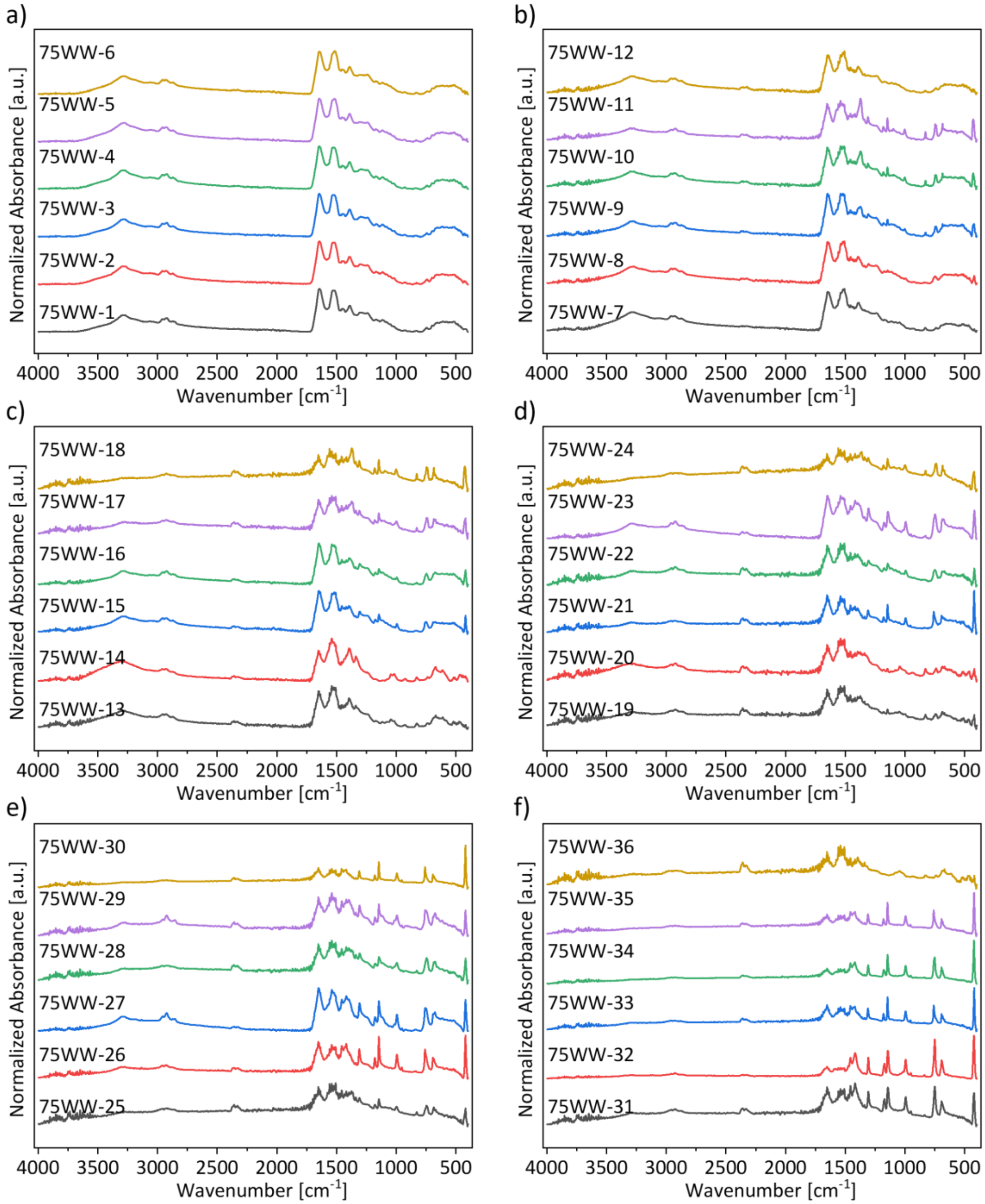

Figure S59. ATR–IR spectra for the WW (TD-H_2O) system prepared at TC = 75 mg mL^{-1}.

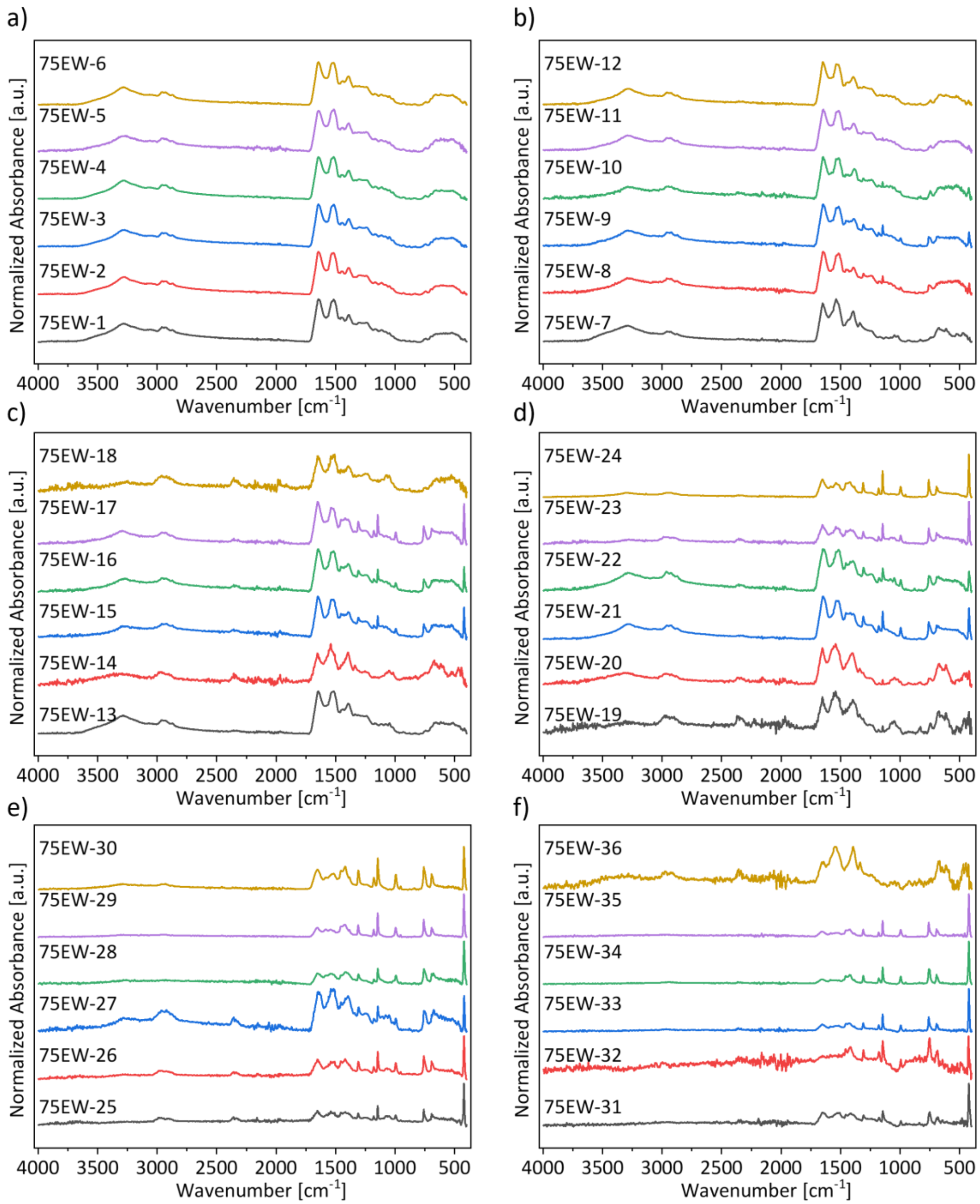

Figure S60. ATR–IR spectra for the EW (TD-EtOH) system prepared at TC = 75 mg mL^{-1}.

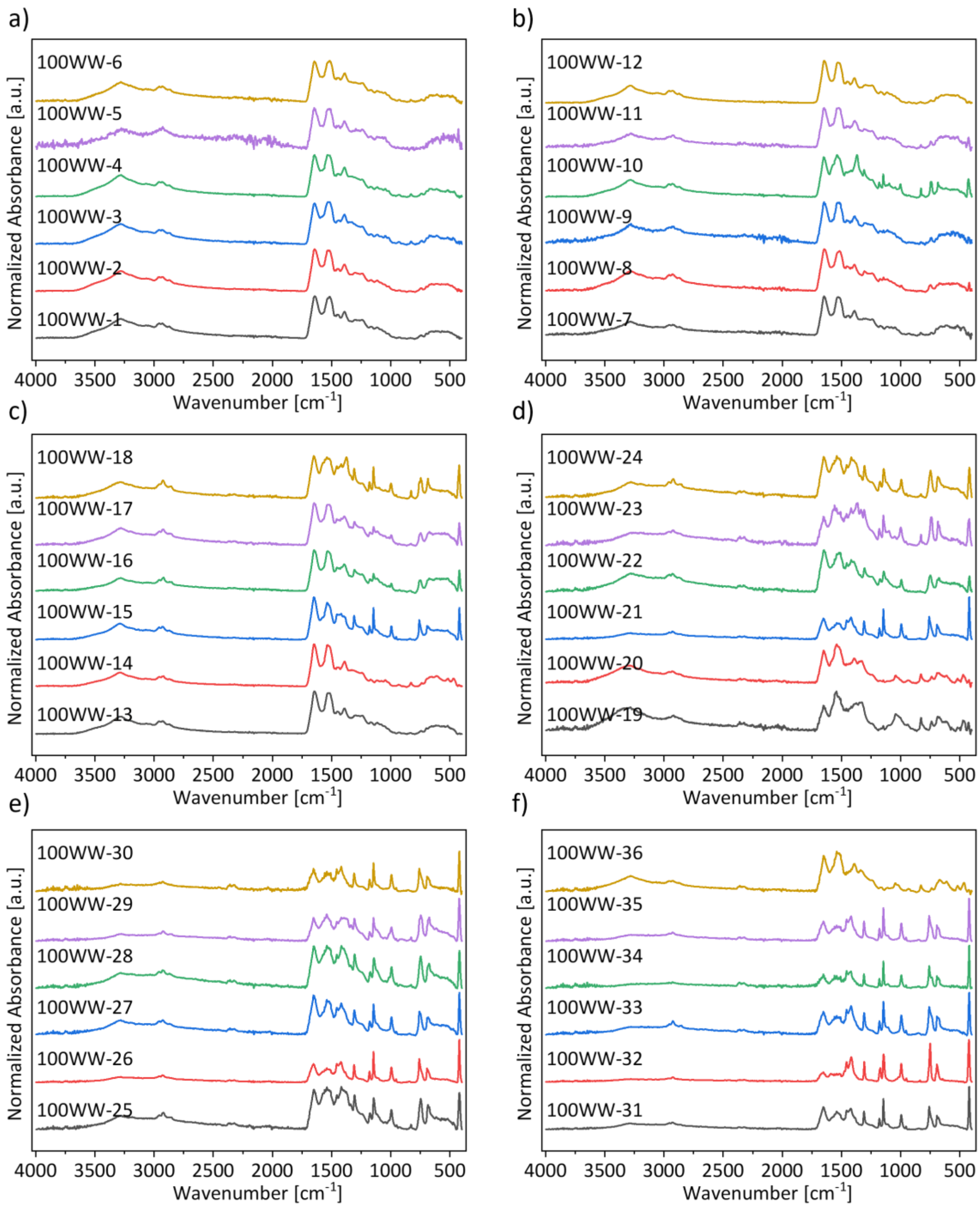

Figure S61. ATR–IR spectra for the WW (TD-H_2O) system prepared at TC = 100 mg mL^{-1}.

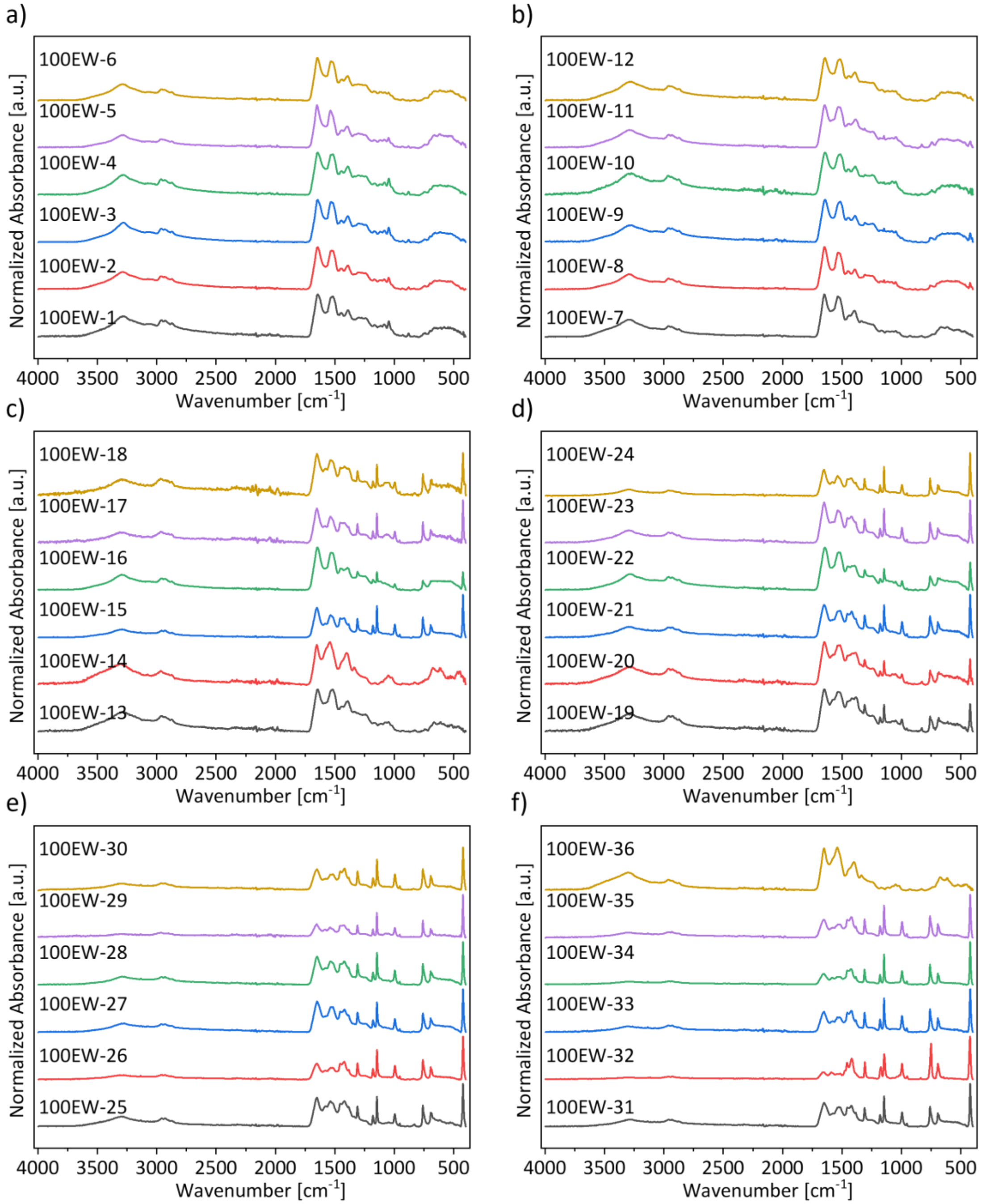

Figure S62. ATR–IR spectra for the EW (TD-EtOH) system prepared at TC = 100 mg mL^{-1}.

5.2 TDs of IR ratio

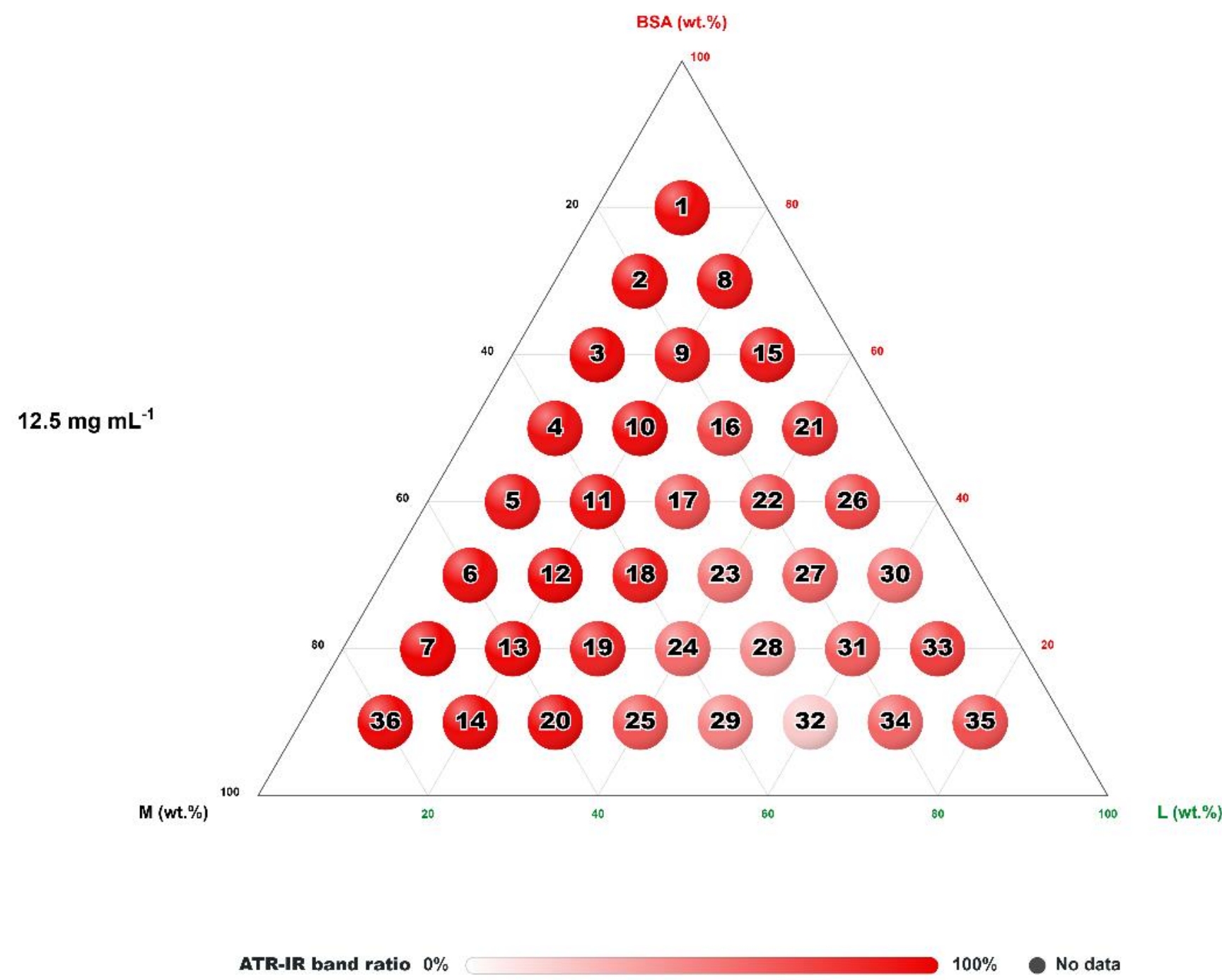

Figure S63. TD-IR ratio for the WW (TD-H_2O) system prepared at TC = 12.5 mg mL^{-1}.

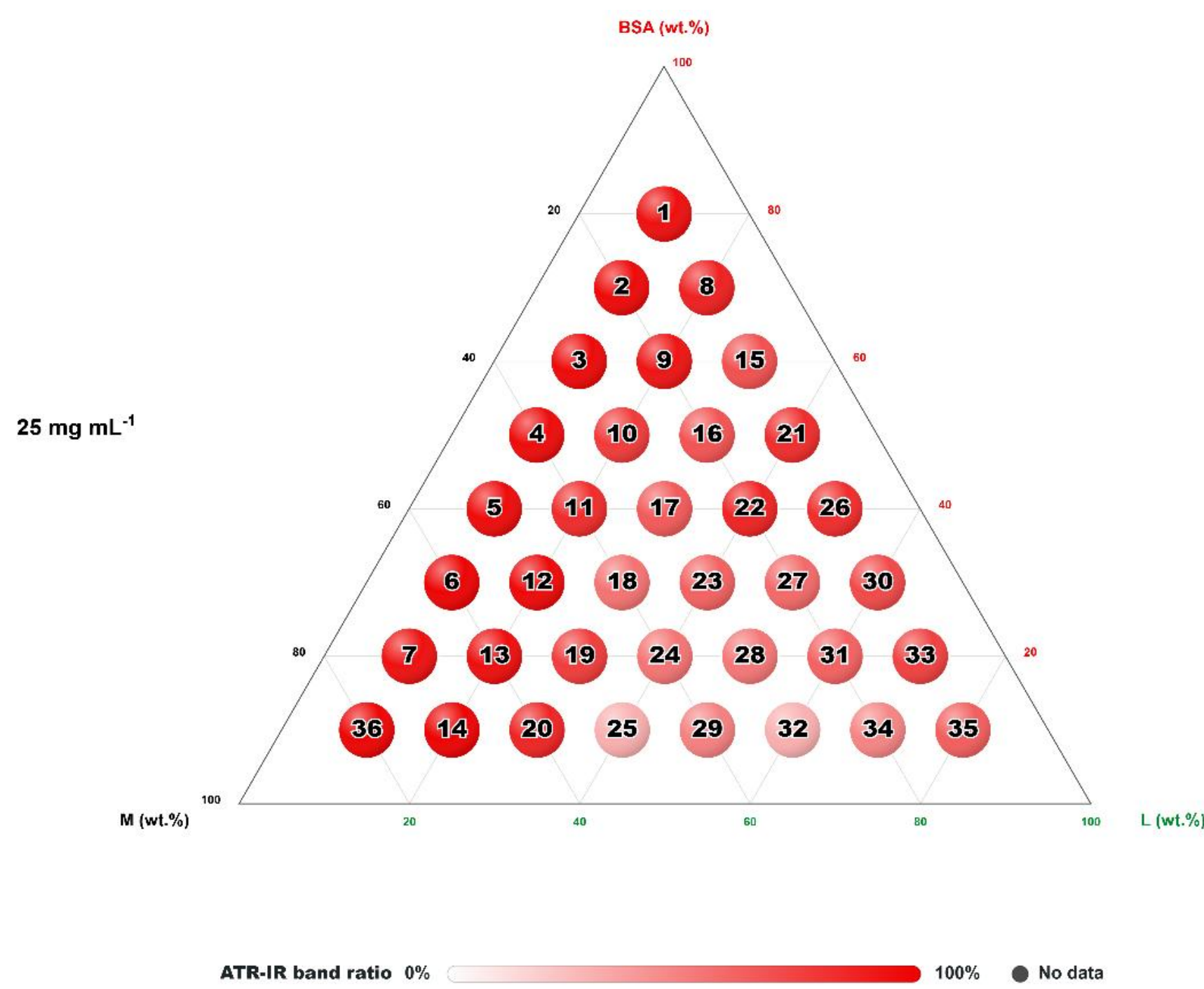

Figure S64. TD-IR ratio for the WW (TD-H_2O) system prepared at TC = 25 mg mL^{-1}.

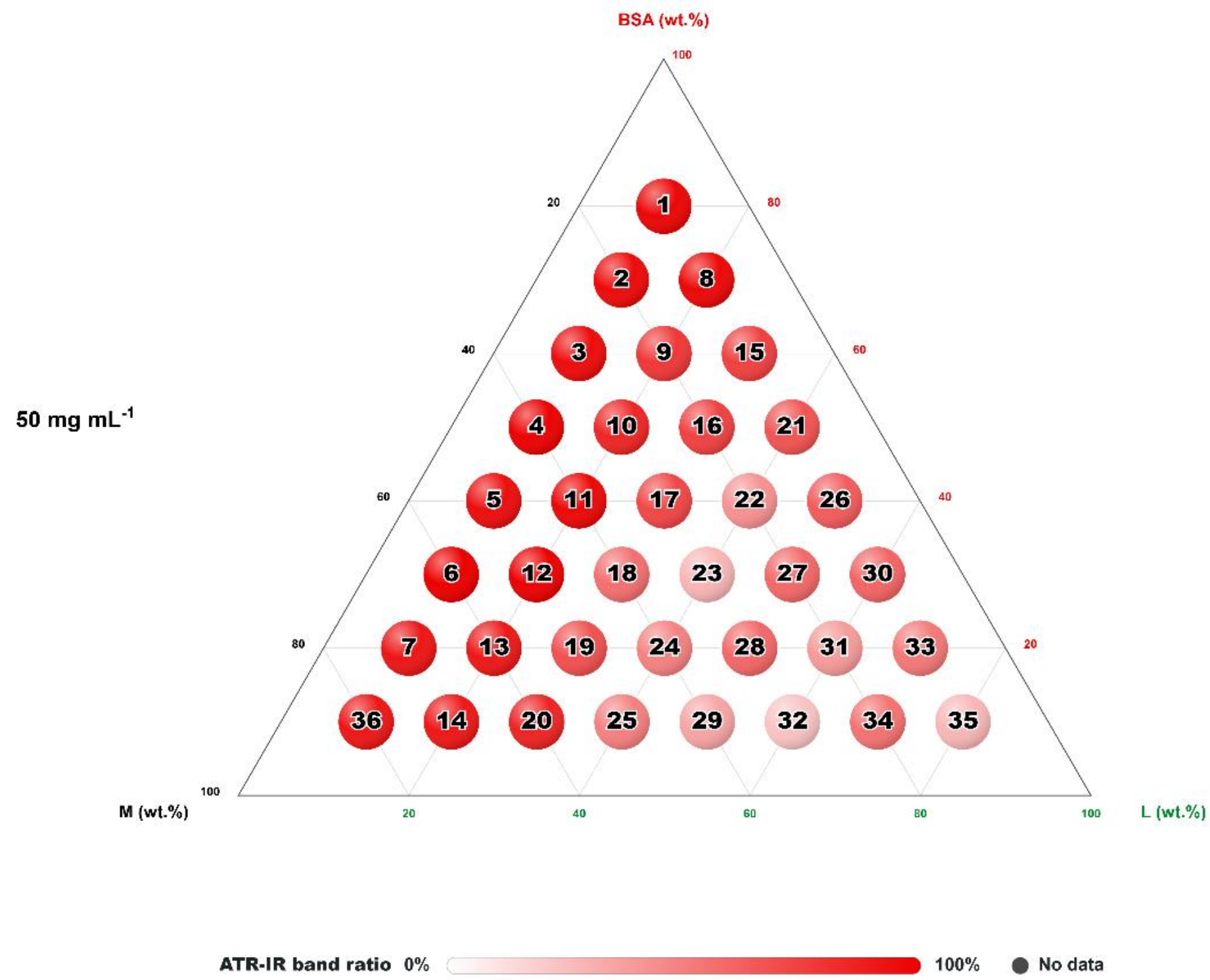

Figure S65. TD-IR ratio for the WW (TD-H_2O) system prepared at TC = 50 mg mL^{-1}.

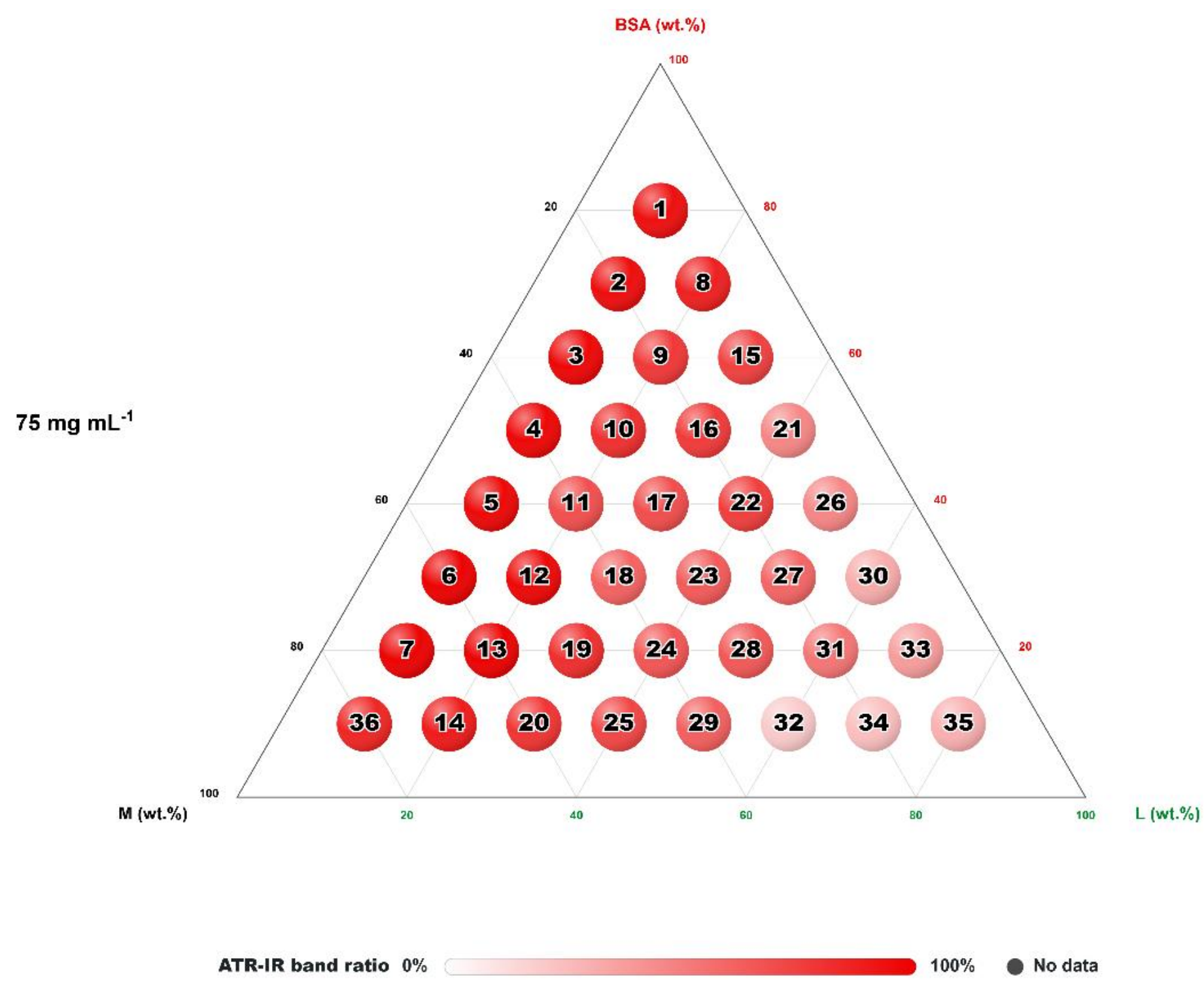

Figure S66. TD-IR ratio for the WW (TD-H_2O) system prepared at TC = 75 mg mL^{-1}.

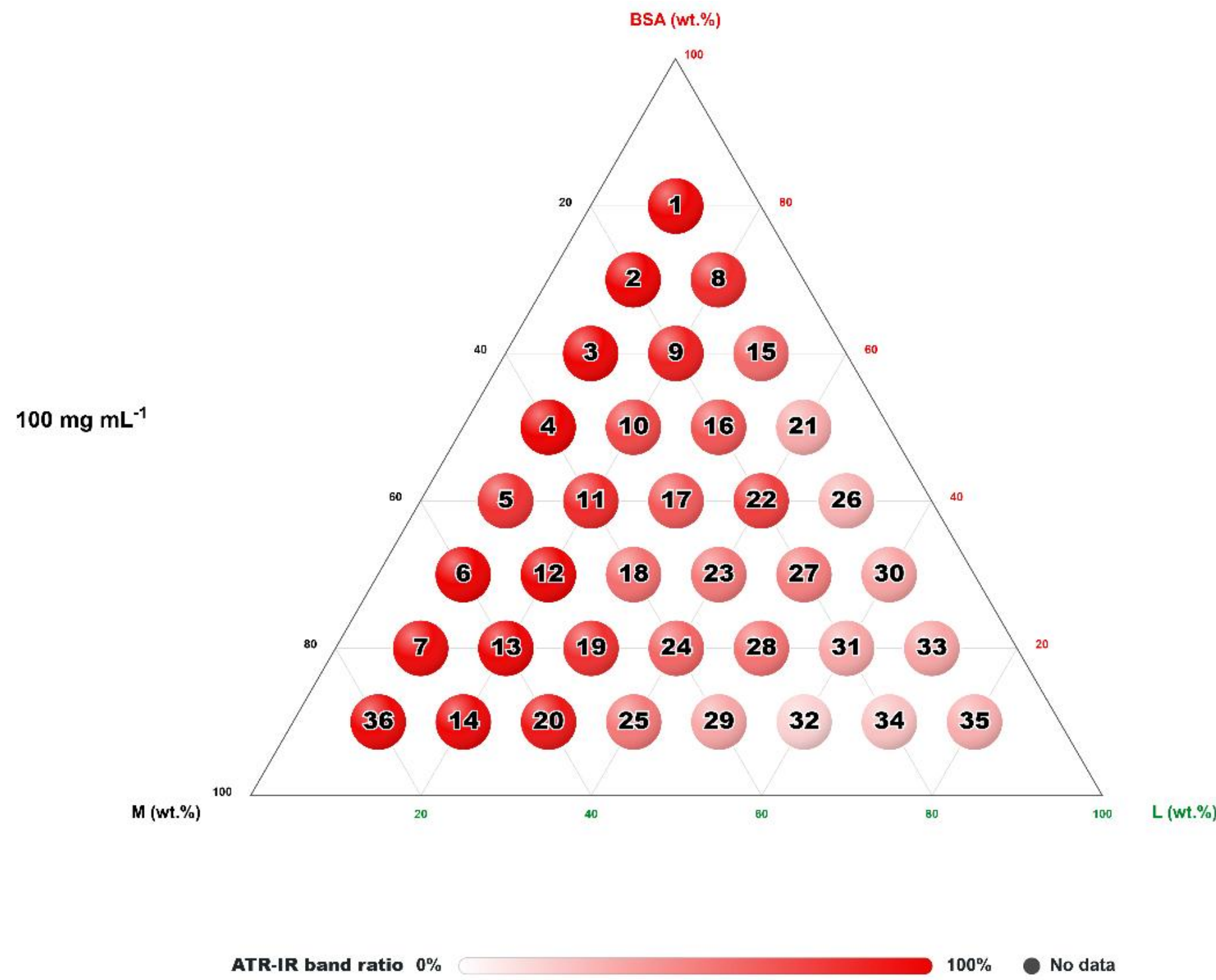

Figure S67. TD-IR ratio for the WW (TD-H_2O) system prepared at TC = 100 mg mL^{-1}.

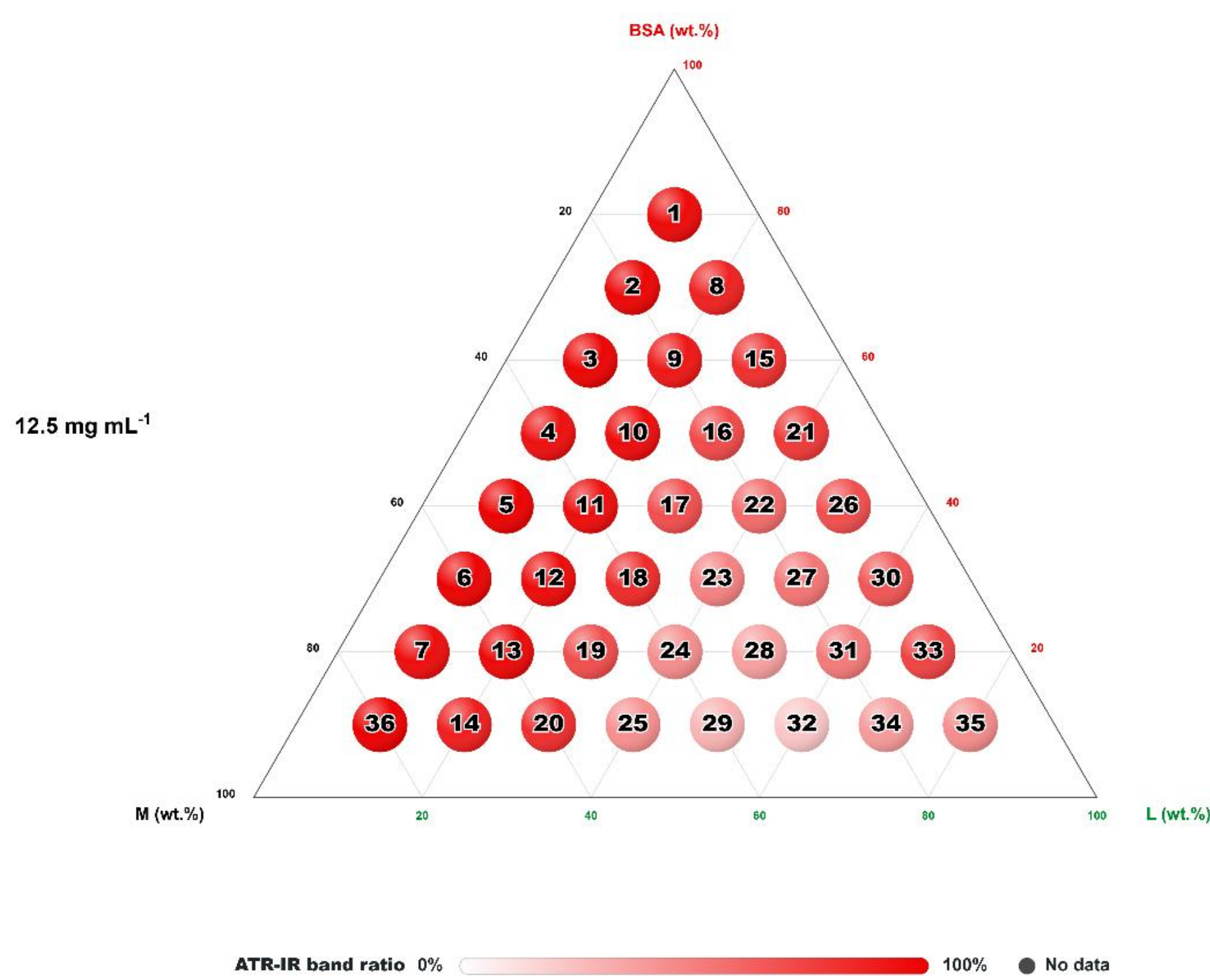

Figure S68. TD-IR ratio for EW (TD-EtOH) system prepared at TC = 12.5 mg mL^{-1}.

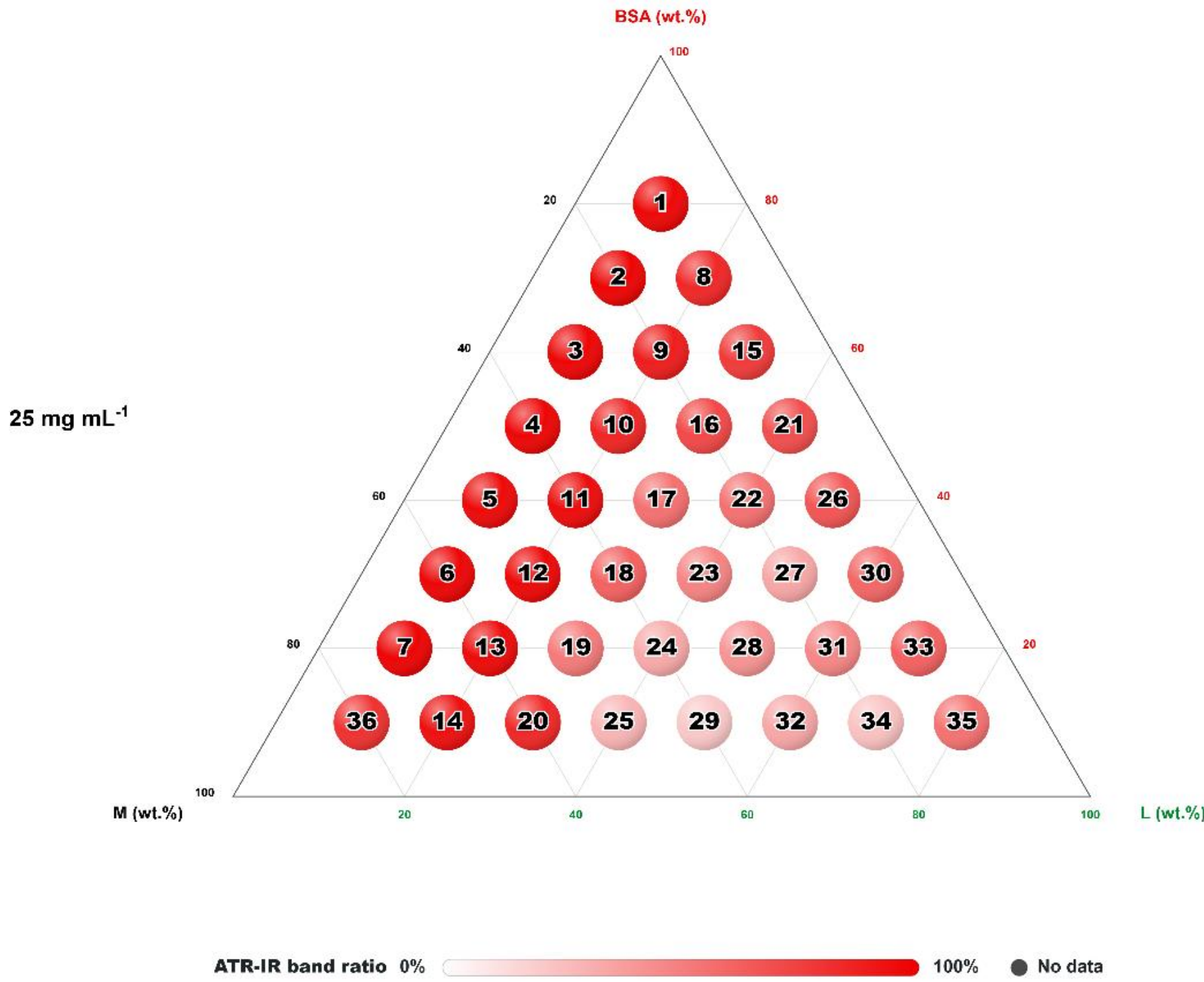

Figure S69. TD-IR ratio for EW (TD-EtOH) system prepared at TC = 25 mg mL^{-1}.

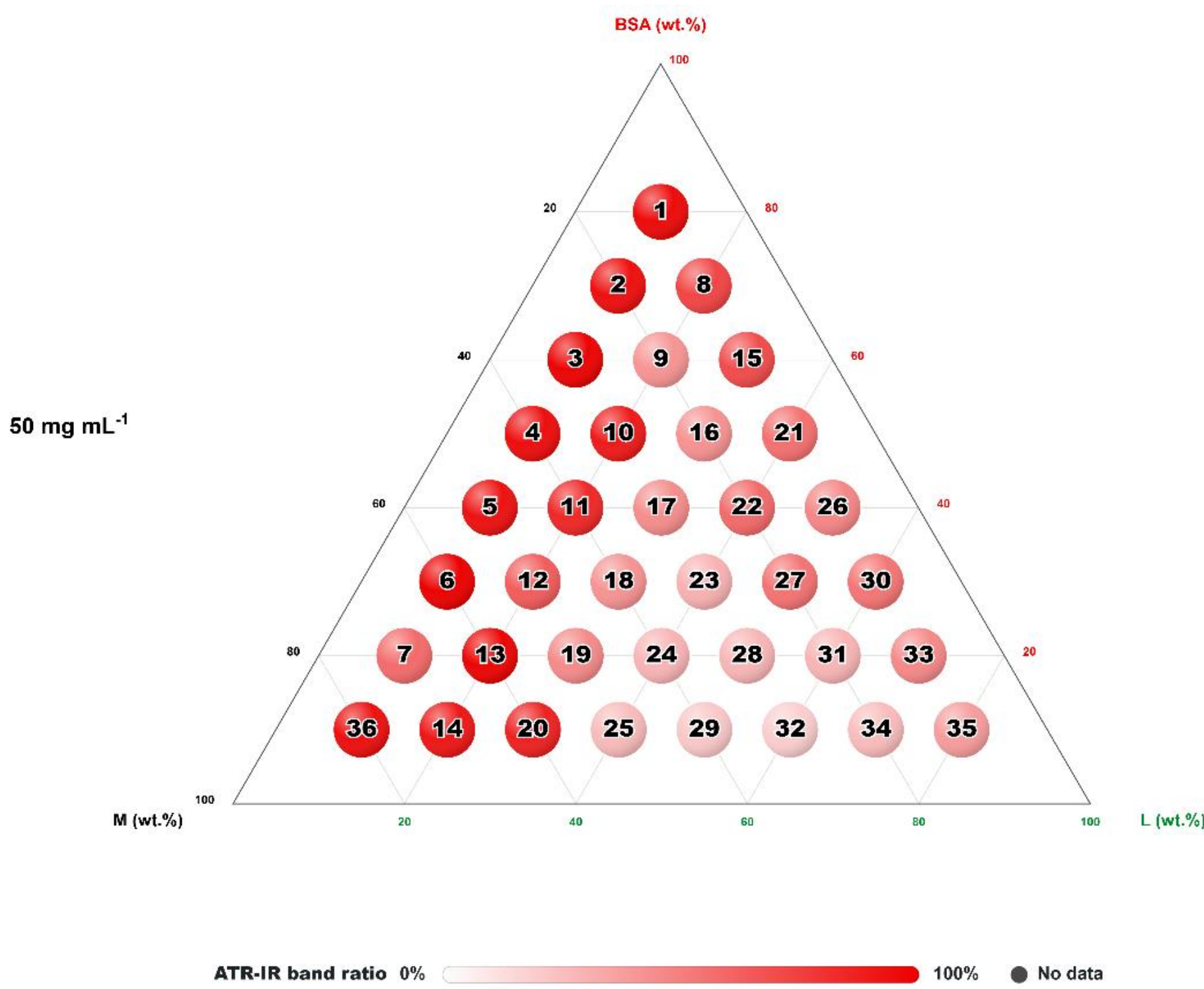

Figure S70. TD-IR ratio for EW (TD-EtOH) system prepared at TC = 50 mg mL^{-1}.

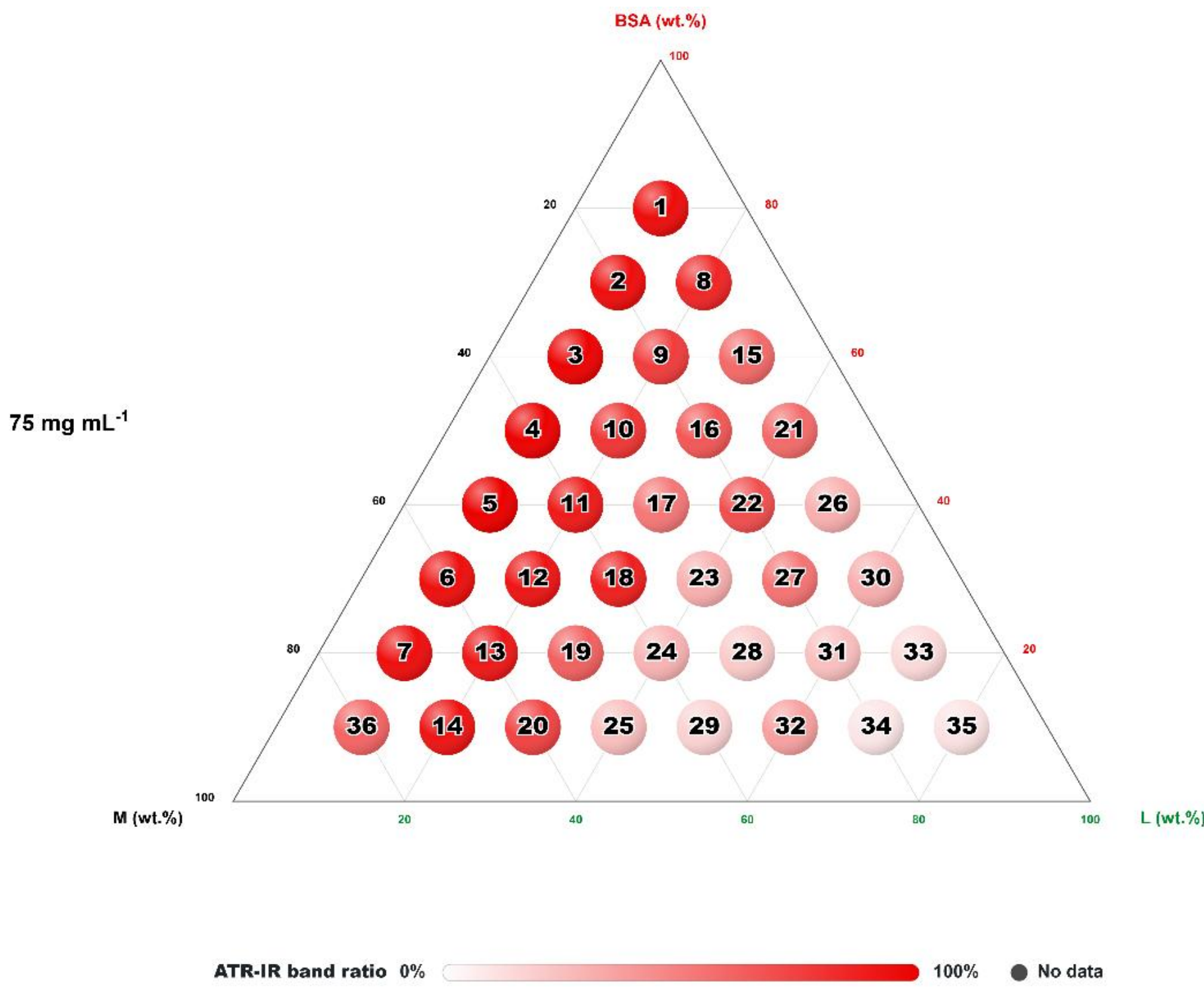

Figure S71. TD-IR ratio for EW (TD-EtOH) system prepared at TC = 75 mg mL^{-1}.

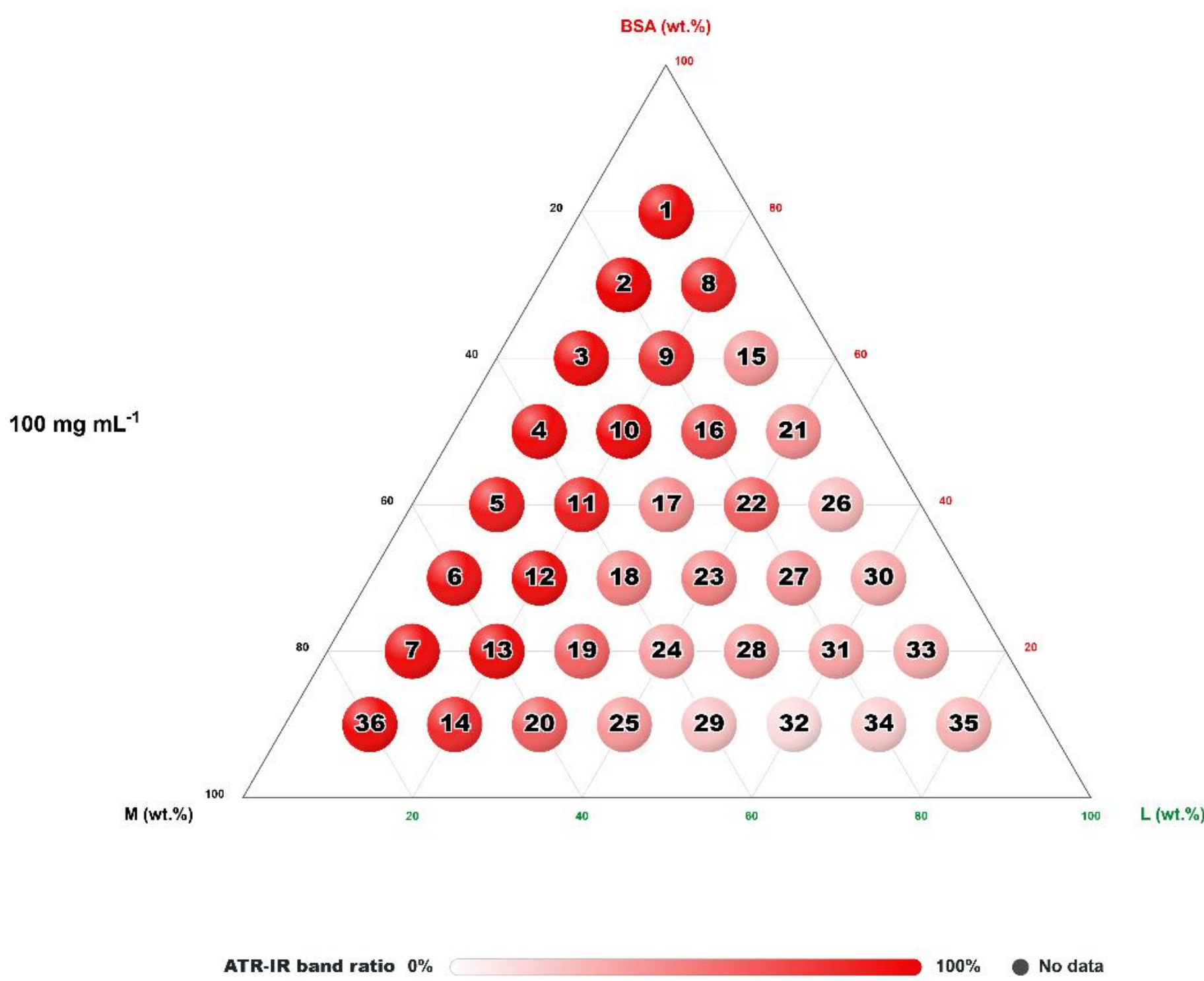

Figure S72. TD-IR ratio for EW (TD-EtOH) system prepared at TC = 100 mg mL^{-1}.

AUTHOR INFORMATION

Corresponding Author

E-mail: francesco.carraro@tugraz.at; paolo.falcaro@tugraz.at

REFERENCES

1. Kondinski, A. Automated Syringe Pump Dashboard for Multi-Reagent Dispensing in ZIF Biocomposite Synthesis, v1.0.0; *Zenodo*, **2025**. https://zenodo.org/records/20268990

2. Hafner, M. R.; Carraro, F.; Abbasgholi-NA, B.; Dal Zilio, S.; Schmallegger, M.; Wiltsche, H.; Ge, L.; Reithofer, M. R.; Astria, E.; Villanova, L.; et al. 4D Mapping of ZIF Biocomposites for High Protein Loading and Tunable Release Profiles. *Adv. Funct. Mater.* **2026**, *36* (17), e18940. https://doi.org/10.1002/adfm.202518940.

3. Hafner, M. R.; Villanova, L.; Carraro, F. App-based quantification of crystal phases and amorphous content in ZIF biocomposites. *CryEngComm.* **2022**, 24 (41), 7266–7271. https://doi.org/10.1039/d2ce00073c.

4. Liang, W.; Wied, P.; Carraro, F.; Sumby, C. J.; Nidetzky, B.; Tsung, C.-K.; Falcaro, P.; Doonan, C. J. Metal–Organic Framework-Based Enzyme Biocomposites. *Chem. Rev.* **2021**, *121* (3), 1077–1129. https://doi.org/10.1021/acs.chemrev.0c01029.

5. Niklas von Hertzen, html2canvas, Version 1.4.1, MIT License, 2022. https://html2canvas.hertzen.com/

6. Niklas von Hertzen, css-line-break, Version 2.1.0, MIT License. https://github.com/niklasvh/css-line-break

7. Niklas von Hertzen, text-segmentation, Version 1.0.3, MIT License. https://www.npmjs.com/package/text-segmentation

8. Niklas von Hertzen, utrie, Version 1.0.2, MIT License. https://github.com/niklasvh/utrie

9. Niklas von Hertzen, base64-arraybuffer, Version 1.0.2, MIT License. https://github.com/niklasvh/base64-arraybuffer
