# Supplementary material for: Semiautomated Synthesis and Interactive Visualization of Zeolitic Imidazolate Framework-Based Biocomposites
Source: Chem Mater. 2026 Sep 29;38(19):10135–48. doi: 10.1021/acs.chemmater.6c01702 (PMC13673939; doi:10.1021/acs.chemmater.6c01702)
Supplement: Supplementary file 2 [file cm6c01702_si_001.zip › Offline_ZIF-BEA/Offline_ZIF-BEA.html]

Offline\_ZIF-BEA


# ZIF Biocomposite Explorer App - Offline Version

TU Graz
Graz University of Technology

Download Page PNG


## Search

M


L


BSA

Conc.

Clear

## Dataset

TD-H_2_O
TD-EtOH

## Concentration

## Visualization

Phase
EE%
LC%
IR-ratio [%]
Amorphous fraction [%]
Framework to amorphous ratio

## Phase

## Filters

M (wt.%)


-


L (wt.%)


-


BSA (wt.%)


-


EE%


-


LC%


-


IR-ratio [%]


-


Amorphous fraction [%]


-

Reset filters

## Glossary

Composition
:   **L**: 2-methylimidazole (HmIM)
:   **M**: Zn(OAc)_2_·2H_2_O
:   **BSA**: Bovine serum albumin

Washing
:   **TD-H_2_O**: deionized water washing only
:   **TD-EtOH**: deionized water washing followed by ethanol washing

Phase
:   **ZIF-C**: Zn_2_(mIM)_2_CO_3_
:   **sod**: sodalite, Zn(mIM)_2_
:   **dia**: diamondoid, Zn(mIM)_2_

Characterization
:   **PXRD**: Powder X-ray diffraction
:   **ATR-IR**: Attenuated total reflection infrared spectroscopy

Property
:   **EE**: Encapsulation efficiency
:   **LC**: Loading capacity
:   **IR-ratio**: ATR-IR band ratio

Sample numbers


Concentrations


Axis labels


Axis numbers

Concentration size

-1x
-0.5x
1x
1.5x
2x
2.5x
3x

Axis number size

-1x
-0.5x
1x
1.5x
2x
2.5x
3x

Axis label size

-1x
-0.5x
1x
1.5x
2x
2.5x
3x

Sample circle size

-1x
-0.5x
1x
1.5x
2x
2.5x
3x

Sample number size

-1x
-0.5x
1x
1.5x
2x
2.5x
3x

Legend size

-1x
-0.5x
1x
1.5x
2x
2.5x
3x

Reset View
Download PNG

Rotate
Pan

Layer spacing

X
Y

Z
L

+
-

TD-H_2_O

100 mg mL^-1^

## Sample 34

100 mg mL^-1^
**-**

M (wt.%)
:   -

L (wt.%)
:   -

BSA (wt.%)
:   -

L/M mass ratio
:   -

EE +/-
:   -

LC
:   -

IR-ratio
:   -

Amorphous fraction +/-
:   -

Framework to amorphous ratio
:   -

| Total concentration | Phase | Framework to amorphous ratio | EE | LC | IR-ratio | Amorphous fraction |
| --- | --- | --- | --- | --- | --- | --- |

### PXRD patterns

-
CSV
PNG
Reset
-
+

### ATR-IR spectrum

-
CSV
PNG
Reset
-
+
